# Metacyclogenesis defects and gene expression hallmarks of histone deacetylase 4-deficient *Trypanosoma cruzi* cells

**DOI:** 10.1038/s41598-021-01080-1

**Published:** 2021-11-04

**Authors:** Gisele Fernanda Assine Picchi-Constante, Eloise Pavão Guerra-Slompo, Ana Carolina Tahira, Monica Visnieski Alcantara, Murilo Sena Amaral, Arthur Schveitzer Ferreira, Michel Batista, Cassiano Martin Batista, Samuel Goldenberg, Sergio Verjovski-Almeida, Nilson Ivo Tonin Zanchin

**Affiliations:** 1 Instituto Carlos Chagas, Fiocruz Paraná, Curitiba, Paraná, 81350-010 Brazil; 2 Laboratório de Parasitologia, Instituto Butantan, São Paulo, SP 05503-900 Brazil; 3 Departamento de Bioquímica, Instituto de Química, Universidade de São Paulo, São Paulo, SP 05508-900 Brazil

**Keywords:** Epigenetics, Post-translational modifications, Proteomics, Transcriptomics

## Abstract

*Trypanosoma cruzi*—the causative agent of Chagas disease—like other kinetoplastids, relies mostly on post-transcriptional mechanisms for regulation of gene expression. However, trypanosomatids undergo drastic changes in nuclear architecture and chromatin structure along their complex life cycle which, combined with a remarkable set of reversible histone post-translational modifications, indicate that chromatin is also a target for control of gene expression and differentiation signals in these organisms. Chromatin-modifying enzymes have a direct impact on gene expression programs and DNA metabolism. In this work, we have investigated the function of *T. cruzi* histone deacetylase 4 (*Tc*HDAC4). We show that, although *Tc*HDAC4 is not essential for viability, metacyclic trypomastigote *Tc*HDAC4 null mutants show a thin cell body and a round and less condensed nucleus located very close to the kinetoplast. Sixty-four acetylation sites were quantitatively evaluated, which revealed H2AT85ac, H4K10ac and H4K78ac as potential target sites of *Tc*HDAC4. Gene expression analyses identified three chromosomes with overrepresented regions of differentially expressed genes in the *Tc*HDAC4 knockout mutant compared with the wild type, showing clusters of either up or downregulated genes. The adjacent chromosomal location of some of these genes indicates that *Tc*HDAC4 participates in gene expression regulation during *T. cruzi* differentiation.

## Introduction

The protozoan parasite *Trypanosoma cruzi* undergoes drastic morphological and biochemical modifications during the different stages of its complex life cycle in invertebrate and mammalian hosts. Transition between the different stages involves dramatic alterations in gene expression. Like in other kinetoplastids, regulation of gene expression in *T. cruzi* has largely been considered to take place mostly by post-transcriptional mechanisms. However, the differentiation process of trypanosomatids is accompanied by modifications in chromatin structure and nuclear organization which has associated chromatin remodeling with gene silencing/activation mechanisms and, consequently, associated the chromatin state with the potential of gene expression^1,2^. In order to fully understand the role of chromatin in the regulation of cellular processes in trypanosomatids, it is essential to further the knowledge regarding the molecular components of the epigenetic machinery.

Chromatin-modifying enzymes are major players in epigenetic mechanisms, mediating reversible covalent modifications and having a direct impact on gene expression programs^3,4^. The histones, by their turn, are main targets of the chromatin-modifying enzymes. *T. cruzi* histones present some peculiarities, such as divergence of canonical histone sequence relative to model organisms, histone H1 lacking globular domain and Kinetoplastida-specific histone variants^5^. All *T. cruzi* canonical and variant histones are decorated with a multitude of post-translational modifications (PTMs) both at the flexible tails and in the globular region^6–11^. Some of these PTMs correlate with those described for other organisms, while others seem to be exclusive of trypanosomatids, raising interesting questions about their role in epigenetic mechanisms. Some of these PTMs have already been implicated in chromatin remodeling, playing fundamental roles in cell cycle and differentiation and influencing the adaptive response of *T. cruzi* to the different hosts^2,12^.The scores of PTMs already described for *T. cruzi* histones^6–11^ imply that a respective set of chromatin-modifying enzymes must be present in this organism. However, so far there is little information on *T. cruzi* chromatin-modifying enzymes. Therefore, in the present work, we focus on the characterization of *T. cruzi* histone deacetylase 4 (*Tc*HDAC4).

Reversible histone acetylation has been established as an important mechanism in regulation of gene expression^13,14^. Currently, there is already a wealth of studies on deacetylases in many organisms. They are usually classified according to the yeast and human orthologues in two superfamilies. The arginase/deacetylase superfamily, comprising the zinc-dependent histone deacetylase family members and, the NAD-binding domain superfamily, comprising the Sir2 regulator family members^15–17^. The zinc-dependent histone deacetylase family is further divided into Classes I (yeast Rpd3, Hos1-3 and human HDAC1-3 and HDAC8), IIa (yeast Hda1 and human HDAC4-5, HDAC7 and HDAC9), IIb (human HDAC6 and HDAC10) and IV (human HDAC11). In this classification, the Sir2 family members form the Class III (yeast Sir2, Hst1-4 and human SIRT1-7)^15–17^. Concerning trypanosomatids, four genes encoding zinc-dependent histone deacetylases (HDAC1-4) have been identified^18^. Based on sequence similarity, HDAC1 and HDAC2 were grouped in Class I and, HDAC3 and HDAC4 were grouped in Class II^18^. *T. brucei* DAC1 and DAC3 appear to be essential proteins, while DAC2 and DAC4 are not necessary for cell viability, although DAC4 mutant parasites present delayed cycle progression^18^. *T. brucei* DAC1 and DAC3 show deacetylase activity and have distinct roles in silencing variable surface glycoproteins (VSGs)^19^. Based on the particular substrate features and on their critical biochemical functions , HDACs have already been used as targets of chemotherapeutic drugs for cancer and neurodegenerative diseases^20,21^. The evidence available so far suggests that HDACs are key enzymes also in trypanosomatids, representing excellent targets for the development of chemotherapy drugs^22,23^.

Despite being considered orthologs, HDACs may not have the same roles in *T. brucei*, *T. cruzi* and *Leishmania* parasites, driving the urgent need for studies to uncover the specific mechanism of action for each of them^24^. This work focuses on *Tc*HDAC4 role in cell division, regulation of gene expression and histone post-translational modifications. Although *Tc*HDAC4 is not essential for *T. cruzi* viability, metacyclic trypomastigotes deficient in *Tc*HDAC4 present abnormal cell morphology, showing a very thin cell body with the kinetoplast and nucleus located very closely to each other. Furthermore, global gene expression changes in *Tc*HDAC4 knockout parasites showed that *Tc*HDAC4 participates in gene expression regulation. Histone post-translational modification analyses with quantitative mass spectrometry showed changes in acetylation of specific histone residues in *Tc*HDAC4 knockout parasites. Overall, our work indicates that *Tc*HDAC4 plays a role in the *T. cruzi* differentiation process.

## Results

### *Tc*HDAC4 is a divergent deacetylase expressed mainly in the insect-equivalent stages of the cell cycle

The *Trypanosoma cruzi* genome encodes four highly divergent histone deacetylase genes, two of them belonging to Class I (*Tc*HDAC1 and 2) and two (*Tc*HDAC3 and 4) to Class II. The deacetylase domains of *Tc*HDAC1 and *Tc*HDAC2 share only 31% amino acid identity with each other and, respectively, 49% and 38% with *Homo sapiens* HDAC1 (NP_004955.2) which is the closest human ortholog for both (Fig. S1A). The deacetylase domains of *Tc*HDAC3 and *Tc*HDAC4 share only 33% of amino acid identity, both presenting a large number of insertions (Fig. S1B). All four *T. cruzi* HDACs contain the zinc-binding residues and the catalytic tyrosine in their catalytic domain (Fig. S1A, S1B).

**Figure 1:**
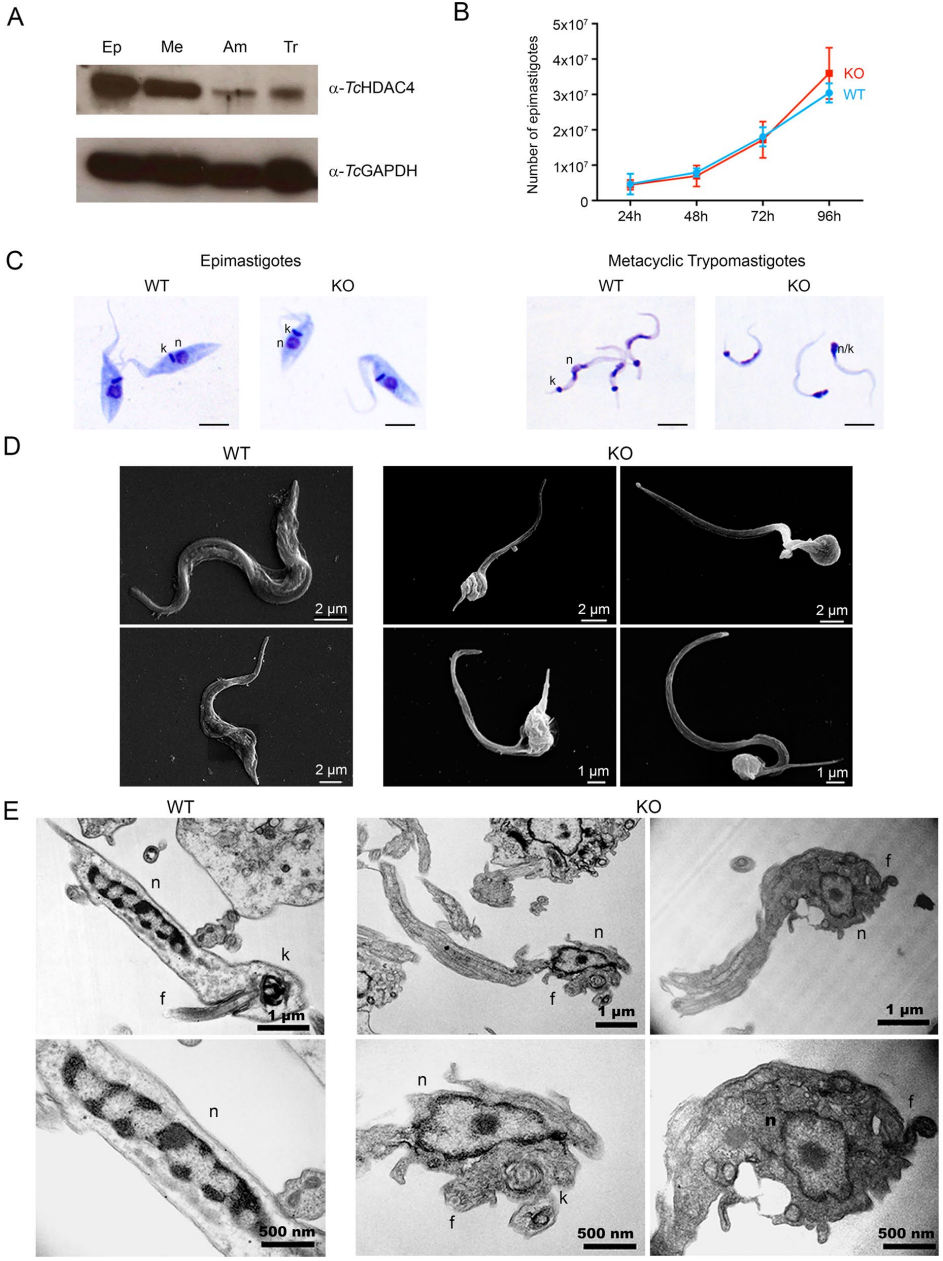
*Tc*HDAC4 is expressed throughout the *T. cruzi* life cycle with higher levels present in the insect-equivalent stages of the life cycle reproduced in vitro and its knockout affects cell morphology during metacyclogenesis. (**A**) Immunoblot using anti- *Tc*HDAC4 serum in cell extracts from epimastigote (Ep), metacyclic trypomastigote (Me), amastigote (Am) and cell culture-derived trypomastigote (Tr) cells. α-*Tc*GAPDH was used as a loading control. Only the part of the immunoblot corresponding to position of the *Tc*HDAC4 and *Tc*GAPDH bands is shown. Complete image of the western blot is presented in Supplementary Figure CI-1. (**B**) Proliferation curves obtained from biological triplicates. (**C**) Panoptic staining of epimastigote and metacyclic trypomastigote cells from wild type (WT) and *Tc*HDAC4 null mutant (KO) cells. Scale bars = 5 µm. (**D**) Scanning electron microscopy of metacyclic trypomastigote from wild type (WT) and *Tc*HDAC4 null mutant (KO) cells. (**E**) Transmission electron microscopy of metacyclic trypomastigotes from wild type (WT) and *Tc*HDAC4 null mutant (KO) cells. The lower panels correspond to larger magnification of the nuclear region of the same cells shown in the upper panel. n: nucleus, k: kinetoplast, f: flagellum.

*Tc*HDAC4 presents only 40% amino acid identity with its closest human ortholog (AAH69243.1) and the HDAC4 orthologs are also highly divergent among trypanosomatids (Fig. S2). However, despite the low overall sequence conservation, the presence of the catalytic and zinc binding residues indicates that the *TcHDAC4* gene encodes a functional deacetylase (Fig. S2). Initially, we have evaluated *Tc*HDAC4 expression in different stages of the *T. cruzi* life cycle reproduced in vitro. *Tc*HDAC4 is expressed in all four stages tested (Fig. 1A) with higher expression levels occurring in the ones that represent the insect stages (epimastigote and metacyclic trypomastigote), while lower levels are observed in the stages that occur within the mammalian host (amastigote and cellular trypomastigote) (Fig. 1A).

### Knockout of *Tc*HDAC4 affects *T. cruzi* cell morphology during differentiation in vitro

To investigate *Tc*HDAC4 function, we have used a gene deletion strategy to obtain double *TcHDAC4* knockout cells (Fig. S3). Surprisingly, in epimastigote cells *Tc*HDAC4 knockout had no effect on the proliferation rate (Fig. 1B) neither on the morphology (Fig. 1C, left WT and KO panels). However, initial microscopic analyses revealed intriguing morphological differences between WT and KO metacyclic trypomastigotes obtained from differentiation in vitro. In most null mutant metacyclic trypomastigotes, the kinetoplast and the nucleus are located very closely to each other, and the cell body is thinner than in metacyclic trypomastigotes derived from wild type cells (Fig. 1C, right WT and KO panels). The distinct body of the null mutant metacyclic trypomastigote cells can be nicely observed in the scanning electron microscopy images (Fig. 1D). Indeed, ultrastructural analyses by transmission electron microscopy (Fig. 1E) revealed a round and less condensed nucleus with a central nucleolus in KO metacyclic trypomastigotes, while the WT metacyclic trypomastigotes showed an elongated nucleus with condensed peripherical heterochromatin patches. Moreover, unlike WT metacyclic trypomastigotes that present the kinetoplast at the posterior body end, in null mutants the kinetoplast is parallel to the nucleus. As seen in the scanning microscopy images, the cell body of the KO cells is slender and appears to have long tubular structures, not observed in WT metacyclic trypomastigotes, which suggests cytoskeleton disorganization. The less condensed nucleus with a central nucleolus of the KO metacyclic trypomastigotes keeps some structural resemblance with the nucleus of wild type epimastigote cells and may be a direct consequence of the lack of *Tc*HDAC4 activity in the KO cells.

### Knockout of *Tc*HDAC4 compromises the infection potential of metacyclic trypomastigotes differentiated in vitro

**Figure 2:**
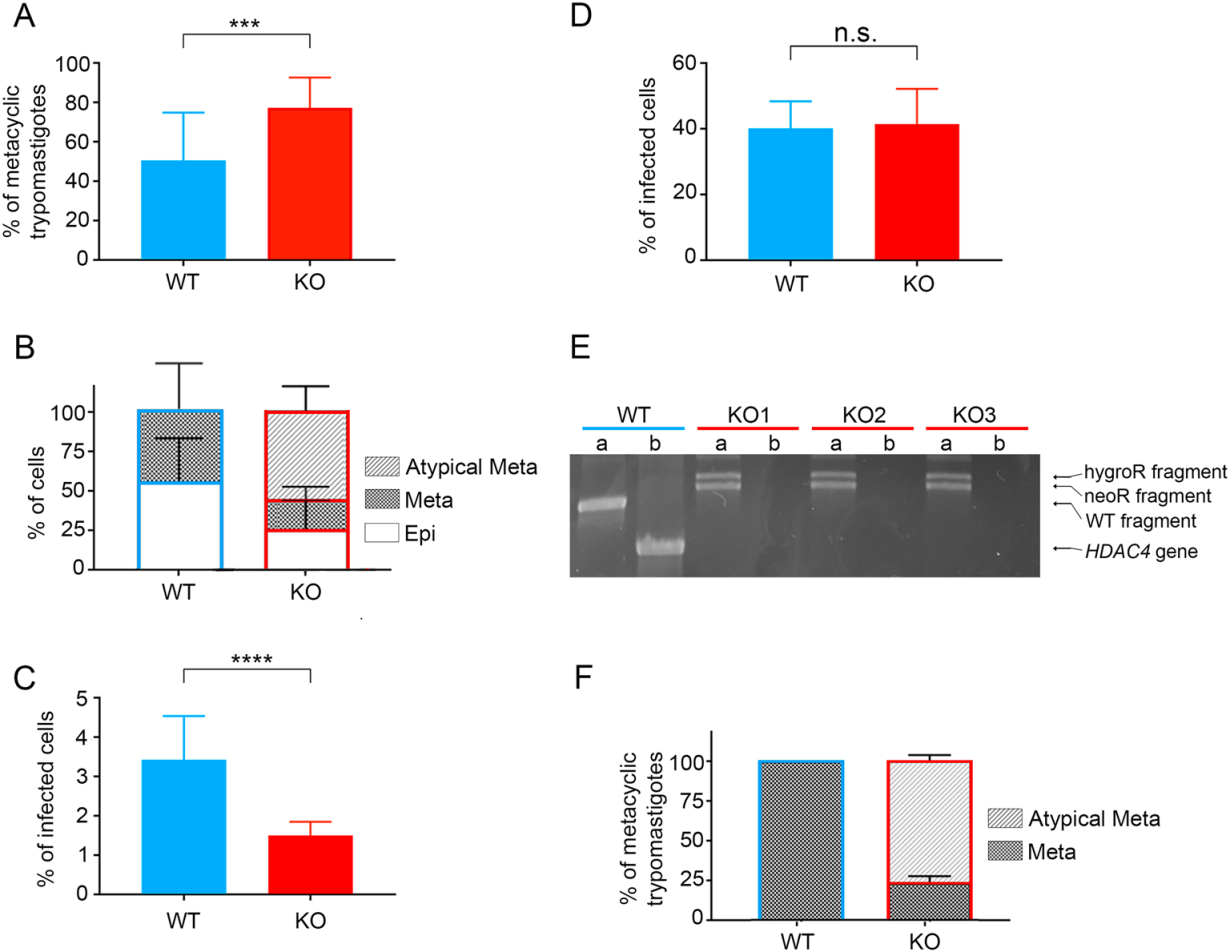
Knockout of *Tc*HDAC4 affects *T. cruzi* differentiation and infection processes. (**A**) Percentage of metacyclic trypomastigotes after 72 h differentiation. WT data are from 4 biological replicas and KO data from 10 biological replicas, all including at least 3 technical replicates each. ***indicates p-value < 0.0002. (**B**) Percentage of epimastigotes (Epi) and normal and atypical metacyclic trypomastigotes (Meta and Atypical Meta, respectively). WT data are from 3 biological replicas and KO data from 9 biological replicas, including at least 3 technical replicates each. (**C**) Percentage of cells infected with metacyclic trypomastigote differentiated in vitro. WT data are from 2 biological replicas and KO data from 4 biological replicas, all including at least 3 technical replicates each. ****indicates p-value < 0.0001. (**D**) Percentage of cells infected with cell-derived trypomastigotes. WT data are from 2 biological replicas with 20 technical replicates and KO data are from 6 biological replicas with 5 technical replicas each. (**E**) Amplification profile of DNA fragments including the knockout cassette insertion (a) and the *TcHDAC4* gene (b) from wild type and 3 clones of null mutant parasites. In the KO cells, there is no PCR product for the *TcHDAC4* gene whereas the knockout cassettes were amplified, confirming the genotype of the *TcHDAC4* KO cells. Complete image of the DNA gel electrophoresis is presented in Supplementary Figure CI-2. (**F**) Percentage of normal and atypical metacyclic trypomastigotes (Meta and Atypical Meta, respectively) from the metacyclogenesis in vitro performed after the epimastigogenesis. WT data are from 3 biological replicates and KO from 6 biological replicates.

Although the *Tc*HDAC4 null mutant proliferates at the same rate as wild type parasites (Fig. 1A), the differentiation process is impacted in these cells, which intriguingly results in a significant increase of rate of metacyclic trypomastigote formation (Fig. 2A). However, approximately 75% of the differentiated null mutants metacyclic trypomastigotes present altered morphological phenotype (Fig. 2B). The question whether the metacyclic trypomastigotes derived from *Tc*HDAC4 KO cell keep their infective potential was addressed by performing a Vero cell infection assay using freshly metacyclic trypomastigotes differentiated in vitro. In this experiment, the infection rate of the null mutants was significantly reduced relative to the rate of wild type counterparts (Fig. 2C). Since it is not possible to separate the atypical metacyclic trypomastigotes from the regular ones during isolation of the metacyclic trypomastigotes from null mutant cells, it was not possible to determine whether the defective metacyclic trypomastigotes can indeed infect cells. One possibility is that the metacyclic trypomastigotes showing normal cellular conformation are infective despite lacking *Tc*HDAC4. Unexpectedly, null mutant trypomastigotes released from Vero cells previously infected with metacyclic trypomastigotes obtained from differentiation in vitro show normal morphology and present infection rates similar to the wild type trypomastigotes (Fig. 2D). The genotype of the cell-derived null mutant trypomastigotes was confirmed by PCR amplification of the specific gene locus, which consistently confirmed the absence of the *TcHDAC4* gene and the presence of the knockout cassettes (Fig. 2E). A second control experiment was performed using cell-derived trypomastigotes, which were subjected to epimastigogenesis in vitro to obtain epimastigotes and submitted to another round of metacyclogenesis in vitro. Consistently, the resulting differentiation profile was similar to the one described above in which about 75% of obtained metacyclic trypomastigotes from null mutant parasites presented altered nuclear morphology (Fig. 2F).

### Dynamic changes in gene expression in the development of Dm28c

In order to investigate the impact of *Tc*HDAC4 knockout on gene expression along the *T. cruzi* life cycle, we have performed RNA-Seq analysis from wild type and *Tc*HDAC4 knockout cells obtained from different stages of the metacyclogenesis in vitro. Initially, we have analyzed the differentially expressed genes (DEGs) during metacyclogenesis using only wild type samples, which resulted in 1,213 DEGs (FDR < 10^–3^, log_2_FC ≥ 1.5) among the different developmental stages (Fig. S4A). Part of these genes was also identified as differentially expressed in previous studies. Santos and collaborators identified 1,791 DEGs (FDR < 0.1) in the polyA + RNA fraction of the CL-Brener strain which were modulated during a 10-day period of growth from log to stationary phase^25^. A comparison of these two studies revealed an overlap of 244 DEGs (20% of 1,213, Fig. S4B). A low number of overlapping genes was expected since these studies had different approaches. Santos et al.^25^ focused on the replication time along the growth curve, without separation of the different populations, whereas the present study analyzed the differentiation process from epimastigotes to metacyclic trypomastigotes. The overlapping genes identified regarding the stressed epimastigotes versus epimastigote was 196 genes (80% concordance), with 126 up-regulated and 70 down-regulated, and 48 discordant. 24 h adhered parasites versus epimastigotes showed 109 overlapping genes (45% concordance), with 57 up-regulated, 52 down-regulated and 135 discordant. The divergent pattern is also observed when comparing trypomastigotes versus epimastigotes with 137 (56%) discordant and only 107 (44%) in concordance, being 34 up-regulated and 73 down-regulated, respectively (Fig. S4C).

A more appropriated comparison of the gene expression profile of metacyclic trypomastigotes and epimastigotes was made using the study published by Smircich and collaborators^26^ , which identified 2,744 (FDR < 0.05) genes differentially expressed between these stages using poliA + RNA fraction. These genes corresponded to 3,337 genes in the Dm28c 2018 updated annotation according to the CL-Brener reference strain. A comparison of these genes with the 1,213 DEGs found here, showed an overlap of 432 genes in Dm28c 2018 (36% of 1,213, Fig. S4B) with 421 (97%) in accordance, being 330 down-regulated and 91 up-regulated genes and only a small part discordant (11 genes, 3%) (Fig. S4C).

### Effects on global gene expression after TcHDAC4 knockout

The transcriptome-wide effect of *TcHDAC4* knockout was assessed by comparing the gene expression pattern of wild type with *Tc*HDAC4 knockout cells during the different stages of metacyclogenesis in vitro. As expected, the *TcHDAC4* knockout samples at all analyzed stages showed that most of the differentially expressed genes are upregulated in null mutant compared with wild type cells (FDR ≤ 0.05) (Table 1 and Tables S1-S4 for details of all differentially expressed genes).

**Table 1:** Summary of genes differentially expressed in *TcHDAC4* knockout samples at each developmental stage.

Developmental stage	# differentially expressed genes	Up regulated in KO	Down regulated in KO	Ratio (up/down)
Epimastigote*	114	86	28	3.07
Stressed epimastigote*	52	30	22	1.36
24 h Adhered differentiating*	167	128	39	3.28
Metacyclic trypomastigote*	236	156	80	1.95

*FDR ≤ 5%.

**Figure 3:**
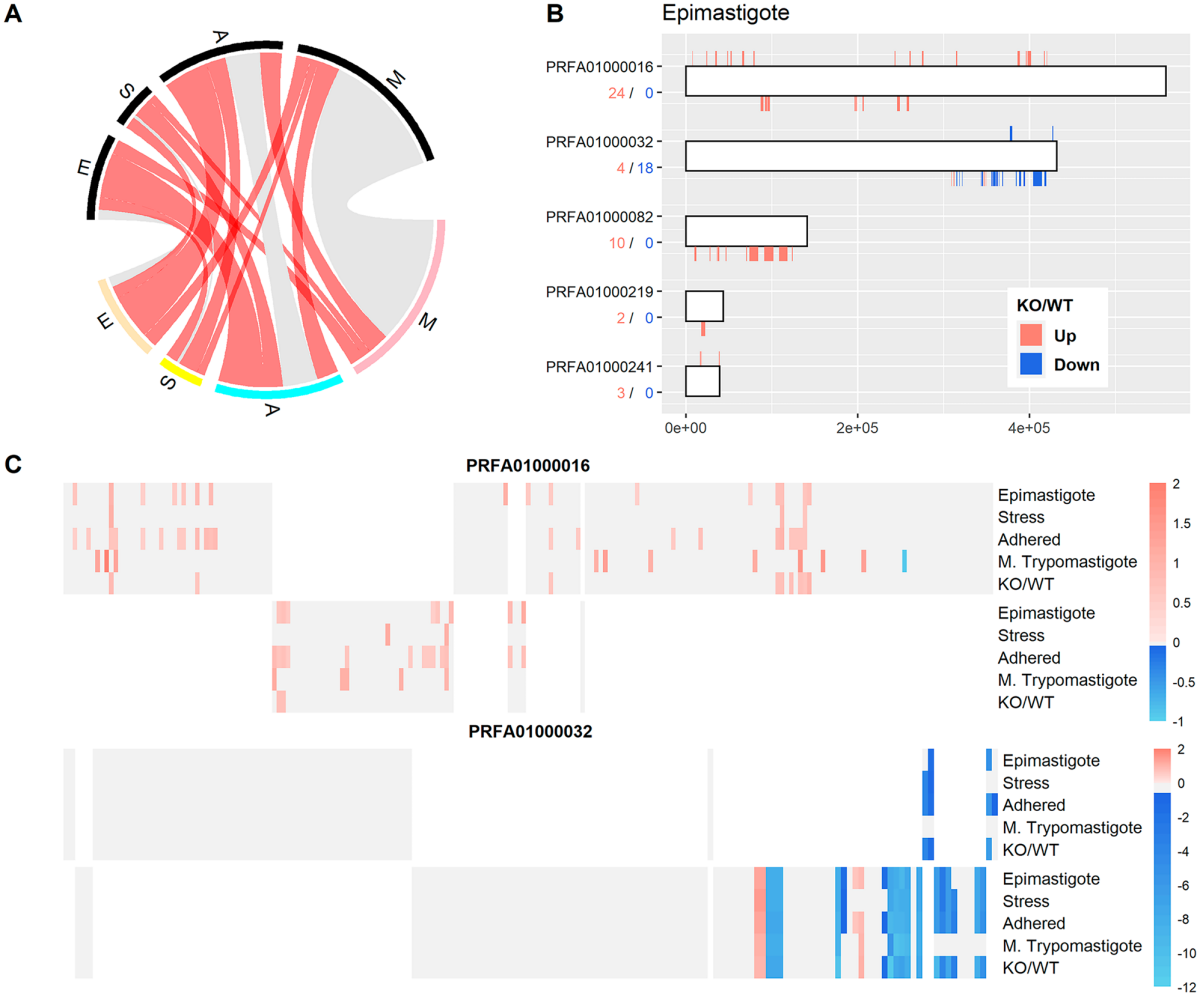
Differentially expressed genes in *TcHDAC4* knockout mutants. (**A**) Circos plot of genes differentially expressed in the comparison KO/WT at each developmental stage indicated by the letters: E, Epimastigote (orange and black); S, Stressed epimastigotes (yellow and black); A, 24 h Adhered differentiating forms (cyan and black); M, Metacyclic Trypomastigote (pink and black). Red lines represent differentially expressed genes (KO/WT) that are shared by least two developmental stages and grey lines show genes that were identified as differentially expressed only in one developmental stage. (**B**) Clusters of genes in the top five chromosomes enriched with overrepresented regions of differentially expressed genes. Bars represent the chromosome scaled according to its size. Only genes that were identified as differentially expressed are marked as red bars or blue bars, which means up and down regulation (KO/WT), respectively; colored bars that are above the chromosome indicate the genes that are encoded in the plus strand and colored bars underneath the chromosome represent genes encoded in the minus strand. (**C**) Clusters of genes from all five comparisons along chromosomes PRFA01000016 and PRFA01000032. Each heatmap shows a chromosome and is grouped in 2 panels. The upper panel represents the genes in the plus strand and the bottom panel genes in the minus strand. Each column shows a gene, and each line shows a comparison of KO/WT at the indicated developmental stage or an overall comparison of KO/WT combining all developmental stages. Differentially expressed genes are represented by red and blue colors (z-score scale at right), which indicates up and down regulation (KO/WT), respectively. Genes that were not differentially expressed are represented in grey and absence of genes is represented in white.

The number of overlapping genes that were regulated in common among all developmental stages in the *TcHDAC4* knockout samples is low, namely 13 genes representing 3% of all differentially expressed genes. Notably, metacyclic trypomastigotes exhibited a large number of genes modulated in the knockout cells (200 genes, or 85%), only at this specific stage (Fig. 3A pink, grey lines) while the other developmental stages showed 20 (4.88%), 5 (1.27%) and 56 (14.21%) genes specifically modulated only in the knockout of epimastigotes (Fig. 3A orange, grey lines), stressed epimastigotes (Fig. 3A yellow, grey lines) and 24 h adhered differentiating forms (Fig. 3A cyan, grey lines), respectively. This suggests that the *TcHDAC4* knockout tends to modulate a core set of genes (Fig. 3A). Thus, we decided to investigate if these genes were arranged in clusters inside chromosomal regions, since the *T. cruzi* genome is transcribed by polycistronic mechanisms.

First, we identified chromosomes enriched with overrepresented regions of differentially expressed genes in knockout parasites in all developmental stages (Fisher’s test, corrected by FDR ≤ 0.05). In this analysis, there were 16 chromosomes with such overrepresented regions in epimastigote (Fig. 3B: for the top five chromosomes and Fig. S5: for all chromosomes), 6 in stressed epimastigotes, 16 in 24 h differentiating forms and 11 in metacyclic trypomastigote (Figs. S6–S8 and Table S5). Combining all analyses regarding the different stages, three chromosomes with regions enriched with differentially expressed genes in knockout parasites were identified in common (PRFA01000082, PRFA01000032 and PRFA01000016). An analysis using the stages as adjustment variables and the condition (wild type and KO) as the variable of interest, identified 74 differentially expressed genes (FDR < 0.05) in common among all stages, with 46 up-regulated and 28 down-regulated in the *Tc*HDAC4 knockout mutant compared with wild type (Fig. S9 and Table S6). It is noteworthy that the consistent changes in gene expression at these chromosomal regions throughout all the analyses (Fig. 3C) pointed to chromosomal locations where the function of *Tc*HDAC4 would be required for proper gene expression.

### Post translational modification (PTM) profile of *T. cruzi* histones

Considering that *Tc*HDAC4 possesses a conserved deacetylase domain which may act directly on histones, we have evaluated the histone posttranslational modifications in wild type and *Tc*HDAC4 knockout cells by mass spectrometry. For this purpose, histone enriched preparations were obtained from four different *Tc*HDAC4 knockout clones and four replicates of the parental *T. cruzi* strain from the epimastigote and metacyclic trypomastigote stages. All *T. cruzi* canonical (H2A, H2B, H3 and H4), variant (H2AZ, H2BV and H3V) and linker (H1) histones were identified in the mass spectrometry analysis, presenting similar sequence coverage between all sample groups (Table S7).

**Figure 4:**
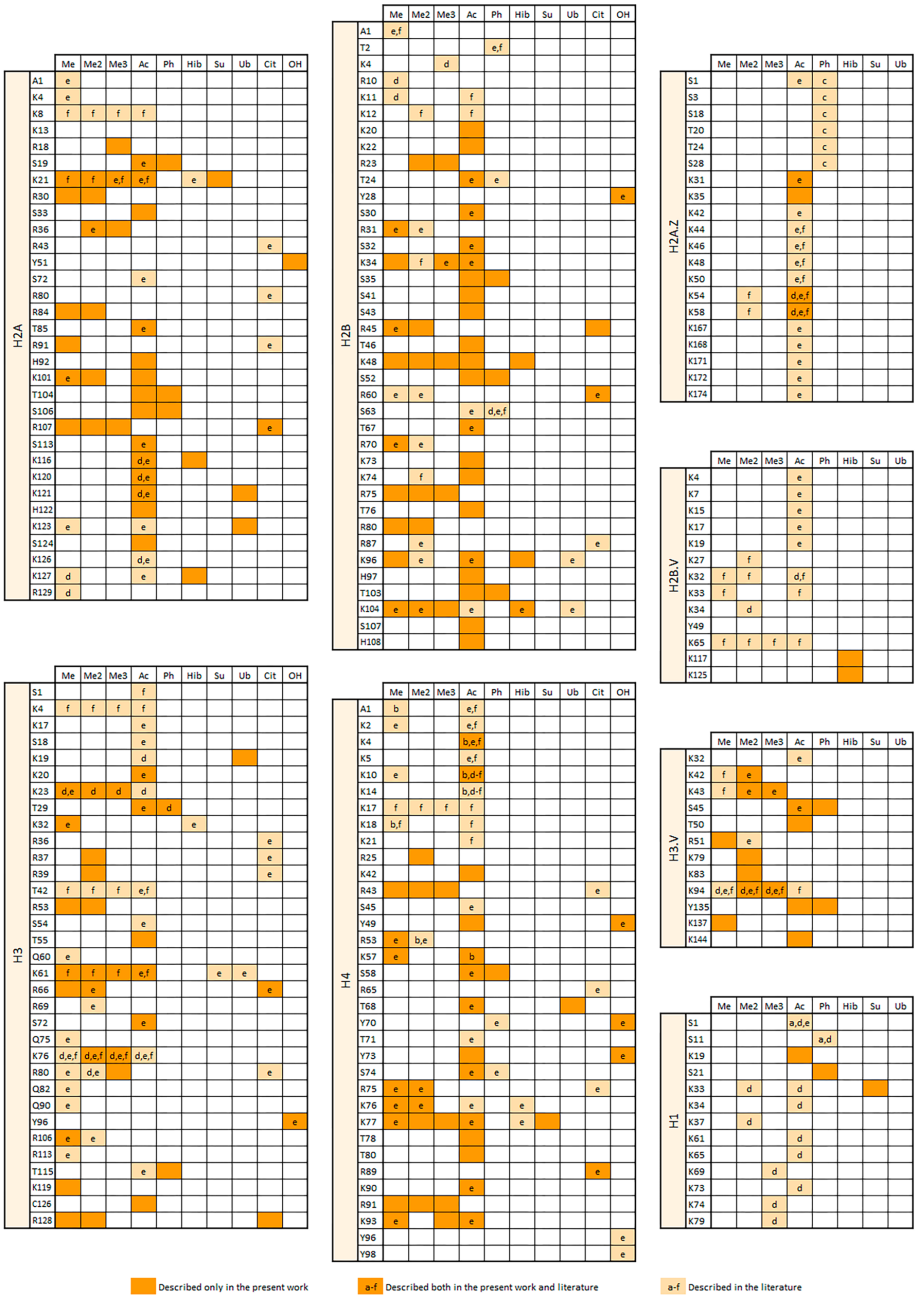
PTM sites identified in the present work and previously reported in the literature (a- da Cunha et al., 2005^6^; b- da Cunha et al., 2006^7^; c- Marchini et al., 2011^8^; d- de Jesus et al., 2016^9^; e- Picchi et al., 2017^11^ and f- de Lima et al., 2020^10^). Me: methylation, Me2: dimethylation; Me3: trimethylation; Ac: acetylation; Ph: phosphorylation; Hib: 2-hydroxyisobutyrylation; Su: succinylation; Ub: ubiquitination; Cit: citronylation; OH: hydroxylation. Noteworthy, as described for humans and other eukaryotes, *T. cruzi* histones lose the initial methionine during protein processing.

A total of 185 post translational modifications (PTM) sites were identified distributed along all the *T. cruzi* histones. 161 of these sites were found in canonical histones while 24 in the variants and linker histones (Fig. 4, Table S8). Sites of lysine formylation and propionylation were also identified but they were not considered for the analysis because it is not possible to differentiate the native sites from the ones originating from the formic acid present during liquid chromatography and from the propionic anhydride treatment used for sample preparation.

### Evaluation of the histone post-translational modification potential targets of *Tc*HDAC4 regulation

**Figure 5:**
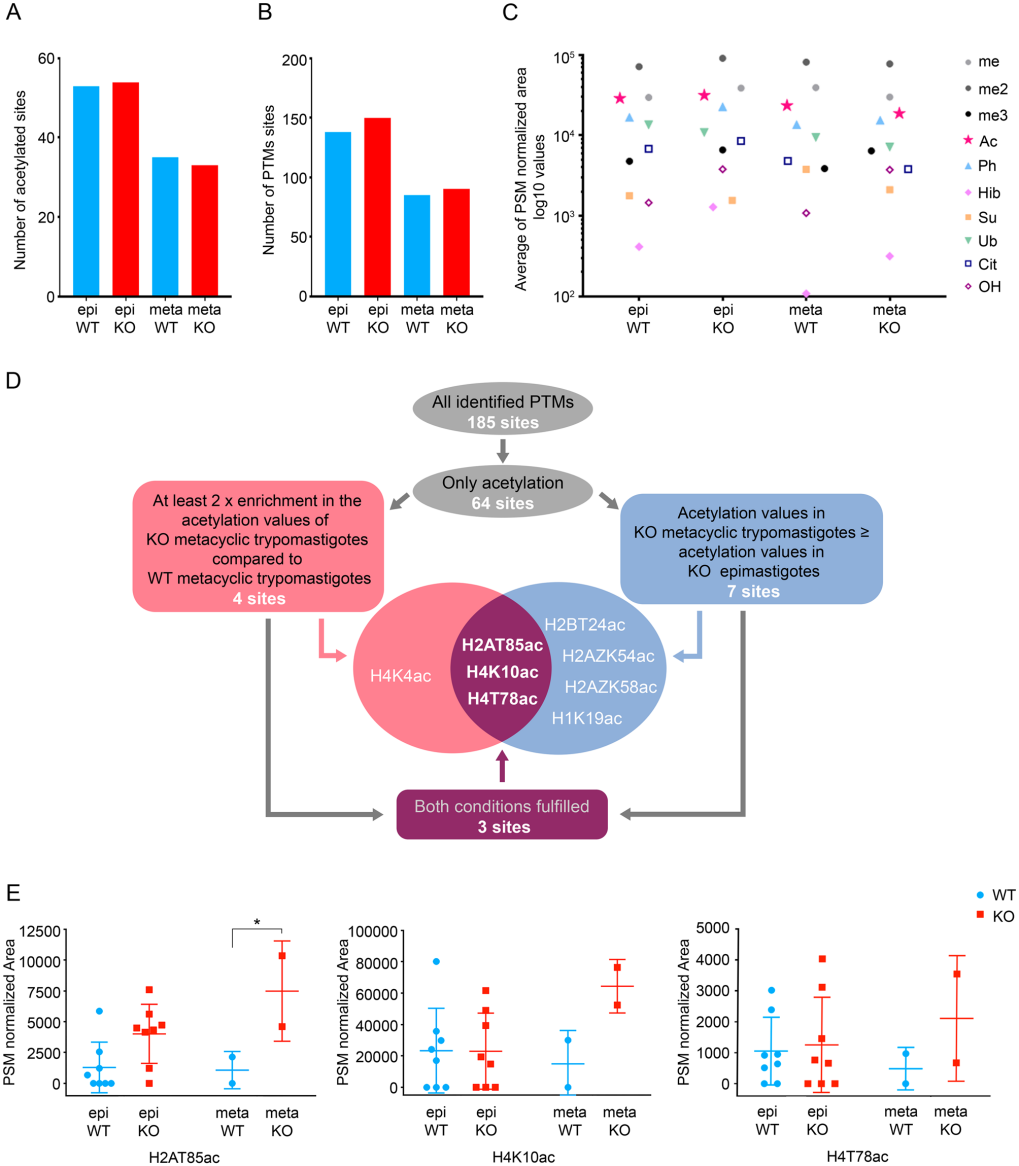
*Tc*HDAC4 histone post-translational modifications targets. (**A**) Number of distinct acetylation sites identified for each condition. (**B**) Numbers of distinct PTMs identified for each condition. (**C)** Comparison of the global PTM levels in epimastigotes (epi) and metacyclic trypomastigotes (meta) from wild type (WT) and mutant (KO) cells using the average of the peptide intensity (me, monomethylation; me2, dimethylation; me3 trymethylation; Ac, acetylation; Ph, phosphorylation; Hib: 2-hydroxyisobutyrylation; Su: succinylation; Ub: ubiquitination; Cit: citronylation; OH: hydroxylation). (**D**) Flowchart describing the steps to determine the potential acetylation sites modulated by *Tc*HDAC4 in *T. cruzi* metacyclic trypomastigotes. (**E**) Acetylation levels of the putative *Tc*HDAC4 specific target sites. The graph shows the PSM normalized area of the peptide intensity for the peptides containing acetylated threonine 85 in histone H2A (H2AT85Ac), acetylated lysine 10 in histone H4 (HK410Ac) and acetylated threonine 78 in histone H4 (H4T78ac). *indicates p value = 0.0296.

For the epimastigote stage, the total modified sites identified was 138 for the WT and 150 for the *Tc*HDAC4 knockout cells. For the metacyclic trypomastigote stage, 85 PTMs sites were identified for the WT and 90 for the knockout cells. Concerning acetylation, the total number of sites identified for the epimastigote stage was 53 for the WT and 54 for the knockout cells and, for the metacyclic trypomastigote stage the number of sites identified was 35 for WT and 33 for knockout cells (Fig. 5A). In both stages, the number of PTMs sites for WT and knockout cells is similar, indicating that there is no easily detectable global change of histone PTM pattern in *Tc*HDAC4 knockout cells (Fig. 5B). Therefore, we performed a comparative quantitative analysis.

For the quantitative analysis, a single representative sequence chosen by the highest coverage index was used for each *T. cruzi* histone (see “Methods”). The chromatographic peak area of each modified peptide feature was normalized to the PSM (peptide-spectrum match) values of the corresponding run (Table S9). To investigate if global acetylation levels were enriched in the *Tc*HDAC4 knocked out parasites, we initially analyzed the distribution of the average areas for each PTM (Fig. 5C). However, since all modifications presented a similar fluctuation, it was not possible to detect any significant difference between the samples. As we did not identify a global acetylation defect in knocked out cells, we have calculated the ratio between the area value of each acetylated peptide of knockout relative to wild type cells to look for specific sites (Table S10). Considering that the acetylation levels are not expected to decrease in *Tc*HDAC4 knockout cells, we looked for acetylated peptides that met the following criteria: (1) at least twofold enrichment in the comparison of knockout with wild type metacyclic trypomastigotes and, (2) a value ≥ 1 for the ratio in the comparison between the knockout out metacyclic trypomastigotes and knockout epimastigotes, which is intended to include only the sites in which acetylation is not decreased in metacyclic trypomastigotes and, (3) the parameters of the two criteria above must apply simultaneously to both datasets of the metacyclic stage (Fig. 5D). By applying these criteria, we have identified 4 acetylation sites that met condition (1) (H2AT85ac, H4K4ac H4K10ac and H4T78ac), 7 acetylation sites that met condition (2) (H2AT85ac, H4K10ac, H4T78ac, H2BT24ac, H2AZK54ac, H2AZK58ac and H1K19ac) and three acetylation sites that fulfilled condition (3)—H2AT85ac, H4K10ac and H4T78ac—which are the ones with higher potential to represent specific targets of *Tc*HDAC4 (Table S10). The intensity values of the modified peptides containing H2AT85ac, H4K10ac and H4T78ac in each stage analyzed is shown in Fig. 5E.

## Discussion

In the present work, we showed that the *Tc*HDAC4 is expressed throughout the *T. cruzi* life cycle, although at higher levels in the developmental stages occurring in the invertebrate vector. Characterization of *Tc*HDA4 null mutants indicated that knockout of *Tc*HDAC4 does not have an impact on the replicative stage. Therefore, it is unlikely that *Tc*HDAC4 has a role in DNA replication or cell division as previously proposed for *T. brucei* DAC4^18^. Metacyclic trypomastigotes, however, were deeply affected by the lack of *Tc*HDAC4, showing nuclei with a rounded shape and, presenting uncondensed chromatin and a central nucleolus, which is similar to the nucleus of the epimastigote stage^27^. Moreover, as the nucleolus of *T. cruzi* is structured only in developmental stages in which the parasite proliferates^27^, the presence of a well-defined nucleolar structure in metacyclic trypomastigotes corroborates the hypothesis of an increased demand in the rates of RNA transcription and translation in null mutants parasites.

Consistently, metacyclic trypomastigote from *Tc*HDAC4 null mutants presented several changes in the gene expression profile, showing 673 differentially expressed genes where almost 70% of them were found to be up regulated when compared with wild type parasites. Remarkably, all the 11 ribosomal protein genes (40S ribosomal protein S10, S13, S14, S16, S23, 50S ribosomal protein L16, 60S ribosomal protein L4, L12, L26, L34 and ribosomal protein S7) found differentially expressed were upregulated in metacyclic trypomastigote null mutants (Table S4), suggesting an increase also in translation rates and reinforcing the hypothesis that they are more metabolically active.

**Figure 6:**
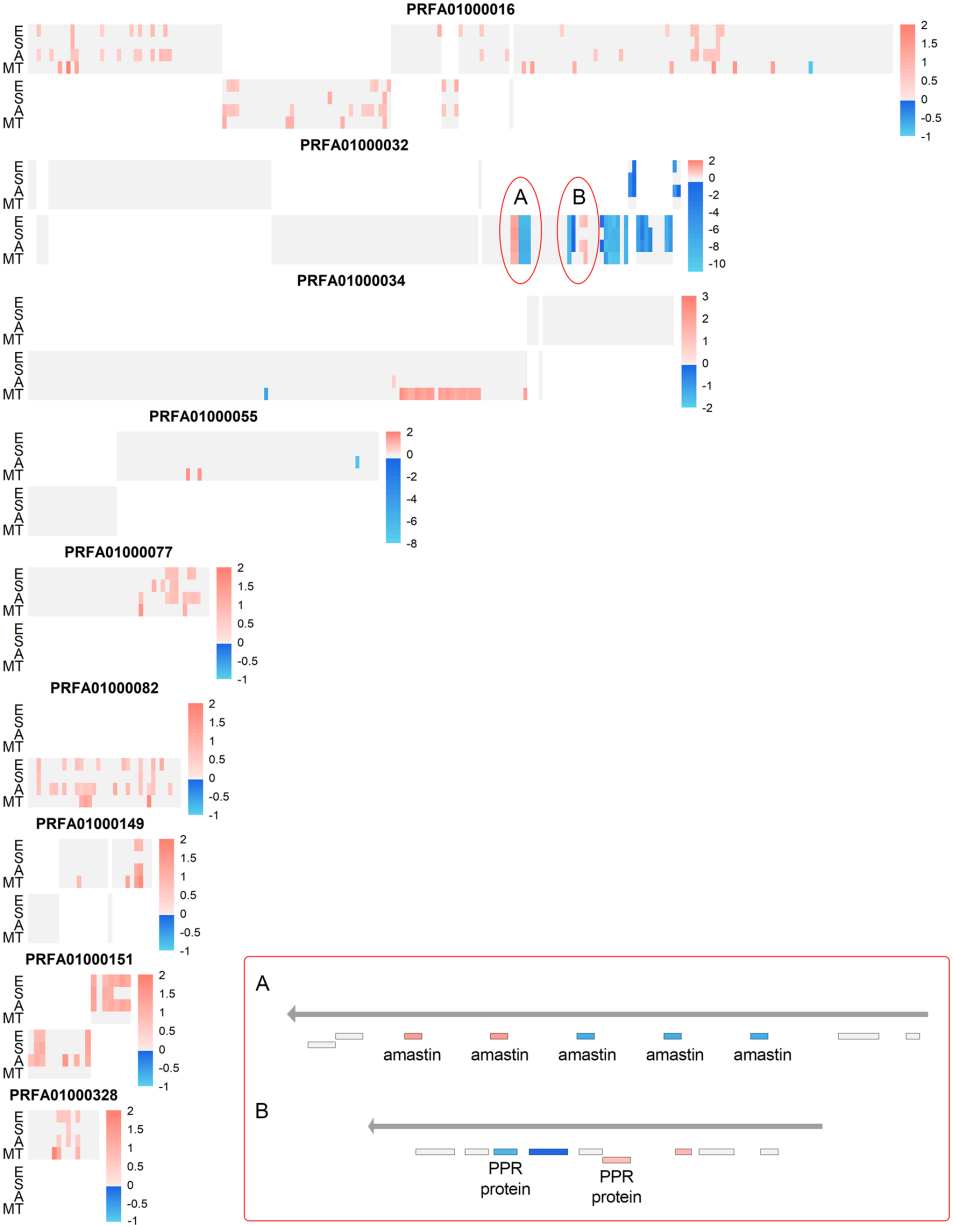
Clusters of differentially expressed genes. Each heatmap shows a chromosome whose ID name is at the top, and it is grouped in 2 panels with the upper panel representing genes encoded in the plus strand and the bottom panel genes encoded in the minus strand. Each column shows a gene, and each line shows a developmental stage comparison (KO/WT), identified by the letters: E, epimastigotes; S, stressed epimastigotes; A, 24 h adhered differentiating forms; MT, metacyclic trypomastigotes. Differentially expressed genes are represented by red and blue colors (z-score scale at left), which indicates up and down regulation (KO/WT), respectively. Genes that were not differentially expressed are represented in grey and absence of genes is represented in white. (**A**) Gene cluster detail including amastin genes. (**B**) Gene cluster details including PPR protein genes.

Indeed, an increase in expression levels was observed for several gene arrays when *Tc*HDAC4 was absent. The clustering observed along the chromosomes suggests that, in most cases, there is a co-regulation of genes from the same polycistronic transcription unit (PTU). It is worth remembering that these regulatory regions—marked by specific chromatin modifications—are enriched at the start of PTUs at divergent strand-switch regions and at other sites of RNA polymerase II transcription re-initiation as between different PTUs transcribed in the same direction^28^ and could explain some examples of neighboring genes being regulated in the opposite way (Fig. 6).

Although further studies are needed to understand the exact mechanism that specifically regulates different copies of the same gene, we believe that some situations deserve to be highlighted, such as that observed for amastins and pentatricopeptide repeat-containing proteins (PPR). Amastins are surface glycoproteins encoded by a diverse gene family. In our data, there is a differentially regulated cluster containing five amastin genes, where two showed about twofold increased whereas the other three were repressed about 200-fold in KO parasites compared with WT (Fig. 6—insert A). This result is noteworthy since the observed repression in some amastin genes could be related to a low rate of *T. cruzi* infection, as suggested elsewhere^29^. The PPR protein family are RNA-binding proteins that play essential roles in post-transcriptional processing, serving as adapters for RNA-editing enzymes in organelles^30^. In our data, one of the copies of the PPR gene showed a slight 1.5-fold increase in its expression while the other copy had a repression of more than 300-fold in KO parasites compared with WT (Fig. 6—insert B). Although quite interesting, we cannot affirm that in both situations the observed differences are directly caused by local chromatin modifications, or otherwise by changes in mRNA stability due to the absence of *Tc*HDAC4.

In a more detailed analysis, it was possible to observe a differential regulation of genes encoding some surface proteins. The large multigene families coding for surface proteins are clustered in arrays distributed on virtually all chromosomes. These clusters can include genes for mucin, mucin-associated surface proteins (MASP), gp85/TS-like, GP-63, serine-alanine-proline rich protein (SAP), dispersed gene family 1 (DGF-1) and trans-sialidase families^31^. Several of these genes encoding surface proteins that are involved with the infection process were found differentially expressed in the *Tc*HDAC4 null mutant parasites (Tables S1–S4). Mucins are the major *T. cruzi* surface glycoproteins in epimastigotes and trypomastigotes^32^, which are crucial to ensure invasion of cells and to protect the parasite against host defensive mechanisms^33,34^. Mucin-associated surface proteins (MASP) are also involved in host-parasite interactions^35^. Notably, some of these genes have a different expression profile when *Tc*HDAC4 is depleted: one mucin (*Tc*MUCII) gene and four MASP genes were down-regulated in epimastigotes and 21 mucins (*Tc*SMUGS) genes were up-regulated in metacyclic trypomastigotes (Tables S1 and S4). It is worth mentioning that out of the 21 differentially expressed *Tc*SMUGS, 20 are located in the same cluster at chromosome PRFA01000034. Interestingly, the genes that encode surface proteins present in a specific cluster were found strongly repressed (about 100-fold) in the null mutant at all stages of development analyzed, except in the metacyclic trypomastigote stage (Tables S1–S4).

Trans-sialidases and surface membrane protein family members are mediators of host-parasite interactions, being important also to the initial stages of *T. cruzi* invasion to the host cells^36^. Some of the genes encoding them, described previously as up-regulated in metacyclic trypomastigote forms of wild type parasites^26^, were found down-regulated in the present work (Table S4) and could be related with lower infection rates observed for null mutant parasites.

Arrays of several genes—sometimes more than 100 genes—are transcribed in polycistronic transcription units that are characterized by distinct chromatin modifications at its beginning and end^37^. Histone acetylation is almost invariably associated with activation of transcription and previous studies showed an enrichment of acetylated histone H3 and H4 at Pol II transcriptional start sites in trypanosomatids^38^. Alterations in the histone mark profiles by deletion of histone modifying enzymes can profoundly impact the gene expression programs. We have identified 34 novel histone acetylation sites and quantitatively compared 64 acetylation sites in this work. Although no increase in the global levels of acetylation were observed when comparing mutant and wild type cells, this work significantly expanded the number of histone PTM sites identified for *T. cruzi*, and identified three enriched sites—H4K10ac, H2AT85ac and H4T78ac—which probably correspond to targets of *Tc*HDAC4.

Acetylation of the N-terminal tail of histone H4 weakens the interactions with DNA, which results in a destabilization of nucleosomal structures, that disrupt the high-order folding of chromatin fibers. This contributes to an open chromatin conformation favoring active transcription directly or by providing binding sites for activators^39^. Although trypanosome histone tails are divergent, some modifications appear to occur in conserved positions relative to other eukaryotes. It has already been shown that residues K4, K10 and K14 of histone H4, homologous to K5, K12 and K16 of H4 from other organisms, could be acetylated also in *T. cruzi* and *T. brucei*^7,40^. H4K12ac has been described as a characteristic of transcribing cells and is enriched surrounding transcriptional start sites (TSSs). As for other organisms, H4K10 was found enriched in *T. brucei* TSSs and was also associated with transcription initiation^41^. In *T. cruzi,* mutation of K10 to R10 in histone H4 prevented H4R10 acetylation and caused transcription impairment^42^. Here, the knockout of *Tc*HDAC4 caused a maintenance of high acetylation levels at H4K10. The associated increase in gene expression levels in metacyclic trypomastigote *Tc*HDAC4-knockout parasites corroborates that H4K10ac could be one of the targets of *Tc*HDAC4. We cannot exclude that *Tc*HDAC4 knockout and the resulting change in the gene expression profile has also affected RNA processing and RNA stability, besides increasing histone acetylation and access to the gene transcription start sites. Future experiments, involving direct RNA transcription rate and RNA decay assays could address this question.

Acetylation and deacetylation of serine and threonine residues, however, is still a little explored field. One of the best known examples is the bacterial family of YopJ effectors: the plague bacteria *Yersinia pestis* uses acetyltransferases to acetylate phosphosites in proteins of the MAPK pathway, thereby preventing signal transduction in the host cell and impairing the immune response^43^. Although our data suggest that acetylation of H2AT85 and H4T78 may be targets of *Tc*HDAC4, in-depth characterization of enzyme recruitment and specificity will be necessary to demonstrate this hypothesis.

Based on our results, we suggest that the changes occurring during metacyclogenesis, such as nuclear structure alterations, mitochondrial DNA rearrangements, chromatin remodeling and the resulting decrease in gene expression levels of a set of genes, most probably represent the consequences of the differentiation process and are not the events that trigger this process. Our findings corroborate the hypothesis that access to transcription start sites and the resulting transcriptional activity in *T. cruzi* is mediated also by acetylation of nucleosomal histones. Access to transcription start sites is a key point in controlling the level of gene expression and is especially important for parasites that need to rapidly adapt to different hosts during their life cycle. In conclusion, we suggest that the biological function of *Tc*HDAC4 is to provide reversible means of switching RNA synthesis off at different chromosomal loci during *T. cruzi* metacyclogenesis.

## Methods

### Ethics and compliance statements

All methods used in this study were carried out in accordance with the relevant guidelines and regulations. Animal experiments were approved by the Ethical Committee on Animal Experimentation of the Oswaldo Cruz Foundation (CEUA / protocol number: P47/12-3, license number: LW-15/13). The study was carried out in compliance with the ARRIVE guidelines (https://arriveguidelines.org/). Experiments using genetically modified organisms classified as biosafety level 1 and 2 were approved by the Brazilian National Committee of Biosafety (CTNBio) under the permission number CQB: 313/10.

### HDAC sequences and multiple sequence alignments.

The amino acid sequence of the histone deacetylases 1–4 from *T. cruzi* strain Dm28c 2014^44^ were obtained from the TriTrypDB database. Their respective identification numbers are: C4B63_79g84, C4B63_9g487, C4B63_34g373, C4B63_31g166. The identification numbers of HDAC4 from *Trypanosoma brucei* and *Leishmania major* in the TriTrypDB database are Tb427.05.2900 and LmjF.08.1090, respectively. The closest human homologs of *Tc*HDAC1 and *Tc*HDAC4 were identified using PSI-BLAST (https://blast.ncbi.nlm.nih.gov/Blast.cgi). They correspond to human HDAC1 and HDAC6, and their sequences were retrieved from the GenBank using the accession numbers, NP_004955.2 and AAH69243.1. The histone deacetylase domains of *T. cruzi* HDAC1-4 were determined using the Pfam database (https://pfam.xfam.org). Multiple sequence alignments were performed with Clustal Omega (https://www.ebi.ac.uk/Tools/msa/clustalo/).

### *Trypanosoma cruzi* cultures and proliferation assays

*T. cruzi* Dm28c^45^ epimastigotes were cultured in liver infusion tryptose (LIT) medium^46^ supplemented with 10% fetal bovine serum (FBS) and, when necessary the required antibiotic, without agitation at 28 °C. Cell proliferation assays were started with 1 × 10^6^ epimastigote cells ml^-1^ and the population growth was monitored during four days by cell counting using a hemocytometer every 24 h. The experiments included technical and biological replicates. Data were subjected to statistical analysis by unpaired t-test using GraphPad Prism v8 software.

### Targeted gene deletion

Targeted gene deletion was achieved by homologous recombination. For this purpose, the flanking sequences of the *TcHDAC4* gene were initially amplified by PCR from *T. cruzi* Dm28c genomic DNA using the primers DAC4UpsF + R and DAC4DownF + R (Table S11, HDAC4 5’ and 3’ flanks). The PCR fragments were inserted into the vectors pTc2KO-neo and pTc2KO-hyg^47^, carrying neomycin phosphotransferase and hygromycin phosphotransferase B gene cassettes, respectively, using the *Kpn*I and *Sal*I for the 5’ flank and the *Bam*HI and *Xba*I restriction sites for the 3’ flank. Subsequently, the complete knockout cassettes pTc2KO-*Tc*HDAC4-neo and pTc2KO-*Tc*HDAC4-hygro were PCR-amplified using the primers DAC4UpsF + DAC4DownR (Table S11) and purified using the QIAquick PCR purification kit (Qiagen). *T. cruzi* Dm28c epimastigote cells were transfected with 20 µg of each cassette by electroporation. Briefly, parasites were transfected with the 5′flank-hygro-3′ flank cassette and the transfectants selected by incubation in LIT medium containing 500 µg ml^−1^ hygromicin B. Subsequently, the hygromicin B-resistant population was transfected with the 5′flank-neo-3′flank cassette and selected in LIT medium containing 500 µg ml^−1^ G418 and 500 µg ml^−1^ hygromicin B. Absence of *Tc*HDAC4 expression in the double resistant parasite population was checked by immunoblotting using the *Tc*HDAC4 antiserum obtained as described below.

*Tc*HDAC4 null mutant clones were obtained by cell sorting of the double resistant population using a BD FACSAria II (Becton–Dickinson). Correct integration of the resistance markers in the *TcHDAC4* locus was verified by PCR amplification from genomic DNA using the primers DAC4ExtF and DAC4ExtR that anneal in regions upstream and downstream of the inserted cassettes (Table S11, HDAC4 locus). The presence/absence of *TcHDAC4* gene was also determined by PCR using the DAC4UpsF + DAC4R primer combination. The PCR amplification products were analyzed in agarose gels stained with 0.5 µg ml^−1^ ethidium bromide.

### DNA extraction

For DNA extraction, 1 × 10^8^ cells were pelleted, washed twice with phosphate-buffered saline, pH 7.4 (PBS) and, incubated with 150 µl of TELT lysis buffer (50 mM Tris–HCl pH 8.0, 62.5 mM EDTA pH 8.0, 2.5 M LiCl and 4% Triton X-100) for 5 min at room temperature. Then, the DNA was purified using phenol:chloroform:isoamyl alcohol (25:24:1, v/v), precipitated with 100% ethanol (2.5:1, v/v) and resuspended in 100 µl of TE containing 10 µg ml^-1^ RNase A.

### Metacyclogenesis and *T. cruzi* differentiation in vitro

Differentiation of epimastigotes into metacyclic trypomastigotes was accomplished in vitro using chemically defined culture medium^48^. Briefly, epimastigotes in late exponential growth phase (~ 5 × 10^7^ cells ml^-1^) in LIT medium were harvested by centrifugation, resuspended in triatomine artificial urine (TAU) medium (190 mM NaCl; 17 mM KCl: 2 mM MgCl:; 2 mM CaCI2; 8 mM phosphate buffer, pH 6.0; 0.035% w/v sodium bicarbonate) at a density of 5 × 10^8^ cells ml^-1^ and incubated for 2 h at 28 °C to obtain stressed epimastigotes which were transferred to culture flasks containing TAU3AAG medium (TAU medium supplemented with 50 mM sodium L-glutamate, 2 mM sodium L-aspartate, and 10 mM glucose) at a final density of 5 × 10^6^ cells ml^−1^ and incubated at 28 °C for 72 h. After this time, the metacyclic trypomastigotes were purified by DEAE cellulose chromatography (D-3764, Sigma), as previously described^49^. Cell differentiation efficiency was determined by counting the different cell types from the supernatant of the 72 h-in vitro metacyclogenesis assay using a hemocytometer. These assays were performed including biological and technical replicates and the data subjected to statistical analysis by unpaired t-test using GraphPad Prism v8 software. Samples from the intermediate forms (stressed epimastigotes and 24 h adhered differentiating forms) and the 72 h differentiated metacyclic trypomastigotes were collected during in vitro metacyclogenesis for subsequent analysis.

Amastigotes were obtained by in vitro amastigogenesis as described elsewhere^50^. Briefly, cell-derived trypomastigotes were cultivated in acidic high glucose DMEM (pH 5.0) at 37 °C for 24 h and the amastigotes collected by centrifugation. Epimastigogenesis to obtain epimastigotes from cell-derived trypomastigotes was performed following a procedure previously described elsewhere^51^ with minor modifications. Briefly, cell-derived trypomastigotes were cultivated in LIT (without FBS) at 28 °C for 3 days, then recently differentiated epimastigotes were maintained in culture in LIT supplemented with 10% FBS at 28 °C.

### Production of an antiserum against *Tc*HDAC4

The coding sequence of the *TcHDAC4* gene was amplified from *T. cruzi* Dm28c genomic DNA using the specific primers HDAC4_F and HDAC4_R (Table S11, HDAC4 coding sequences), inserted into the Gateway entry vector pDONR221 (Invitrogen), and subsequently transferred to the expression vector pDEST17 (Invitrogen), where it was cloned in fusion with an N-terminal hexa-histidine tag. The His6x-*Tc*HDAC4 recombinant protein was produced in *Escherichia coli* BL21 Star (DE3) (Thermo Scientific) after induction with 1 mM IPTG, solubilized from inclusion bodies with 8 M urea and purified using Ni–NTA columns (Qiagen). Swiss mice were immunized with four injections of 20 µg of the purified His6x-*Tc*HDAC4 protein in Alu-Gel-S adjuvant (Serva) at 14-day intervals. The antiserum specificity was determined by western blot assays using both purified His6x-*Tc*HDAC4 protein and *T. cruzi* epimastigote cell lysates. Animal experiments were approved by the Ethical Committee on Animal Experimentation of the Fundação Oswaldo Cruz (CEUA / protocol number: P47/12-3, license number: LW-15/13).

### Immunoblotting

Epimastigotes and metacyclic trypomastigotes were obtained as described above. Cell-derived trypomastigotes were obtained from infected Vero cells (ATCC CCL-81). Briefly, Vero cells were infected with metacyclic trypomastigotes at a 1:100 host cell/parasite ratio. After 96 h of culture, the cell-derived trypomastigotes were collected from culture supernatants by centrifugation^52^. For analysis by western blot, whole protein extract from approximately 10^7^ cells was loaded in each gel lane. Following separation by electrophoresis, the proteins were transferred to nitrocellulose membranes, which were incubated with antisera raised against *Tc*HDAC4 and *Tc*GAPDH and the blots developed following standard protocols^53^. A polyclonal mouse antibody against *T. cruzi* glyceraldehyde 3-phosphate dehydrogenase (GAPDH) was kindly provided by F Morini [Carlos Chagas Institute, Oswaldo Cruz Foundation (Fiocruz), state of Paraná, Brazil].

### Morphological characterization

Panoptic staining was performed as previously described^54^ using a total of 1–10 × 10^6^ epimastigotes or metacyclic trypomastigotes. Briefly, cells were collected by centrifugation, washed in PBS, deposited on glass slides, allowed to dry and fixed in cold methanol. After drying, the cells were clarified for 4 min with 5 M HCl, washed 5 times with water and dried again. Subsequently, the dried cells were stained with Panoptic (Laborclin) according to the manufacturer’s instructions with minor modifications: 2 min in solution 1, 15 min in solution 2 and 15 min in solution 3. Finally, the cells were analyzed by light microscopy. Images were acquired using the Nikon Eclipse E600 or Leica DMi8 microscope.

Scanning and transmission electron microscopy assays were performed essentially as described previously^55^. For scanning electron microscopy, the cells were washed twice in PBS and fixed for 1 h at room temperature in 2.5% glutaraldehyde in 0.1 M phosphate buffer, pH 7.2. The fixed parasites were washed twice in 0.1 M cacodylate buffer and adhered for 15 min at room temperature to 0.1% poly-L-lysine coated coverslips. The non-adhered cells were washed out twice with 0.1 M cacodylate buffer and the adhered cells were incubated for 15 min at room temperature with 1% osmium tetroxide diluted in 0.1 M cacodylate buffer. The samples were washed three times with 0.1 M cacodylate buffer and dehydrated in a graded acetone series (30%, 50%, 70%, 90% and 100% acetone, 5 min each)^55^. These steps were followed by critical point drying and gold sputtering using a Leica EM CPD300 and a Leica EM ACE200 system, respectively. The scanning electron microscopy images were acquired on a JEOL JSM-6010 PLUS/LA scanning electron microscope at 20 kV. For transmission electron microscopy, the cells were washed twice in PBS and fixed for 1 h in 2.5% glutaraldehyde in 0.1 M phosphate buffer at room temperature. The fixed cells were washed twice in 0.1 M cacodylate buffer and post-fixed with 1% osmium tetroxide / 1.6% potassium ferrocyanide / 5 mM CaCl_2_ diluted in 0.1 M cacodylate buffer for 30 min at room temperature. The samples were washed three times with 0.1 M cacodylate buffer, dehydrated in a graded acetone series (5 min in 30%, 50%, 70%, 90% and 100%) and embedded in PolyBed812 resin. Ultrathin sections were obtained using a Leica EM UC6 ultramicrotome, collected on copper grids and contrasted with uranyl acetate and lead citrate^55^. The transmission electron microscopy images were obtained on a JEOL 1400Plus transmission electron microscope at 90 kV. Brightness and contrast were adjusted using the Adobe Photoshop C5 software.

### Vero cell infection assays

Vero cells (ATCC CCL-81) were cultured in low glucose DMEM (Gibco) supplemented with 10% FBS, 10 U ml^−1^ of penicillin and 10 µg ml^−1^ of streptomycin at 37 °C in a humid 5% CO_2_ environment. Metacyclic trypomastigotes from wild type and *Tc*HDAC4 null mutant cells were obtained as described above. Cellular trypomastigotes were obtained after three passages in Vero cells. For the infection assays, 3 × 10^3^ Vero cells previously adhered to 96-well plates for 4 h were infected with a MOI of 100:1 of metacyclic or a MOI of 20:1 of cellular trypomastigotes for 24 or 16 h, respectively. After this time, the medium containing free trypomastigotes that had not infected cells was replaced by fresh medium and the cultures were maintained for further 72 h under the same conditions. For cell counting, the wells were washed twice with PBS prior and after fixation with 4% paraformaldehyde (PFA) for 15 min and stained with 0,001% Evans’ blue and 1 μg ml^-1^ DAPI. Images of > 20 fields per well were acquired on an Operetta Image System (PerkinElmer) and analyzed using the Harmony software (PerkinElmer) to discriminate nucleus, cytoplasm and the spots delimiting the intracellular amastigotes. The number of infected and non-infected cells was determined, plotted using the GraphPad Prism v.8 software and statistically analyzed by the Mann–Whitney test.

### RNA-Seq assays

Differentiation of epimastigotes to metacyclic trypomastigotes of wild type and null mutant parasites was accomplished as described above. Total RNA was extracted from 1 × 10^8^ epimastigotes, stressed epimastigotes, 24 h adhered differentiating forms and metacyclic trypomastigotes using the RNeasy kit (Qiagen) following the “Animal Cells I” protocol from the manufacturer’s instructions. RNA samples were quantified using the Qubit RNA HS Assay (Thermo Fisher Scientific), purity was evaluated using the 260/280 nm absorbance ratio on NanoDrop ND-1000 Spectrophotometer (NanoDrop Technologies) and integrity verified using the Agilent RNA 6000 Pico Kit (Agilent Technologies) on a 2100 Bioanalyzer Instrument (Agilent Technologies). Stranded tagged cDNA libraries were prepared using the KAPA Stranded mRNA-Seq Kit (Illumina) and cluster generation was performed using the Illumina HiSeq PE Cluster Kit v4 cBot. Tagged RNA-Seq libraries were pooled and sequenced (300 cycles, paired-end sequencing) on an Illumina HiSeq 2500 instrument using a HiSeq SBS Kit V4. Raw reads were preprocessed using the standard Illumina pipeline to segregate multiplexed reads.

The RNA-Seq datasets were inspected with FASTQC (v0.11.7)^56^⁠, which revealed that reads from all samples showed high quality control parameters. Next, the FASTP (0.20.0)^57^⁠ algorithm was used to remove adapter sequences and short fragments (< 15 nt). To determine the best genome version that suited our organism of interest, we used QUAST (v.5.0.2)^58^, and selected the genome that showed the best parameters according to the number of contigs, N50, larger contigs and genome coverage. Five genome assembly versions, namely Dm28c 2014, Dm28c 2017, Dm28c 2018, SylvioX10-1-2012 and SylvioX10-1 that are available at TriTrypDB (release 44)^59^ were evaluated. To help evaluate the best genome assembly, we also mapped our RNA-Seq reads to the five versions of the genome using Bowtie (v. 2.2.9)^60^, with default and very sensitive parameters (Supplementary Methods). This led to the selection of the Dm28c 2018 genome. Subsequently, RNA-Seq reads alignment and counting were performed using Kallisto (v 2.7.0c)^61^⁠ with default parameters, using the Dm28c 2018 transcriptome comprising 19,122 genes (TriTrypDB – release 44) as reference. This algorithm was selected because it works with pseudoalignments and expectation–maximization approaches, which tries to infer the probability of the read to be originated from a specific transcript. This approach is valuable to deal with multi-mapping reads and can suit to transcriptomes that are poorly annotated, because pseudoalignments can increase the probability of identifying the transcript of origin without mapping the entire read within it. Statistical analyses were performed with three different algorithms to compute differential gene expression: Sleuth (v 0.30.0)^62^, voom with weights (v 3.42.2)^63^ and EdgeR (v 3.28.1)^64,65^ with svaseq (v 3.34.0)^66^. False Discovery Rate (FDR) was used to correct for multiple testing and only genes that presented FDR ≤ 0.05 were selected. Only differentially expressed genes that overlapped in all three statistical approaches were used for further analyses. For assays without replicate, we applied the NOISeq (v 2.28.0)^67^ algorithm with overall variability 0.02, which was calculated among all samples using the biological coefficient variation (BCV) function from EdgeR. The subsample parameter was 20% of all reads with 5 replicates. These parameters were chosen according to the coefficient of variation of differentially expressed genes (Supplementary Methods). The DEGs were shown with unsupervised heatmap clustering (Figures S4A, S9 and SM1) that were built using the pheatmap (v 1.0.12) function in the R platform (v 3.6.2)^68^. The heatmaps on chromosomes (Figs. 3C, 6) were generated with the same software by disabling the clustering function option.

### Sample preparation for the analysis of histone post-translational modifications (PTMs)

Differentiation of epimastigotes to metacyclic trypomastigotes of wild type and *Tc*HDAC4 null mutant cells was accomplished as described above. Histone-enriched extracts were obtained in quadruplicates as previously described^11^ with some modifications. Briefly, 2 × 10^8^ epimastigotes and DEAE-purified metacyclic trypomastigotes were collected by centrifugation, lysed in 1 mL of Buffer A (0.25 M Sucrose; 1 mM EDTA; 3 mM CaCl_2_; 0.01 M Tris–HCl pH 7.4; 0.5% (v/v) Saponin; protease inhibitor cocktail tablets (Roche)). After centrifugation, the pellet containing cell debris and nuclei was washed once in 1 mL of Buffer B (0.25 M Sucrose; 1 mM EDTA; 3 mM CaCl_2_; 0.01 M Tris–HCl pH 7.4; protease inhibitor cocktail tablets (Roche)), once in 1 mL of Buffer C (1% (v/v) Triton X-100; 0.15 M NaCl; 0.025 M EDTA; 0.01 M Tris–HCl pH 8; protease inhibitor cocktail (Roche)) to remove the soluble nuclear proteins and washed 3 times in 100 mM Tris–HCl pH 8. After incubation with 1 mL of 0.4 N HCl, the acid-soluble proteins were recovered in the supernatant and precipitated in 8 volumes of acetone at –20 °C overnight. Next, the samples were centrifuged, washed 3 times with acetone, dried at 37 °C and resuspended in 50 μL of water.

### Sample preparation for LC–MS/MS

Initially, the protein samples were subjected to propionylation at lysine residues^69^. For this, 20–40 µg of protein were diluted in 15 µL of 100 mM ammonium bicarbonate (ABC) pH 8.0, and the volume was adjusted to 30 µl with ultrapure water. The propionylation reagent was made by adding one volume of propionic anhydride to 3 volumes of 2-propanol, followed by brief vortexing. 10 µL of the propionylation reagent were added to the samples. For each reaction, 6 µL of 100 mM ABC was immediately added to the histone sample, to balance the pH. A pH indicator strip was used to monitor the pH at this time, to make sure that the pH was in a range from 7 to 9. The samples were then incubated in a thermomixer at 37 °C for 15 min, dried in a vacuum centrifuge to obtain 5–10 µL and diluted with ultrapure water to obtain a volume of 30 µL. The process was repeated once to increase the propionylation efficiency. After the second propionylation round, the volume was adjusted to 60 µL with 15 µL of 100 mM ABC and 15 µL of ultrapure water. For protein digestion, trypsin was added in a 1:20 enzyme to protein ratio and incubated at 37 °C overnight. The digestion was stopped with 3 µL of glacial acetic acid. The peptides were desalted on C18 homemade StageTips before LC–MS/MS analysis.

### Mass spectrometry data acquisition

For mass spectrometry analysis, 0.2 µg (epimastigote) or 0.5 µg (metacyclic trypomastigote) of desalted peptides were separated in a packed emitter column with 15 cm length, 75 µm I.D., and 3 µm C18 particles (Dr. Maisch). Injection and separation were assisted by a Eksigent system composed of a nanoLC 1d plus and an as-2 autosampler. Separation was carried out during 120 min using as mobile phases 0.1% acid formic, 5% DMSO in LC–MS water (phase A) and 0.1% formic acid, 5% DMSO in acetonitrile (phase B). The gradient was set from 4 to 40% phase B at a flow rate of 250 nL/min. The LC system was coupled to a Thermo Scientific LTQ Orbitrap XL, equipped with a nano electrospray source (Phoenix S&T). MS acquisition parameters were: source spray voltage of 2.7 kV, spray current of 100 μA and capillary heater 175 °C; MS1 was done in the Orbitrap analyzer with a resolution of 60,000, m/z window of 300 to 1800, and the preview scan enabled; MS2 analysis was performed in a DDA mode, where the ten most intense ions were subjected to CID fragmentation in the ion trap analyzer; the MS1 lock mass was set to 401.922718 m/z. The runs were done in technical triplicate (epimastigotes) or duplicate (metacyclic trypomastigotes). Two rounds of mass spectrometry data were acquired for each sample. This resulted in 32 datasets, which were initially inspected for similar NL (normalization level) values of peak intensity. The datasets with lower NL values were excluded to avoid bias in subsequent comparisons. The analysis was performed with eight datasets each of the TcHDAC4 knockout and control cells for the epimastigote stage and with two datasets each for the metacyclic trypomastigote stage.

### Mass spectrometry data analysis

Data analysis was performed using Peaks DB, PTM and SPIDER tools from Peaks Studio [version 10.0, Bioinformatics Solutions Inc]. Proteins were searched against a *T. cruzi* histone database, which consists of two proteome sets of histone sequences and against a database of frequently observed contaminants. The proteins matching the contaminants database were excluded from the analysis. The precursor mass tolerance was set to 10 ppm and the fragment ion mass tolerance was set to 0.5 Da. The searches required enzymatic specificity and a minimum of five amino acids for peptide length, allowing for four missed cleavages. The search parameters for PTMs were set as variable modifications: Acetylation [K] (42.01); Phosphorylation [STY] (79.97); 2-Hydroxyisobutyrylation (86.04); Succinylation (100.02); Formylation (27.99); Ubiquitination (383.23); Citrullination (0.98); Hydroxylation (15.99); Methylation[Protein N-term] (14.02); Propionyl (56.03); MethylPropionyl (70.04) ; Acetylation [TSCYH] (42.01); Acetylation [N-term] (42.01); Acetylation [Protein N-term] (42.01); Methylation[KR] (14.02); Dimethylation[KR] (28.03); Trimethylation (42.05). All modifications and mutations in the Unimod database were searched as well. For all searches, the false discovery rate [FDR] was below 1.1%, as estimated by decoy-fusion method. PTM sites with Ascore ≥ 20 were automatically validated. For the quantitative analysis, a single representative sequence chosen by the highest coverage index was used for each *T. cruzi* histone (**H2A**: BCY84_04071, **H2B**: BCY84_11072, **H3**: BCY84_02630, **H4**: C4B63_188, **H1**: BCY84_14675, **H2AZ**: BCY84_22061, **H2BV**: C4B63_30 and **H3V**: C4B63_4). The chromatographic peak area of each modified peptide feature was normalized to the PSM (peptide-spectrum match) values of the corresponding run (Table S9). Global PTMs and acetylation levels were first analyzed by comparing the distribution of the average areas for each PTM between the wild type and *Tc*HDAC4 knockout cells. To investigate whether global acetylation or specific acetylation sites were affected *Tc*HDAC4 knockout, we have calculated the ratio between the area value of each acetylated peptide of *Tc*HDAC4 knockout relative to wild type cells (Table S10). Considering that the acetylation levels are not expected to decrease in *Tc*HDAC4 knockout cells, we looked for acetylated peptides that met the following criteria: (1) at least 2 × enrichment in the comparison of knockout with wild type metacyclic trypomastigotes and, (2) a value ≥ 1 for the ratio in the comparison between the knockout out metacyclic trypomastigotes and knockout epimastigotes, which is intended to include only the sites in which acetylation is not decreased in metacyclic trypomastigotes and, (3) the parameters of the two criteria above must apply simultaneously to both datasets of the metacyclic stage. A flowchart describing the steps to determine the potential acetylation sites modulated by *Tc*HDAC4 in *T. cruzi* metacyclic trypomastigotes was generated using Adobe Photoshop CC version 20.0.4 (https://www.adobe.com/).

## Supplementary Information

Supplementary Information.

## Abbreviations

ABC: Ammonium bicarbonate
Ac: Acetylation
Cit: Citronylation
DAC: Deacetylase
DAPI: 4′: 6-Diamidino-2-phenylindole
DEG: Differentially expressed genes
DGF-1: Dispersed gene family 1
DMSO: Dimethyl sulfoxide
FBS: Fetal bovine serum
FDR: False discovery rate
HDAC: Histone deacetylase
Hib: 2-Hydroxyisobutyrylation
IPTG: Isopropyl β-d-thiogalactoside
KO: Knockout
LIT: Liver infusion tryptose
MASP: Mucin-associated surface proteins
Me: Methylation
Me2: Dimethylation
Me3: Trimethylation
OH: Hydroxylation
PBS: Phosphate-buffered saline
PFA: Paraformaldehyde
Ph: Phosphorylation
PSM: Peptide-spectrum match
PTM: Post-translational modification
PTU: Polycistronic transcription unit
SAP: Serine-alanine-proline rich protein
Su: Succinylation
TAU: Triatomine artificial urine
TSS: Transcriptional start site
Ub: Ubiquitination
VSG: Variable surface glycoprotein
WT: Wild type
