# Supplementary material for: Metacyclogenesis defects and gene expression hallmarks of histone deacetylase 4-deficient Trypanosoma cruzi cells
Source: Sci Rep. 2021 Nov 4;11:21671. doi: 10.1038/s41598-021-01080-1 (PMC8569148; doi:10.1038/s41598-021-01080-1)
Supplement: Supplementary file 1 — Supplementary Information. [file 41598_2021_1080_MOESM1_ESM.pdf]

Supplementary information for the paper:

Metacyclogenesis defects and gene expression hallmarks of histone deacetylase 4-deficient *Trypanosoma cruzi* cells

Gisele Fernanda Assine Picchi-Constante [1, *]; Eloise Pavão Guerra-Slompo [1, *]; Ana Carolina Tahira [2]; Monica Visnieski Alcantara [1]; Murilo Sena Amaral [2]; Arthur Schveitzer Ferreira [1]; Michel Batista [1]; Cassiano Martin Batista [1], Samuel Goldenberg [1]; Sergio Verjovski-Almeida [2, 3]; Nilson Ivo Tonin Zanchin [1, #]

[1] Instituto Carlos Chagas, Fiocruz Paraná, Curitiba, Paraná 81350-010, Brazil.

[2] Laboratório de Parasitologia, Instituto Butantan, 05503-900, São Paulo SP, Brazil.

[3] Departamento de Bioquímica, Instituto de Química, Universidade de São Paulo, 05508-900 São Paulo, SP, Brazil.

* These authors contributed equally to this work

[#] Corresponding author. Email address: nilson.zanchin@fiocruz.br

Supplemental figures

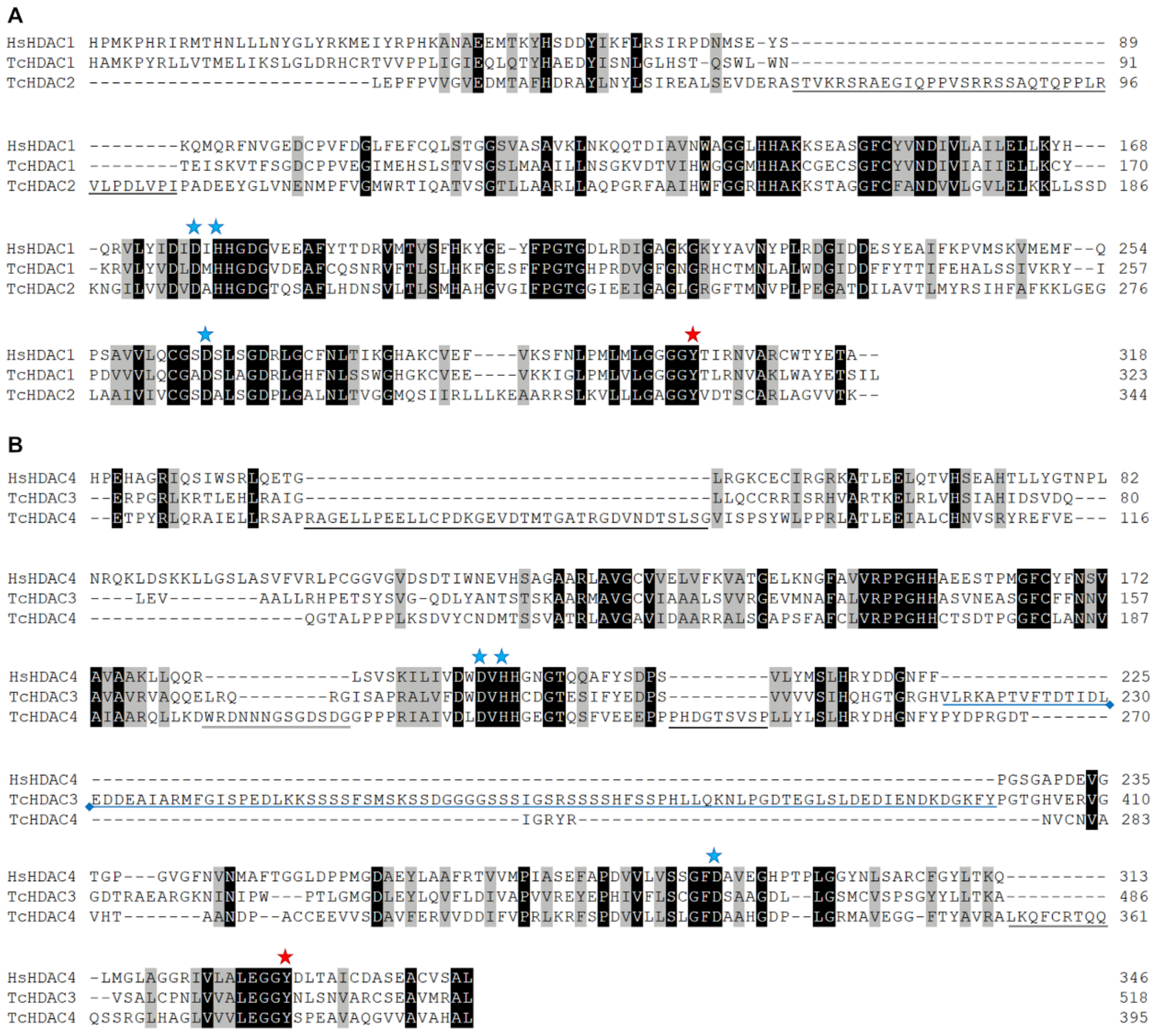

C

Histone deacetylase domain aminoacids identity (%)					
Class I			Class II		
	HsHDAC1	TcHDAC1		HsHDAC4	TcHDAC3
HsHDAC1			HsHDAC4		
TcHDAC1	49		TcHDAC3	39	
TcHDAC2	38	31	TcHDAC4	35	33

Figure S1. Multiple sequence alignment of the histone deacetylase domains (according to Pfam identification) of (**A**) *Trypanosoma cruzi* class I HDACs and human HDAC1 (Hs) and, (**B**) *T. cruzi* class II HDACs and HsHDAC6 (second HDAC domain). Sequences are colored according to amino acid identity (black background) or similarity (gray background). Residues involved in zinc binding are shown with blue stars and the catalytic tyrosine as red star. Insertions in the *T. cruzi* enzymes are underlined. The large insertion in the middle of *Tc*HDAC3 is underlined in blue. The sequence identities are shown in (**C**). The *T. cruzi* HDAC sequences were retrieved from the TriTrypDB database. Their identification numbers: *Tc*HDAC1, C4B63_79g84; *Tc*HDAC2 C4B63_9g487; *Tc*HDAC3, C4B63_34g373. *Tc*HDAC4, C4B63_31g166. The sequence of human DAC1 and HDAC6 were retrieved from the GenBank using the accession numbers, NP_004955.2 and AAH69243.1.

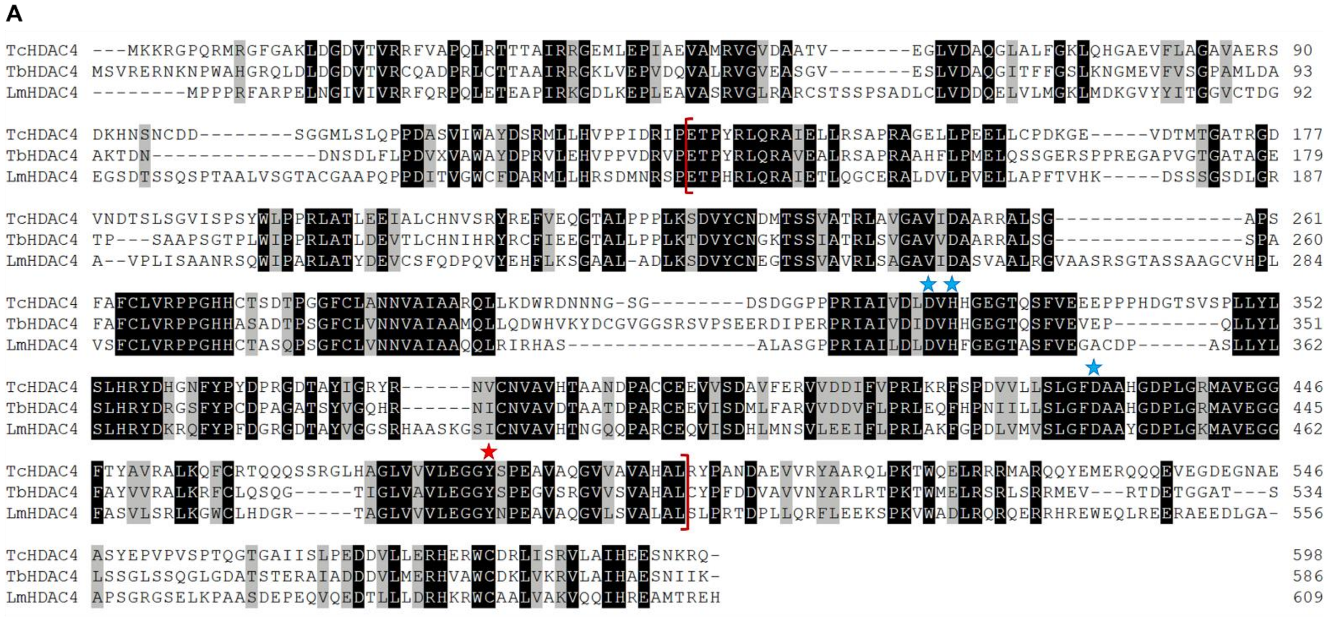

B

Histone deacetylase 4 aminoacids identity (%)		
	TcHDAC4	TbHDAC4
TcHDAC4		
TbHDAC4	58	
LmHDAC4	45	43

Figure S2. Analysis of sequence conservation of trypanosomatid HDAC4 orthologs. (A) Multiple sequence alignment of the histone deacetylases 4 from *T. cruzi* (C4B63_31g166) *T. brucei* (Tb427.05.2900) and *Leishmania major* (LmjF.08.1090) The HDAC domain is indicated between red brackets. Sequences are colored according to amino acid identity (black background) or similarity (gray background). Residues involved in zinc binding are indicated with blue stars and the catalytic tyrosine with a red star. (B) percentage of sequence identity between the trypanosomatid HDAC4 orthologs.

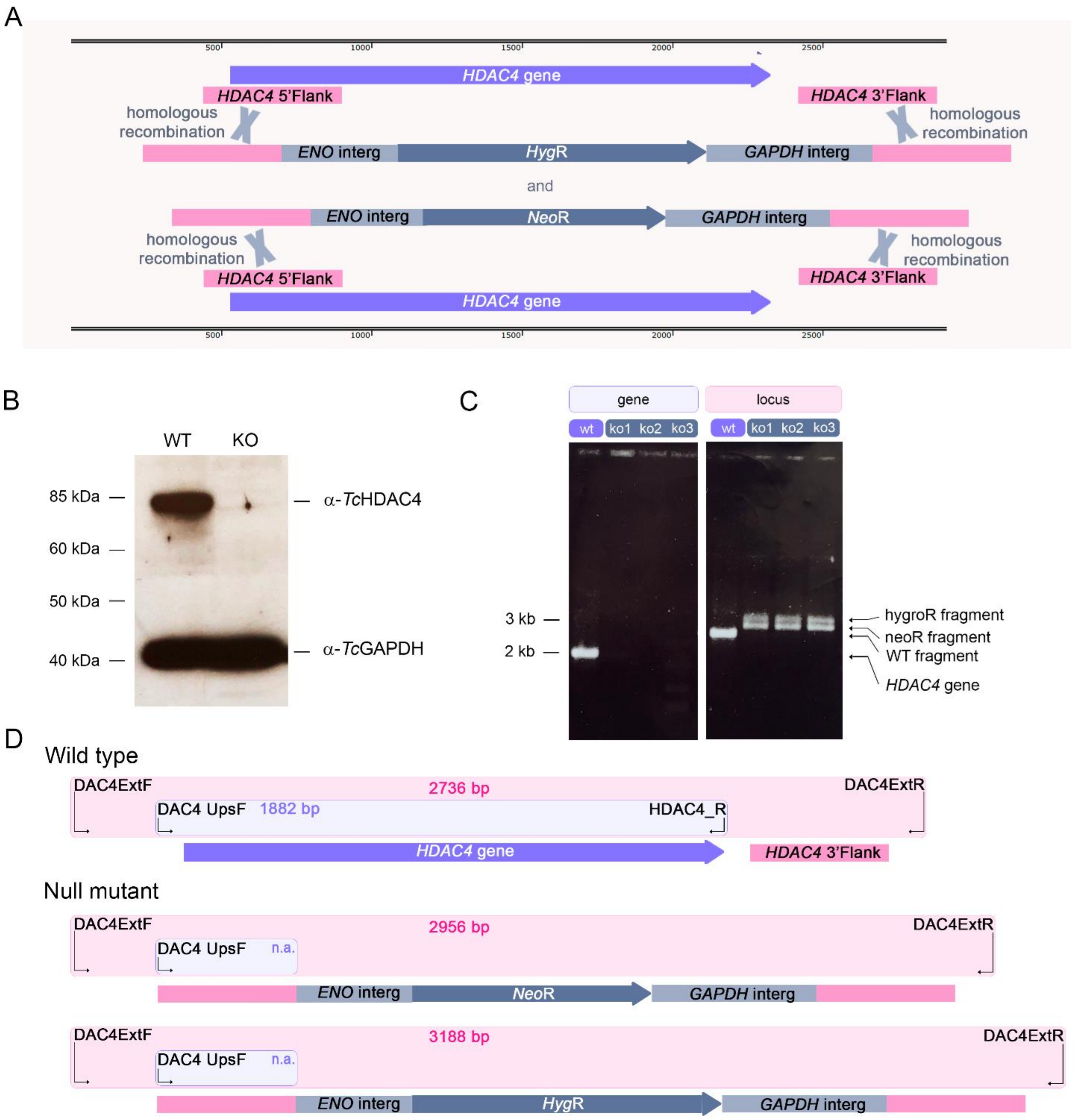

Figure S3. Generation of *Tc*HDAC4 null mutant parasites. (**A**) Scheme of *TcHDAC4* gene knockout by homologous recombination. Epimastigote *T. cruzi* cells were initially transfected with the hygromycin selection marker containing the 5' and 3' *Tc*HDAC4 flanking sequences. After selection, cells from the hygromycin-resistant population were transfected with the neomycin selection marker containing the 5' and 3' *Tc*HDAC4 flanking sequence and the cells were selected for resistance to both hygromycin and neomycin. (**B**) Western blot of wild type (WT) and null mutant (KO) cell extracts using antisera specific to detect *Tc*HDAC4 and *Tc*GAPDH. The latter was used as a loading control. Only the part of the immunoblot corresponding to position of the *Tc*HDAC4 and *Tc*GAPDH bands is shown. Complete image of the western blot is presented at the end of this document as Figure CI-1C (pink box Fig. CI-1C). (**C**) DNA gel electrophoresis of the PCR products of the analyses performed to confirm *Tc*HDAC4 knockout. A complete image of the gel electrophoreris is presented at the end of this document as Figure CI-3. The lanes identified by "gene" (white box in Fig. CI-3) correspond to the PCR reactions performed using oligonucleotides DAC4UpsF + DAC4R (Table S11).

Amplification of the gene is seen only in the wild type sample. The lanes identified by "locus" (yellow box in Fig. CI-3) correspond to the PCR reactions performed using primers DAC4ExtF + DAC4ExtR (Table S11). The single band in the WT sample indicates the presence of the original genome sequence. The two bands in the KO samples represent each of the selection makers used to knockout the two copies of the TcHDAC4 gene. (**D**) Scheme of the expected PCR amplification products for each primer set.

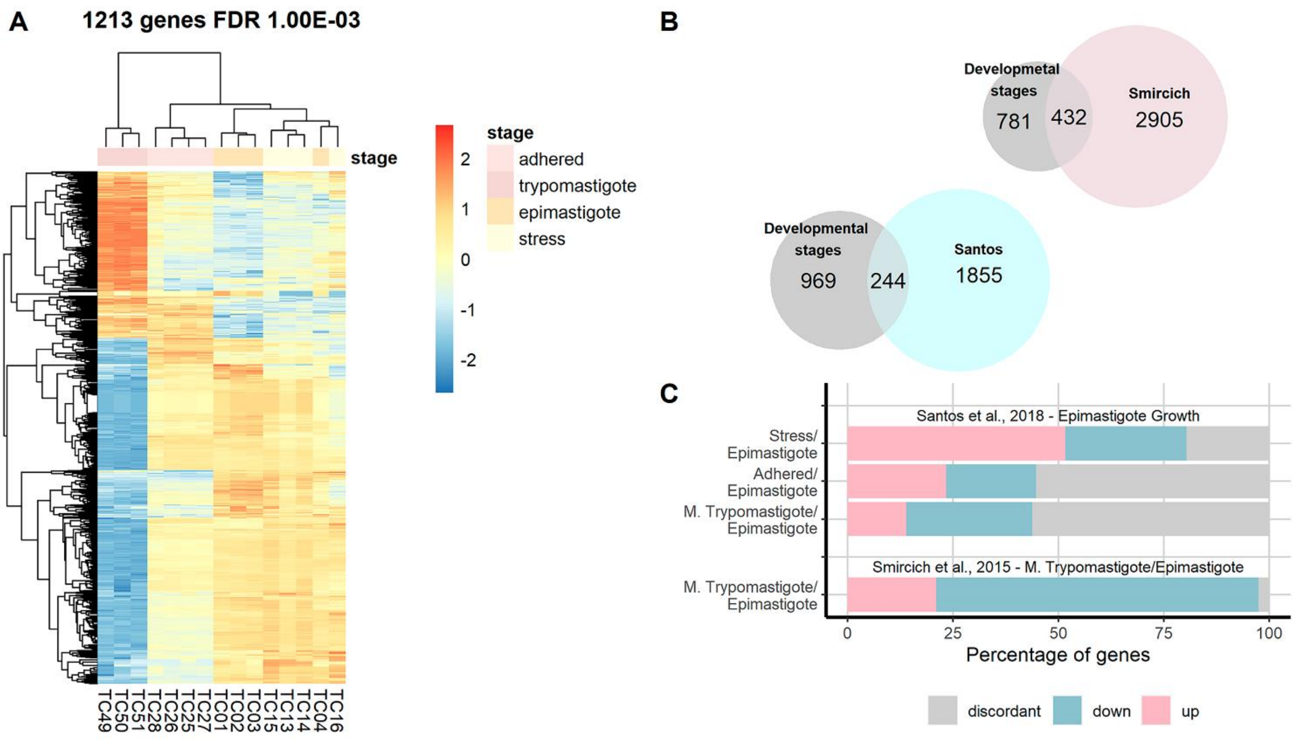

Figure S4. Dynamic changes in gene expression profile between different stages of wild type *T. cruzi* Dm28c. **(A)** Heatmap of 1,213 genes differentially expressed (FDR < 0.001) among the stages in wild type parasites. Each row represents a gene and the expression level of each gene is shown by a color scale of red and blue (z-score), which represents high and low expression, respectively. Each column represents a different sample, and the color above each sample represents the developmental stage. **(B)** Venn-diagram of genes identified in this study (grey circles) compared to two other studies: Santos and collaborators [8] (blue circle) and Smircich and collaborators [9] (pink circle). Developmental stages (grey circles) comprise all stages, using Epimastigote as reference. **(C)** Barplot of the percentage of differentially expressed genes with concordant and discordant patterns of change of expression. The y-axis shows the comparisons performed in this study and the x-axis the percentage of discordant (grey) and concordant changes in expression colored in pink or blue, which represent up and down regulated genes, respectively.

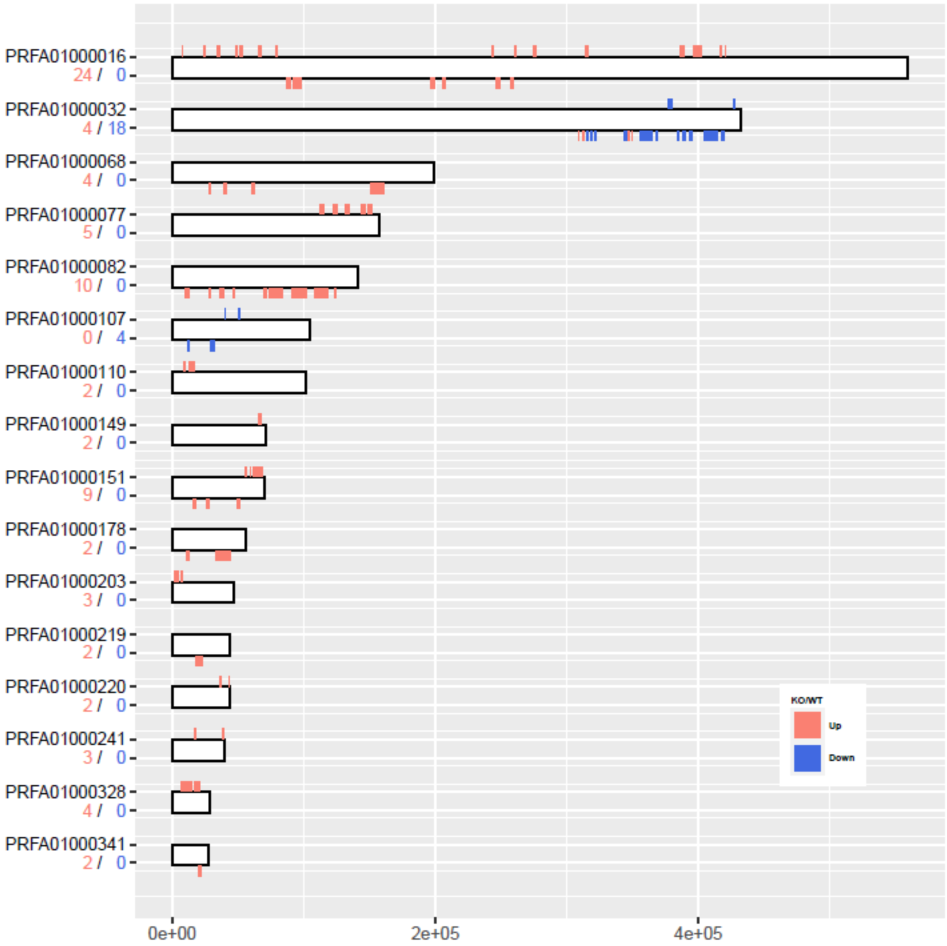

Figure S5. Clusters of differentially expressed genes in epimastigote stage along 16 overrepresented chromosomes. Bars represent the chromosomes scaled according to their sizes; the ID names of the chromosomes are on the left, with the number of differentially expressed genes indicated below each ID name. Only genes that were identified as differentially expressed are marked as red bars or blue bars, which mean up and down regulation (KO/WT), respectively. Colored bars that are above the chromosome indicate that the genes are encoded in the plus strand, and colored bars underneath the chromosome represent genes encoded in the minus strand.

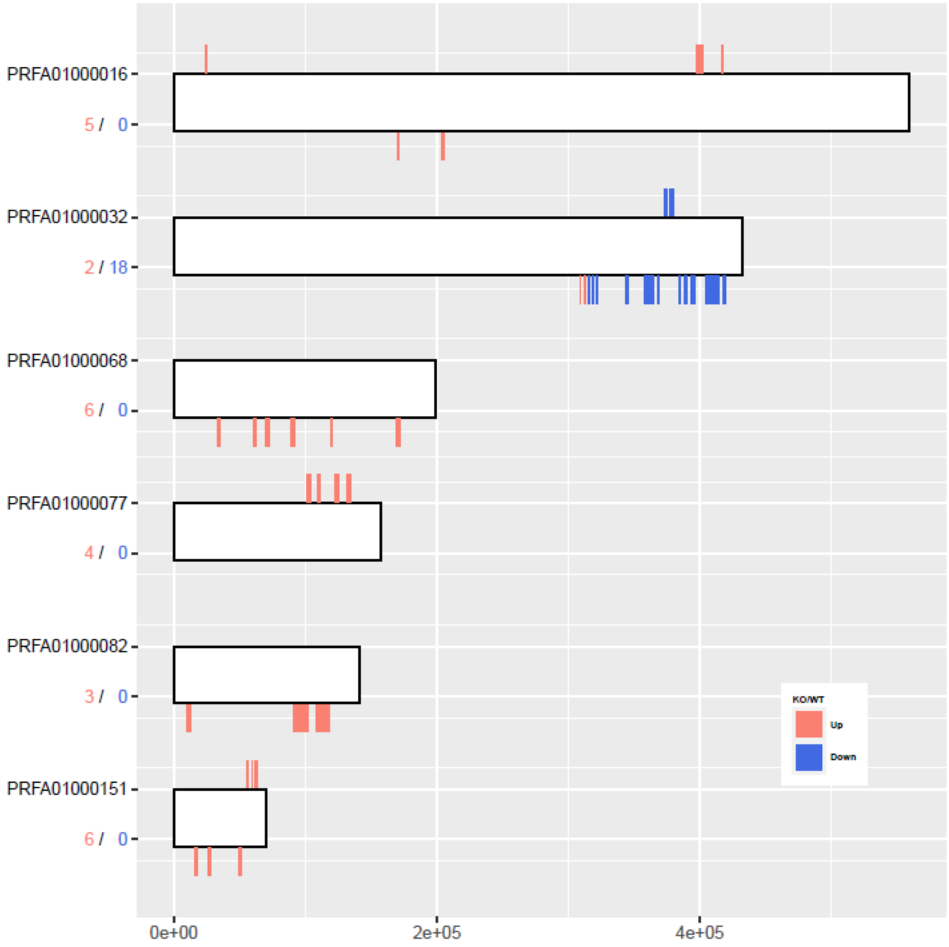

Figure S6. Clusters of differentially expressed genes in stressed epimastigotes along 6 overrepresented chromosomes. Bars represent the chromosomes scaled according to their sizes; the ID names of the chromosomes are on the left, with the number of differentially expressed genes indicated below each ID name. Only genes that were identified as differentially expressed are marked as red bars or blue bars, which means up and down regulation (KO/WT), respectively. Colored bars that are above the chromosome indicate that the genes are encoded in the plus strand, and colored bars underneath the chromosome represent genes encoded in the minus strand.

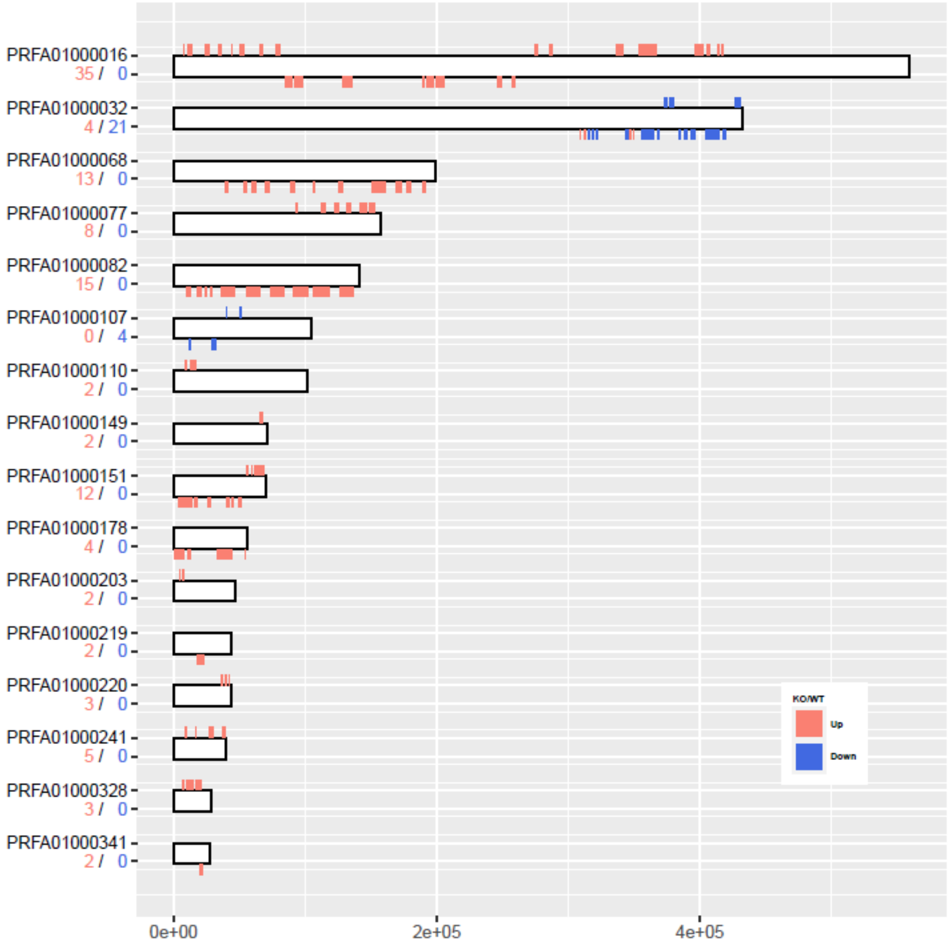

Figure S7. Clusters of differentially expressed genes in 24-h differentiating forms along 16 overrepresented chromosomes. Bars represent the chromosomes scaled according to their sizes; the ID names of the chromosomes are on the left, with the number of differentially expressed genes indicated below each ID name. Only genes that were identified as differentially expressed are marked as red bars or blue bars, which means up and down regulation (KO/WT), respectively. Colored bars that are above the chromosome indicate that the genes are encoded in the plus strand, and colored bars underneath the chromosome represent genes encoded in the minus strand.

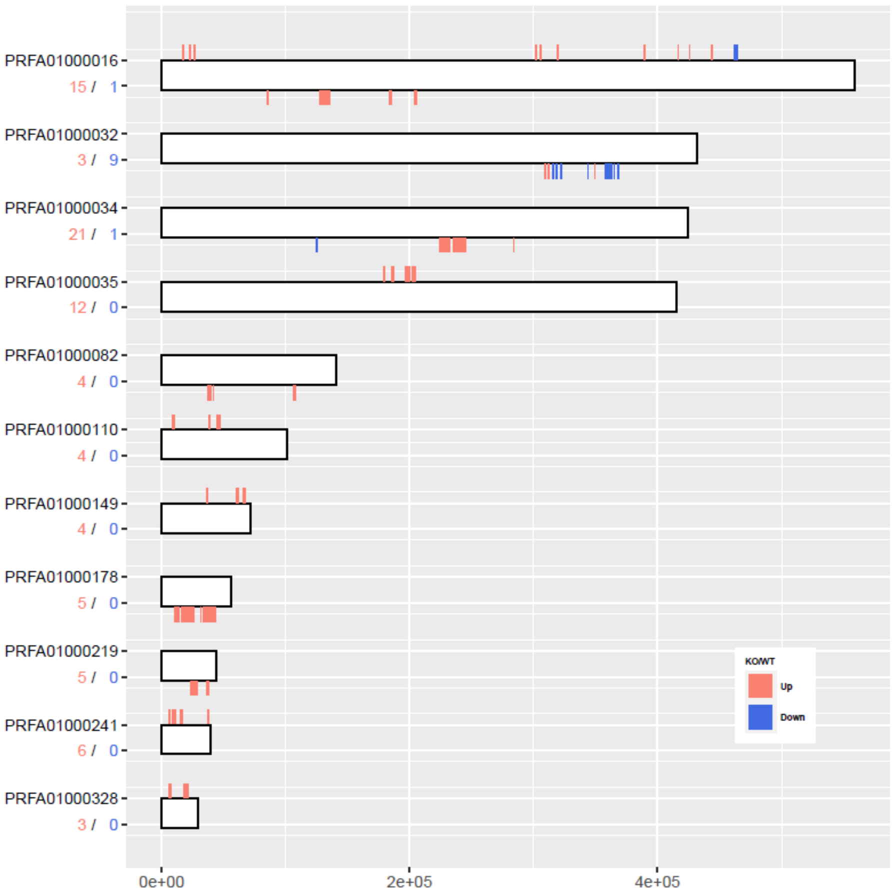

Figure S8. Clusters of differentially expressed genes in metacyclic trypomastigotes along 11 overrepresented chromosomes. Bars represent the chromosomes scaled according to their sizes; the ID names of the chromosomes are on the left, with the number of differentially expressed genes indicated below each ID name. Only genes that were identified as differentially expressed are marked as red bars or blue bars, which means up and down regulation (KO/WT), respectively. Colored bars that are above the chromosome indicate that the genes are encoded in the plus strand, and colored bars underneath the chromosome represent genes encoded in the minus strand.

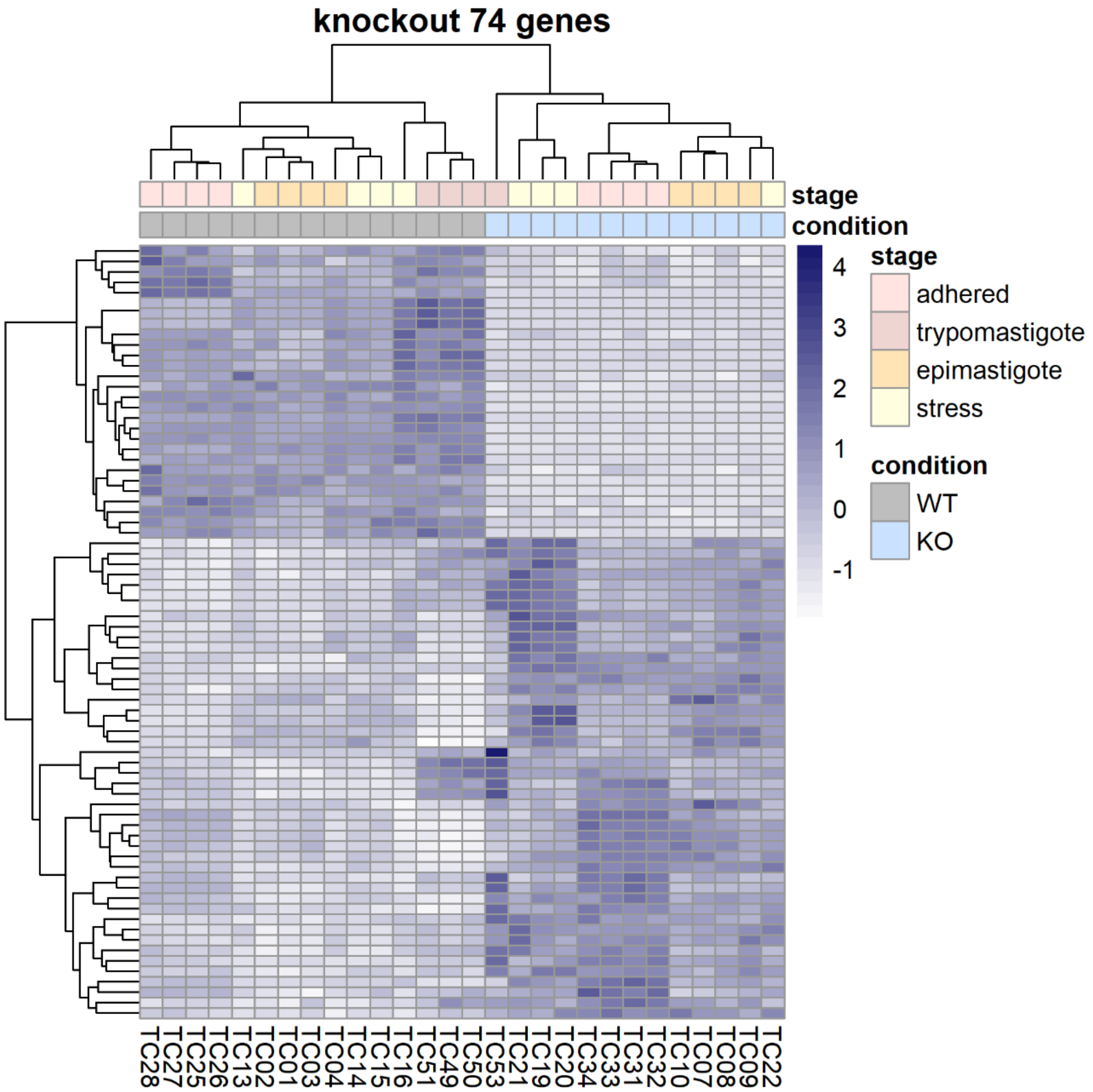

Figure S9. Unsupervised heatmap clustering of differentially expressed genes in KO vs WT parasites along all developmental stages. Each row represents a gene and, the expression levels are shown by the colored scale from white to blue (z-score scale at right), which represent low and high expression, respectively. Each column represents a sample and, the developmental stage and genetic condition (WT or KO) are assigned in the colored squares at the top.

Supplemental Methods

Genome assembly:

Five genome assembly versions identified as Dm28c 2014, Dm28c 2017, Dm28c 2018, SylvioX10-1-2012 and SylvioX10-1were retrieved from TriTrypDB (release 44) [1] for evaluation using the QUAST (v.5.0.2) [2] algorithm. The genomes were inspect concerning the number of contigs, N50, larger contig and ratio of coverage as shown in **Table SM1**. This evaluation indicated that the two best genome assemblies were obtained for Dm28c 2018 and SylvioX10-1. These two genomes were initially selected for further analyses.

Table SM1. Summary of genome assembly parameters using QUAST.

Assembly	TriTrypDB 44 Tcruzi Dm28c 2018 Genome	TriTrypDB 44 Tcruzi Dm28c 2017 Genome	TriTrypDB 44 Tcruzi Dm28c 2014 Genome	TriTrypDB 44 Tcruzi SylvioX10 1 Genome	TriTrypDB 44 Tcruzi SylvioX10 1 2012 Genome
#contigs	636	1029	1210	47	26891
Largest contig	1645565	1542890	462134	3116433	72500
Total length	53271887	50978736	27347664	41382871	38540861
GC (%)	51.56	51.69	50.55	51.57	51.16
N50	317638	110589	78389	1006492	2324
N75	79637	28414	19864	691987	797
L50	47	95	86	14	2588
L75	137	363	257	27	10934

Read mapping

RNA-Seq resulted on an average 15.45 million reads per sample. Quality of sequencing was evaluated with FASTQC (v0.11.7) [3]. 88% of bases presented >q30 quality score, which reflects the high sequencing quality. The FASTP (0.20.0) [4] algorithm was used to remove adapter sequences and short fragments (< 15 nt). After this filtering, about ~14.47 million (94.38%) reads per sample remained for further analyses. Reads were mapped to genome assemblies Dm28c 2018 or SylvioX10-1 using Bowtie (v. 2.2.9) [5] with default or very sensitive parameters. The overall mapping rate to the Dm28c 2018 genome were higher when compared with the overall mapping rate to the SylvioX10-1 genome. This was also observed using very sensitive parameters, which increased the multi mapping rate. In addition, Dm28c 2018 has 623 annotated SL sequences, while SylvioX10-1 had no SL sequences identified in its annotation file (release 44), which indicates that Dm28c 2018 has a better gene prediction annotation. Also, Dm28c 2018 assembly has 59 rRNA annotations, while SylvioX10-

1 presented only four rRNA annotations throughout the genome. Thus, Dm28c 2018 (release-44) assembly and annotation were used as references for further analyses.

Metacyclic Trypomastigote expression analysis:

Next, in order to identify the possible gene targets of *Tc*HDAC4, we have compared the gene expression pattern of wild type with *Tc*HDAC4 knockout cells during the different stages of metacyclogenesis in vitro. First, to confirm *Tc*HDAC4 knockout in the sequenced samples, we checked in the RNA-Seq data if all knockout samples showed no reads mapping at the *Tc*HDAC4 gene locus (C4B63_31g166-t42_1, Dm28c 2018). Indeed, *TcHDAC4* reads were absent in all developmental stage samples. However, in the metacyclic trypomastigote stage only one replicate corresponded to the *Tc*HDAC4 knockout (**Figure SM1**).

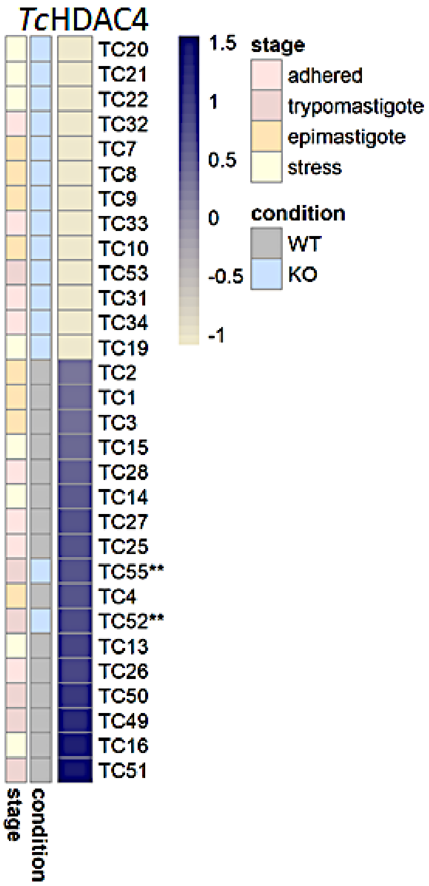

Figure SM1. Unsupervised heatmap clustering of *TcHDAC4* expression in each sample. Expression levels are shown as z-scores (-1 to 1.5) and are colored according to the scale at right from yellow to blue, which represent low and high expression, respectively. Developmental stage (stage) and genetic condition (condition, WT or KO) are indicated by two colored squares at the left side of each sample. ** samples that are clustered in the wrong group.

Unfortunately, obtaining new samples from knockout metacyclic trypomastigotes with the same method to replace those with problems was no longer possible since the DEAE resin used for parasite purifications was discontinued by the manufacturer (DEAE cellulose, D-3764, Sigma). Other types of matrix with the DEAE functional group have been tested but they were not as efficient to separate the metacyclic trypomastigotes and, using any other protocol to perform this separation could introduce unknown sources of non-biological bias in the expression analysis. Therefore, the analyses comprise four samples each of the wild type and null mutant from epimastigote, stressed epimastigote and 24 h adhered differentiating parasites whereas for the metacyclic trypomastigotes the samples comprised three wild type and only one null mutant. To perform the gene expression analysis of the metacyclic trypomastigotes, we used the NOISeq (v 2.28.0) [6,7] algorithm, which can handle assays without replicates. To adjust the analysis parameters, we used the biological coefficient of variation (BCV) function from EdgeR to calculate the overall variability using all 30 samples in the analysis, namely all samples of the developmental stages and genetic conditions (WT and KO). To calculate the subsample parameter, we tested the statistical analysis using from 10% up to 80% subsampled reads. Each test of subsampled reads size was repeated 10 times and the differentially expressed genes with FDR < 0.1 (probability 0.9) were calculated. The coefficient of variation (cv) was calculated for each point of subsampled reads size, and the smaller size that corresponded to a plateau in the graph (stabilized cv) was chosen (**Figure SM2**). It is possible to observe that the plateau begins at the 0.2 (20%) size of simulated subsamples.

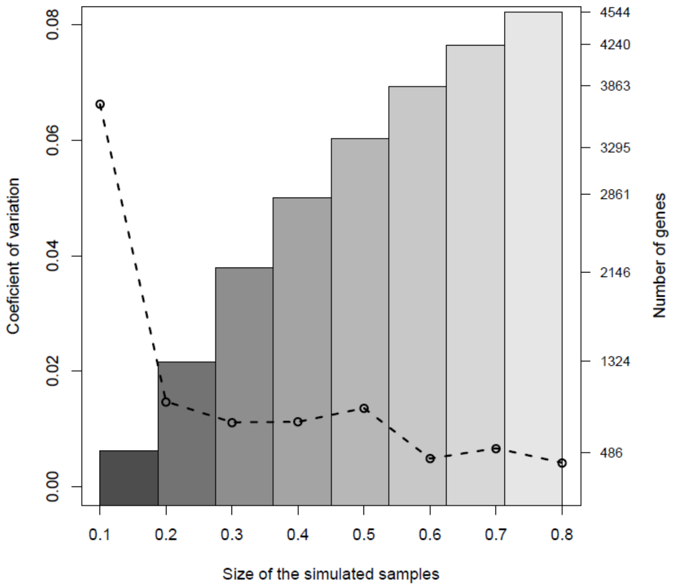

Figure SM2. Distribution of coefficient of variation (cv) as a function of the size of simulated samples. The y-axis at the left side shows the cv in each of 10 simulated tests performed at each size of simulated samples. The right side of the y-axis shows the mean value of number of genes identified as differentially expressed (FDR < 10%). The x-axis shows the size of simulated samples.

Subsequently, statistical analysis of differential expression (KO vs WT) was performed using subsample size = 0.2 and 5 subsample replicates, using an FDR < 0.05 (**Table SM2**). Thus, for each round in the analysis (KO vs WT) a set of 5 subsample replicates from each of three wild type samples (TC49 to TC 51) was compared to the 5 subsample replicates of knockout sample TC53. Ten different rounds of analyses were performed per each KO vs WT comparison, resulting in 30 different statistical analyses of differentially expressed genes (FDR < 0.05) (**Table SM2**). Only the overlapping differentially expressed genes (FDR < 0.05) that were identified in common among all 30 statistical analyses were selected for further analyses; the overall intersect resulted in 236 differentially expressed genes (FDR < 0.05) comparing KO versus WT (**Table SM2, see also Table 1**), which are listed in **Table S4**.

Table SM2. Number of significant differentially expressed genes in metacyclic trypomastigotes in each simulation (KO vs WT, FDR < 0.05).

Round # (5 subsamples per round)	TC53 vs TC49	TC53 vs TC50	TC53 vs TC51
1	429	633	445
2	439	575	441
3	429	575	433
4	537	624	460
5	442	609	421
6	488	615	433
7	480	590	437
8	381	587	428
9	481	542	453
10	445	570	424
**intersect**	**381**	**542**	**419**
**overall intersect**	**236**		

Supplemental References

[1] M. Aslett, C. Aurrecoechea, M. Berriman, J. Brestelli, B.P. Brunk, M. Carrington, D.P. Depledge, S. Fischer, B. Gajria, X. Gao, M.J. Gardner, A. Gingle, G. Grant, O.S. Harb, M. Heiges, C. Hertz-Fowler, R. Houston, F. Innamorato, J. Iodice, J.C. Kissinger, E. Kraemer, W. Li, F.J. Logan, J.A. Miller, S. Mitra, P.J. Myler, V. Nayak, C. Pennington, I. Phan, D.F. Pinney, G. Ramasamy, M.B. Rogers, D.S. Roos, C. Ross, D. Sivam, D.F. Smith, G. Srinivasamoorthy, C.J. Stoeckert, S. Subramanian, R. Thibodeau, A. Tivey, C. Treatman, G. Velarde, H. Wang, TriTrypDB: a functional genomic resource for the Trypanosomatidae., Nucleic Acids Res. 38 (2010) D457-62. https://doi.org/10.1093/nar/gkp851.

[2] A. Gurevich, V. Saveliev, N. Vyahhi, G. Tesler, QUAST: quality assessment tool for genome assemblies, Bioinformatics. 29 (2013) 1072–1075. https://doi.org/10.1093/bioinformatics/btt086.

[3] S. Andrews, FastQC: A quality control tool for high throughput sequence data, Babraham Bioinforma. (2010).

[4] S. Chen, Y. Zhou, Y. Chen, J. Gu, fastp: an ultra-fast all-in-one FASTQ preprocessor, Bioinformatics. 34 (2018) i884–i890. https://doi.org/10.1093/bioinformatics/bty560.

[5] B. Langmead, Aligning Short Sequencing Reads with Bowtie, in: Curr. Protoc. Bioinforma., John Wiley & Sons, Inc., Hoboken, NJ, USA, 2010. https://doi.org/10.1002/0471250953.bi1107s32.

[6] S. Tarazona, F. Garcia-Alcalde, J. Dopazo, A. Ferrer, A. Conesa, Differential expression in RNA-seq: A matter of depth, Genome Res. 21 (2011) 2213–2223. https://doi.org/10.1101/gr.124321.111.

[7] S. Tarazona, F. García, A. Ferrer, J. Dopazo, A. Conesa, NOIseq: a RNA-seq differential expression method robust for sequencing depth biases, EMBnet.Journal. 17 (2012) 18. https://doi.org/10.14806/ej.17.B.265.

[8] C.M.B. dos Santos, A. Ludwig, R.L. Kessler, R. de C.P. Rampazzo, A.H. Inoue, M.A. Krieger, D.P. Pavoni, C.M. Probst, Trypanosoma cruzi transcriptome during axenic epimastigote growth curve, Mem. Inst. Oswaldo Cruz. 113 (2018). https://doi.org/10.1590/0074-02760170404.

[9] P. Smircich, G. Eastman, S. Bispo, M.A. Duhagon, E.P. Guerra-Slompo, B. Garat, S. Goldenberg, D.J. Munroe, B. Dallagiovanna, F. Holetz, J.R. Sotelo-Silveira, Ribosome profiling reveals translation control as a key mechanism generating differential gene expression in Trypanosoma cruzi, BMC Genomics. 16 (2015) 443. https://doi.org/10.1186/s12864-015-1563-8.

Supplemental Tables

Table S1: Differentially expressed genes (FDR < 5%) in the Epimastigote analysis (KO vs WT)											
trancriptID(Dm28c2018)	chr	start	end	strand	description	Voom		Sleuth		EdgeR	
						Log2 (KO/WT)	adjP	b	qval	Log2 (KO/WT)	FDR
C4B63_104g57-t42_1	PRFA01000104	89353	90504	-	UDP-Gal or UDP-GlcNAc-dependent glycosyltransferase	2,546878314	0,001692	1,528807	0,000265	2,320067438	2,03E-05
C4B63_107g104-t42_1	PRFA01000107	12288	13130	-	Mucin-associated surface protein (MASP)	-3,47658724	0,001134	-2,41747	0,000769	-3,246612871	9,72E-05
C4B63_107g29-t42_1	PRFA01000107	39859	40764	+	unspecified product	-1,556114937	0,013136	-1,11757	0,024461	-1,906158807	0,000282
C4B63_107g32-t42_1	PRFA01000107	50807	51955	+	Mucin-associated surface protein (MASP)	-2,274763895	0,009388	-1,67762	0,004965	-2,372705158	0,000113
C4B63_107g93-t42_1	PRFA01000107	29245	32070	-	trans-sialidase%2C Group VI	-1,329294099	0,009212	-1,03088	5,27E-05	-1,645174128	2,69E-05
C4B63_110g7-t42_1	PRFA01000110	8575	10197	+	multidrug resistance protein E	0,579899482	0,004613	0,452474	4,75E-05	0,716958925	0,00018
C4B63_110g9-t42_1	PRFA01000110	12686	16531	+	conserved hypothetical protein	0,571576312	0,004031	0,433682	2,59E-07	0,70194017	4,01E-06
C4B63_12g221-t42_1	PRFA01000012	449058	451799	-	pyruvate phosphate dikinase	-1,064477403	0,00214	-0,67431	0,000772	-0,839554366	0,000284
C4B63_149g40-t42_1	PRFA01000149	65397	66239	+	unspecified product	0,925571372	0,002232	0,689249	9,61E-09	0,912488618	0,000103
C4B63_149g41-t42_1	PRFA01000149	66509	67450	+	conserved hypothetical protein	0,844197084	0,005288	0,541947	2,56E-05	1,005736716	4,53E-06
C4B63_151g29-t42_1	PRFA01000151	55419	56318	+	conserved hypothetical protein	1,136093311	0,000656	0,741609	2,59E-08	0,899706848	4,80E-07
C4B63_151g31-t42_1	PRFA01000151	59428	59940	+	conserved hypothetical protein	0,813152968	0,002306	0,611935	4,42E-11	0,722330457	0,000142
C4B63_151g33-t42_1	PRFA01000151	61145	63370	+	cullin	1,125189909	0,000289	0,766494	5,12E-14	1,020907715	2,00E-06
C4B63_151g34-t42_1	PRFA01000151	63628	66042	+	TFIIH basal transcription factor complex helicase subunit	1,074954078	0,000289	0,692099	2,71E-11	0,962358572	1,94E-06
C4B63_151g35-t42_1	PRFA01000151	66166	66990	+	zinc-finger multi-pass transmembrane protein	1,363943721	0,00086	0,86208	1,71E-05	1,211589358	4,52E-06
C4B63_151g36-t42_1	PRFA01000151	67366	68874	+	notchless homolog	1,125180107	0,001305	0,741531	0,002223	1,129755726	5,25E-05
C4B63_151g38-t42_1	PRFA01000151	49425	50972	-	unspecified product	1,208230266	0,00079	0,803192	6,54E-13	1,062715328	3,68E-06
C4B63_151g47-t42_1	PRFA01000151	26076	28088	-	unspecified product	0,862416966	0,000404	0,614456	4,91E-10	0,88467857	1,75E-05
C4B63_151g48-t42_1	PRFA01000151	16457	18292	-	retrotransposon hot spot (RHS) protein	0,887538366	0,004631	0,683063	5,32E-05	1,090871415	1,73E-07
C4B63_16g105-t42_1	PRFA01000016	314483	315979	+	coronin	0,426427149	0,006809	0,309401	0,00073	0,503118067	0,000294
C4B63_16g11-t42_1	PRFA01000016	23961	24878	+	5'-AMP-activated protein kinase subunit beta	0,919437945	0,003322	0,601834	3,73E-08	0,868982413	0,000152
C4B63_16g132-t42_1	PRFA01000016	385925	388921	+	DNA polymerase theta (helicase domain only)	0,414820678	0,022832	0,306849	0,001077	0,519149834	0,000365
C4B63_16g139-t42_1	PRFA01000016	396139	397056	+	palmitoyl acyltransferase 12	0,752544804	0,003044	0,504528	1,25E-07	0,71957198	0,000199
C4B63_16g140-t42_1	PRFA01000016	397584	402098	+	Paladin	0,734417256	0,000816	0,492472	3,44E-09	0,633166989	4,14E-05
C4B63_16g147-t42_1	PRFA01000016	417079	417951	+	Ring finger domain containing protein	0,69346735	0,001305	0,518409	2,31E-08	0,757275224	1,69E-06
C4B63_16g148-t42_1	PRFA01000016	420214	420918	+	vesicle transport v-SNARE 11	0,781492192	0,003709	0,458078	0,000643	0,728301675	4,35E-05
C4B63_16g17-t42_1	PRFA01000016	34316	36349	+	acetyl-CoA synthetase	0,488852638	0,001812	0,352873	5,32E-05	0,511328832	0,000319
C4B63_16g25-t42_1	PRFA01000016	48149	48982	+	conserved hypothetical protein	0,51874519	0,023881	0,373321	0,001496	0,66232054	0,00026
C4B63_16g269-t42_1	PRFA01000016	257345	259813	-	conserved hypothetical protein	1,104873259	0,003446	0,634633	0,005411	0,842858987	0,00016
C4B63_16g274-t42_1	PRFA01000016	246107	249091	-	conserved hypothetical protein	0,842165765	0,001275	0,536124	1,04E-06	0,682764148	0,000115
C4B63_16g279-t42_1	PRFA01000016	205879	207162	-	S-adenosyl-L-methionine-dependent methyltransferase	0,888406612	0,004631	0,559754	0,000566	0,618104274	0,000202
C4B63_16g28-t42_1	PRFA01000016	51922	53466	+	Protein X92	0,664816578	0,022832	0,491095	0,00058	0,82233988	0,000224
C4B63_16g282-t42_1	PRFA01000016	198059	199240	-	SET domain containing protein	0,690247996	0,001305	0,437749	9,25E-06	0,688896999	9,26E-05
C4B63_16g284-t42_1	PRFA01000016	196229	197785	-	actin interacting protein-like protein	0,496616417	0,026629	0,401739	0,000367	0,57846662	0,000217
C4B63_16g3-t42_1	PRFA01000016	7473	7979	+	aldose 1-epimerase-like protein	0,642296917	0,002237	0,467558	1,62E-05	0,682416246	0,000244
C4B63_16g329-t42_1	PRFA01000016	95141	97729	-	conserved hypothetical protein	0,59205727	0,011339	0,45571	0,000339	0,710863885	5,41E-05
C4B63_16g33-t42_1	PRFA01000016	65468	67915	+	trans-sialidase%2C Group VIII	0,886523929	0,003115	0,542016	0,003498	0,676176386	0,000108
C4B63_16g330-t42_1	PRFA01000016	92335	94587	-	kinesin-C	0,742081135	0,001495	0,483534	3,76E-09	0,729617268	4,18E-05

C4B63_16g331-t42_1	PRFA01000016	87043	90159	-	ubiquitin-activating enzyme E1	0,645232561	0,032387	0,502272	0,021746	0,940556879	5,97E-07
C4B63_16g36-t42_1	PRFA01000016	78767	79936	+	unspecified product	0,874806409	0,037812	0,513946	0,037528	1,005448937	5,90E-05
C4B63_16g78-t42_1	PRFA01000016	243216	244145	+	unspecified product	1,052833319	0,003482	0,687918	1,12E-05	0,846140762	0,000268
C4B63_16g79-t42_1	PRFA01000016	260196	261740	+	ammonium transporter	0,540853653	0,005199	0,377102	8,97E-06	0,581037414	0,000304
C4B63_16g86-t42_1	PRFA01000016	274837	276630	+	mannosyl-oligosaccharide 1%2C2-alpha-mannosidase IB	0,595563213	0,003044	0,38201	5,21E-05	0,661994424	1,86E-05
C4B63_178g41-t42_1	PRFA01000178	33472	43860	-	dispersed gene family protein 1 (DGF-1)	0,982270563	0,000174	0,619859	1,21E-13	0,92965691	9,60E-07
C4B63_178g46-t42_1	PRFA01000178	10396	12861	-	conserved hypothetical protein	0,644541186	0,001291	0,413928	0,000141	0,588420784	0,000327
C4B63_17g327-t42_1	PRFA01000017	10819	11910	-	retrotransposon hot spot (RHS) protein	0,870050728	0,004631	0,641377	5,44E-08	0,818442893	0,000164
C4B63_17g328-t42_1	PRFA01000017	9996	10877	-	retrotransposon hot spot protein (RHS)	1,184718828	0,003482	0,824536	1,47E-06	1,321694351	4,06E-05
C4B63_17g329-t42_1	PRFA01000017	9182	9931	-	retrotransposon hot spot protein (RHS)	1,037435165	0,021358	0,799086	0,000481	1,132963963	0,000102
C4B63_182g5-t42_1	PRFA01000182	8170	18678	+	dispersed gene family protein 1 (DGF-1)	0,572396154	0,015982	0,415414	0,000384	0,74225342	2,27E-05
C4B63_1g812-t42_1	PRFA01000001	1412620	1413180	-	mucin TcMUCII	-3,149905623	0,000578	-1,99451	0,036072	-3,499565829	3,15E-05
C4B63_1g818-t42_1	PRFA01000001	1403985	1405280	-	Mucin-associated surface protein (MASP)	-1,858926062	0,004631	-1,22152	0,001247	-1,938432301	0,000246
C4B63_1g819-t42_1	PRFA01000001	1400040	1402877	-	trans-sialidase%2C Group VI	-0,973464103	0,015887	-0,74678	0,000339	-1,17706612	5,05E-05
C4B63_203g1-t42_1	PRFA01000203	1584	2864	+	unspecified product	0,561900523	0,004125	0,390412	0,000146	0,59919258	0,000324
C4B63_203g3-t42_1	PRFA01000203	4056	4520	+	unspecified product	0,752704509	0,01641	0,583858	0,000495	0,844859749	0,000229
C4B63_203g5-t42_1	PRFA01000203	6569	7513	+	conserved hypothetical protein	0,529664845	0,03516	0,362842	0,011161	0,663554708	0,000301
C4B63_219g47-t42_1	PRFA01000219	20336	22567	-	multidrug resistance-associated protein(fragment)	0,569009468	0,013136	0,452237	0,001247	0,714441787	2,72E-05
C4B63_219g48-t42_1	PRFA01000219	17822	20293	-	multidrug resistance protein E	0,509598778	0,016162	0,394556	0,00073	0,661693443	1,77E-05
C4B63_220g14-t42_1	PRFA01000220	43025	43594	+	conserved hypothetical protein	0,866062047	0,002622	0,546292	2,65E-05	0,715420142	0,000397
C4B63_220g9-t42_1	PRFA01000220	36218	37525	+	conserved hypothetical protein	0,742154996	0,000598	0,53545	4,00E-09	0,755961629	2,54E-05
C4B63_241g14-t42_1	PRFA01000241	16601	17215	+	conserved hypothetical protein	0,61456079	0,01023	0,473019	0,000252	0,730641125	0,000195
C4B63_241g15-t42_1	PRFA01000241	17674	18237	+	terbinafine resistance locus protein (yip1)	0,614307664	0,025187	0,504897	0,023067	0,837717076	2,18E-05
C4B63_241g24-t42_1	PRFA01000241	38531	39475	+	conserved hypothetical protein	0,543840395	0,007063	0,41388	1,34E-05	0,643976459	2,34E-05
C4B63_256g19-t42_1	PRFA01000256	12069	22508	-	dispersed gene family protein 1 (DGF-1)	1,112634254	0,000536	0,789655	2,08E-10	1,139634682	1,32E-05
C4B63_31g166-t42_1	PRFA01000031	388657	390453	-	histone deacetylase 4	-8,827122872	1,01E-06	-5,96902	3,68E-41	-9,561266767	1,28E-11
C4B63_31g167-t42_1	PRFA01000031	387153	388403	-	conserved hypothetical protein	-0,829881697	0,002455	-0,57861	7,08E-07	-0,883782574	5,40E-05
C4B63_328g10-t42_1	PRFA01000328	10231	14976	+	multidrug resistance protein E	0,646667623	0,014118	0,498126	0,001772	0,812827128	2,87E-05
C4B63_328g12-t42_1	PRFA01000328	17465	21298	+	conserved hypothetical protein	0,671384226	0,001692	0,465129	3,73E-08	0,621661002	0,000161
C4B63_328g8-t42_1	PRFA01000328	6842	7456	+	conserved hypothetical protein	0,61456079	0,01023	0,473019	0,000252	0,730641125	0,000195
C4B63_328g9-t42_1	PRFA01000328	7910	8473	+	terbinafine resistance locus protein (yip1)	0,614307664	0,025187	0,504897	0,023067	0,837717076	2,18E-05
C4B63_32g139-t42_1	PRFA01000032	377181	380183	+	trans-sialidase%2C Group VI	-1,246399159	0,002622	-0,86646	2,01E-07	-1,401423527	1,80E-05
C4B63_32g171-t42_1	PRFA01000032	426951	427727	+	unspecified product	-5,274132025	0,005878	-3,46471	0,003707	-5,31411251	0,000184
C4B63_32g177-t42_1	PRFA01000032	417391	419745	-	unspecified product	-5,417583738	0,000404	-3,94209	1,36E-14	-5,472656032	1,86E-07
C4B63_32g181-t42_1	PRFA01000032	404401	414789	-	dispersed gene family protein 1 (DGF-1)	-7,228750648	1,01E-06	-5,05306	1,86E-45	-7,674306333	6,35E-10
C4B63_32g186-t42_1	PRFA01000032	393324	394817	-	Mucin-associated surface protein (MASP)	-6,443232078	1,23E-05	-4,28969	8,96E-21	-7,183671666	7,59E-08
C4B63_32g187-t42_1	PRFA01000032	387776	390559	-	trans-sialidase%2C Group V	-2,791993952	0,001305	-1,77188	3,77E-05	-3,168213876	5,72E-06
C4B63_32g189-t42_1	PRFA01000032	384386	385171	-	unspecified product	-6,283418859	2,87E-06	-4,34	1,14E-18	-8,16631311	5,01E-06
C4B63_32g198-t42_1	PRFA01000032	368205	369104	-	conserved hypothetical protein	-7,508591687	1,83E-05	-5,35634	2,51E-17	-7,313945688	1,21E-11
C4B63_32g200-t42_1	PRFA01000032	364648	365160	-	conserved hypothetical protein	-7,415684894	1,25E-05	-5,03128	9,00E-21	-7,784338665	6,82E-10
C4B63_32g202-t42_1	PRFA01000032	361204	363429	-	cullin	-9,513682006	1,27E-06	-6,73295	5,15E-214	-11,58968288	3,87E-10
C4B63_32g203-t42_1	PRFA01000032	358525	360939	-	TFIIH basal transcription factor complex helicase subunit	-8,321121871	0,000275	-6,12094	2,98E-12	-7,702785237	2,24E-09
C4B63_32g204-t42_1	PRFA01000032	357574	358398	-	zinc-finger multi-pass transmembrane protein	-7,364922731	0,000289	-5,44643	2,24E-14	-7,274863682	7,92E-09

C4B63_32g205-t42_1	PRFA01000032	355690	357255	-	notchless homolog	-1,230845535	0,001216	-0,75866	1,54E-06	-1,080479934	0,000108
C4B63_32g211-t42_1	PRFA01000032	349223	349717	-	60S ribosomal protein L12	0,825671408	0,000289	0,549102	1,44E-12	0,833199751	4,00E-06
C4B63_32g212-t42_1	PRFA01000032	346945	347817	-	Pentatricopeptide repeat-containing protein At2g31400%2C chloroplastic	0,641368863	0,002622	0,447783	7,24E-07	0,686442203	8,96E-05
C4B63_32g214-t42_1	PRFA01000032	344653	345867	-	2-amino-3-ketobutyrate coenzyme A ligase	-1,764552577	0,006688	-1,03951	0,004317	-1,549575022	6,74E-06
C4B63_32g215-t42_1	PRFA01000032	343548	344264	-	Pentatricopeptide repeat-containing protein At2g31400%2C chloroplastic	-8,230068753	2,54E-06	-5,83999	2,28E-95	-10,96354522	6,43E-09
C4B63_32g226-t42_1	PRFA01000032	321634	322218	-	amastin	-8,286197471	1,01E-06	-5,28262	1,16E-65	-10,5258195	7,25E-09
C4B63_32g227-t42_1	PRFA01000032	318439	319023	-	amastin	-8,286197471	1,01E-06	-5,28262	1,16E-65	-10,5258195	7,25E-09
C4B63_32g228-t42_1	PRFA01000032	315244	315828	-	amastin	-8,286197471	1,01E-06	-5,28262	1,16E-65	-10,5258195	7,25E-09
C4B63_32g229-t42_1	PRFA01000032	312055	312639	-	amastin	1,241122661	0,000487	0,791742	4,66E-08	1,007623751	1,06E-06
C4B63_32g230-t42_1	PRFA01000032	308861	309445	-	amastin	1,241122661	0,000487	0,791742	4,66E-08	1,007623751	1,06E-06
C4B63_341g11-t42_1	PRFA01000341	21156	22202	-	lipase	1,087185428	0,009388	0,723521	0,000506	1,280727185	4,26E-05
C4B63_341g12-t42_1	PRFA01000341	19737	20783	-	lipase	0,918233983	0,001305	0,698244	8,44E-06	0,960923001	0,000123
C4B63_43g98-t42_1	PRFA01000043	356572	357820	+	unspecified product	1,308551044	0,003482	0,779272	0,002308	1,200209416	2,36E-05
C4B63_68g109-t42_1	PRFA01000068	60539	62383	-	retrotransposon hot spot protein (RHS)	0,805172156	0,000289	0,532889	9,61E-09	0,706299878	2,81E-05
C4B63_68g120-t42_1	PRFA01000068	39113	41554	-	retrotransposon hot spot (RHS) protein	0,806418462	9,20E-05	0,54759	9,07E-13	0,745043016	2,00E-06
C4B63_68g123-t42_1	PRFA01000068	28004	28756	-	retrotransposon hot spot protein (RHS)	0,947395677	0,001305	0,60428	1,34E-05	0,778505302	0,000272
C4B63_68g83-t42_1	PRFA01000068	151070	161440	-	dispersed gene family protein 1 (DGF-1)	0,685755212	0,003983	0,415809	5,78E-06	0,709968976	0,000164
C4B63_77g53-t42_1	PRFA01000077	112536	115550	+	trans-sialidase%2C Group III	0,792893093	0,00214	0,513189	1,56E-07	0,636790539	0,000289
C4B63_77g60-t42_1	PRFA01000077	122003	125029	+	trans-sialidase%2C Group III	0,789467436	0,001863	0,537079	4,91E-10	0,733417158	0,000139
C4B63_77g67-t42_1	PRFA01000077	131426	134452	+	trans-sialidase%2C Group III	0,761560452	0,002212	0,47972	1,28E-05	0,65771218	7,83E-05
C4B63_77g75-t42_1	PRFA01000077	143718	147230	+	ABC transporter	0,825770294	0,000289	0,571909	4,36E-13	0,822295022	6,39E-06
C4B63_77g77-t42_1	PRFA01000077	148417	151488	+	conserved hypothetical protein	0,773517526	0,000127	0,532753	4,91E-10	0,762052452	2,45E-06
C4B63_82g107-t42_1	PRFA01000082	27776	28831	-	NADP-dependent alcohol hydrogenase	0,623573876	0,003853	0,471654	7,36E-07	0,73543042	2,72E-05
C4B63_82g114-t42_1	PRFA01000082	10099	12477	-	unspecified product	0,83633837	0,000816	0,536688	9,01E-09	0,770744172	3,05E-05
C4B63_82g448c-t42_1	PRFA01000082	36387	36749	-	conserved hypothetical protein	0,790678824	0,002341	0,530812	6,47E-09	0,65355641	0,000225
C4B63_82g72-t42_1	PRFA01000082	123723	124337	-	tryptophanyl-tRNA synthetase	1,178891486	0,00214	0,773706	0,001227	1,182247861	0,000221
C4B63_82g75-t42_1	PRFA01000082	108396	118167	-	unspecified product	0,892437063	0,000327	0,569889	1,90E-11	0,798101673	2,97E-06
C4B63_82g81-t42_1	PRFA01000082	91424	101914	-	dispersed gene family protein 1 (DGF-1)	0,533886658	0,041628	0,425074	0,012704	0,719966664	3,37E-05
C4B63_82g85-t42_1	PRFA01000082	73787	84286	-	dispersed gene family protein 1 (DGF-1)	0,575930806	0,031284	0,454406	0,015163	0,673157276	8,99E-05
C4B63_82g86-t42_1	PRFA01000082	70147	71316	-	tryptophanyl-tRNA synthetase	0,821204041	0,004191	0,625638	0,013286	0,814426189	0,000421
C4B63_82g93-t42_1	PRFA01000082	46549	47211	-	conserved hypothetical protein	0,567746065	0,006964	0,469687	0,000405	0,692166605	0,000358
C4B63_82g98-t42_1	PRFA01000082	37040	38827	-	glycosyl transferase	0,504469978	0,036229	0,450203	0,022757	0,691518477	4,20E-05
C4B63_85g45-t42_1	PRFA01000085	109007	109615	-	ribosomal protein S7	0,65315173	0,003819	0,424298	4,86E-06	0,676864493	0,000214

Table S2: Differentially expressed genes (FDR < 5%) in the Stress analysis (KO vs WT)											
trancriptID(Dm28c2018)	chr	start	end	strand	description	Voom		Sleuth		EdgeR	
						Log2 (KO/WT)	adjP	b	qval	Log2 (KO/WT)	FDR
C4B63_107g93-t42_1	PRFA01000107	29245	32070	-	trans-sialidase%2C Group VI	-1,435127862	0,004315	-1,11845	8,89E-06	-1,779060046	1,22E-05
C4B63_110g7-t42_1	PRFA01000110	8575	10197	+	multidrug resistance protein E	0,65365142	0,000518	0,478355	5,20E-05	0,762149284	5,27E-05
C4B63_151g29-t42_1	PRFA01000151	55419	56318	+	conserved hypothetical protein	1,314306083	0,000255	0,765264	0,000142	0,893228928	1,72E-05
C4B63_151g31-t42_1	PRFA01000151	59428	59940	+	conserved hypothetical protein	1,144058768	0,000217	0,691131	9,11E-06	0,852654204	0,000132
C4B63_151g33-t42_1	PRFA01000151	61145	63370	+	cullin	1,080173904	0,00102	0,739857	4,81E-08	1,151997743	6,44E-05
C4B63_151g38-t42_1	PRFA01000151	49425	50972	-	unspecified product	1,205613187	0,000359	0,739117	2,37E-06	0,874106646	0,000122
C4B63_151g47-t42_1	PRFA01000151	26076	28088	-	unspecified product	1,139096166	0,000241	0,707386	1,44E-12	0,993326802	0,000125
C4B63_151g48-t42_1	PRFA01000151	16457	18292	-	retrotransposon hot spot (RHS) protein	0,906824014	0,003117	0,710411	6,19E-06	1,109580056	8,00E-05
C4B63_16g11-t42_1	PRFA01000016	23961	24878	+	5'-AMP-activated protein kinase subunit beta	1,043701155	8,95E-05	0,658533	1,09E-11	0,878644987	6,76E-05
C4B63_16g140-t42_1	PRFA01000016	397584	402098	+	Paladin	0,892712663	0,000131	0,591264	1,60E-10	0,7434173	3,23E-05
C4B63_16g147-t42_1	PRFA01000016	417079	417951	+	Ring finger domain containing protein	0,659253407	0,002058	0,510408	1,19E-06	0,818362942	7,12E-05
C4B63_16g280-t42_1	PRFA01000016	203610	205595	-	conserved hypothetical protein	1,045617114	0,000467	0,623193	0,000683	0,777163327	4,98E-05
C4B63_16g295-t42_1	PRFA01000016	170549	171538	-	RNA-binding protein	1,163041609	0,000374	0,686439	0,000414	0,753274269	6,89E-05
C4B63_1g818-t42_1	PRFA01000001	1403985	1405280	-	Mucin-associated surface protein (MASP)	-1,978876903	0,002046	-1,25481	0,00263	-1,982633018	0,000158
C4B63_1g819-t42_1	PRFA01000001	1400040	1402877	-	trans-sialidase%2C Group VI	-1,155125559	0,002021	-0,81936	1,19E-07	-1,20094636	0,000178
C4B63_31g166-t42_1	PRFA01000031	388657	390453	-	histone deacetylase 4	-8,201818024	1,16E-05	-6,07267	7,18E-26	-8,685782153	6,79E-10
C4B63_328g10-t42_1	PRFA01000328	10231	14976	+	multidrug resistance protein E	0,502159535	0,03607	0,433554	0,036361	0,771708315	0,000168
C4B63_32g138-t42_1	PRFA01000032	372866	375253	+	unspecified product	-4,165968567	0,002353	-3,23369	6,19E-06	-4,661146453	3,14E-05
C4B63_32g139-t42_1	PRFA01000032	377181	380183	+	trans-sialidase%2C Group VI	-1,075677844	0,003117	-0,7866	1,04E-08	-1,406277926	2,21E-06
C4B63_32g177-t42_1	PRFA01000032	417391	419745	-	unspecified product	-5,530660665	8,95E-05	-4,05914	3,10E-19	-6,54869232	2,81E-08
C4B63_32g181-t42_1	PRFA01000032	404401	414789	-	dispersed gene family protein 1 (DGF-1)	-7,547271741	3,43E-07	-5,0147	4,00E-27	-7,536037154	3,27E-08
C4B63_32g185-t42_1	PRFA01000032	395454	396209	-	unspecified product	-3,640380653	0,001615	-2,84452	3,75E-05	-4,494561351	5,00E-05
C4B63_32g186-t42_1	PRFA01000032	393324	394817	-	Mucin-associated surface protein (MASP)	-6,544910176	2,35E-07	-4,59906	6,17E-25	-8,630136883	3,73E-06
C4B63_32g187-t42_1	PRFA01000032	387776	390559	-	trans-sialidase%2C Group V	-2,66259981	0,000253	-1,76819	0,000204	-2,879921642	5,36E-06
C4B63_32g189-t42_1	PRFA01000032	384386	385171	-	unspecified product	-6,038231618	3,43E-07	-4,30552	2,16E-17	-8,361147395	6,91E-06
C4B63_32g198-t42_1	PRFA01000032	368205	369104	-	conserved hypothetical protein	-7,705461195	3,43E-07	-5,2503	6,17E-25	-8,166848619	1,87E-12
C4B63_32g200-t42_1	PRFA01000032	364648	365160	-	conserved hypothetical protein	-7,923300436	8,28E-06	-5,7279	4,78E-34	-8,652241939	1,76E-09
C4B63_32g202-t42_1	PRFA01000032	361204	363429	-	cullin	-7,940069162	0,000312	-5,2946	1,69E-05	-6,591354846	8,15E-08
C4B63_32g203-t42_1	PRFA01000032	358525	360939	-	TFIIH basal transcription factor complex helicase subunit	-8,432109735	4,62E-05	-6,37493	4,32E-16	-8,081382497	4,55E-09
C4B63_32g204-t42_1	PRFA01000032	357574	358398	-	zinc-finger multi-pass transmembrane protein	-7,354151451	4,70E-05	-5,17876	4,95E-19	-7,638399883	3,63E-09
C4B63_32g214-t42_1	PRFA01000032	344653	345867	-	2-amino-3-ketobutyrate coenzyme A ligase	-0,998169744	0,000251	-0,67514	1,80E-12	-0,992644186	2,63E-05
C4B63_32g215-t42_1	PRFA01000032	343548	344264	-	Pentatricopeptide repeat-containing protein At2g31400%2C chloroplastic	-8,240176902	4,21E-06	-5,36704	3,37E-12	-6,984407438	1,40E-07
C4B63_32g226-t42_1	PRFA01000032	321634	322218	-	amastin	-8,082212184	6,39E-06	-4,84512	3,17E-58	-8,228940085	6,21E-10
C4B63_32g227-t42_1	PRFA01000032	318439	319023	-	amastin	-8,082212184	6,39E-06	-4,84512	3,17E-58	-8,228940085	6,21E-10
C4B63_32g228-t42_1	PRFA01000032	315244	315828	-	amastin	-8,082212184	6,39E-06	-4,84512	3,17E-58	-8,228940085	6,21E-10
C4B63_32g229-t42_1	PRFA01000032	312055	312639	-	amastin	1,436915961	8,95E-05	0,847203	6,61E-05	1,029827668	2,91E-06
C4B63_32g230-t42_1	PRFA01000032	308861	309445	-	amastin	1,436915961	8,95E-05	0,847203	6,61E-05	1,029827668	2,91E-06
C4B63_43g92-t42_1	PRFA01000043	345624	348758	+	trans-sialidase%2C Group VIII	1,206915896	0,000513	0,694667	0,00263	0,733946721	0,000175
C4B63_43g95-t42_1	PRFA01000043	351035	353503	+	trans-sialidase%2C Group II	1,121880855	0,000253	0,689227	2,01E-05	0,931515315	1,24E-05
C4B63_68g103-t42_1	PRFA01000068	89317	91599	-	trans-sialidase%2C Group II	1,13090159	0,000253	0,686625	9,19E-06	0,784459043	4,70E-05

C4B63_68g107-t42_1	PRFA01000068	70118	72646	-	trans-sialidase%2C Group II	1,145258589	0,000251	0,670535	6,19E-06	0,91981441	4,94E-05
C4B63_68g109-t42_1	PRFA01000068	60539	62383	-	retrotransposon hot spot protein (RHS)	0,640703385	0,000649	0,500102	3,88E-07	0,766824326	6,46E-05
C4B63_68g121-t42_1	PRFA01000068	32935	35403	-	trans-sialidase%2C Group II	1,081556418	0,000398	0,677188	1,02E-07	1,040941941	3,87E-05
C4B63_68g79-t42_1	PRFA01000068	169571	171658	-	surface protease GP63	1,843403464	0,000501	1,071483	0,000564	1,278235524	6,19E-05
C4B63_68g93-t42_1	PRFA01000068	119849	120583	-	retrotransposon hot spot (RHS) protein	1,027895273	0,000467	0,736304	7,34E-06	1,105187359	0,000166
C4B63_77g50-t42_1	PRFA01000077	101051	103819	+	trans-sialidase%2C Group IV	1,097590934	0,000256	0,654751	0,000182	0,82825675	4,24E-05
C4B63_77g52-t42_1	PRFA01000077	109229	110974	+	retrotransposon hot spot protein (RHS)	0,604922212	0,009259	0,504086	9,96E-06	0,858164781	8,65E-05
C4B63_77g60-t42_1	PRFA01000077	122003	125029	+	trans-sialidase%2C Group III	0,723739475	0,000518	0,52776	3,80E-08	0,863225856	3,20E-05
C4B63_77g67-t42_1	PRFA01000077	131426	134452	+	trans-sialidase%2C Group III	0,890571924	0,000113	0,593347	9,41E-11	0,836662033	2,37E-05
C4B63_82g114-t42_1	PRFA01000082	10099	12477	-	unspecified product	0,779724378	0,000256	0,560511	1,99E-08	0,873903799	3,08E-05
C4B63_82g75-t42_1	PRFA01000082	108396	118167	-	unspecified product	0,682114233	0,010475	0,523596	0,00039	0,948118228	0,000114
C4B63_82g81-t42_1	PRFA01000082	91424	101914	-	dispersed gene family protein 1 (DGF-1)	0,659355489	0,004872	0,502832	0,000154	0,844283626	0,000183

Table S3: Differentially expressed genes (FDR < 5%) in the Adhered analysis (KO vs WT)											
trancriptID(Dm28c2018)	chr	start	end	strand	description	Voom		Sleuth		EdgeR	
						Log2 (KO/WT)	adjP	b	qval	Log2 (KO/WT)	FDR
C4B63_107g104-t42_1	PRFA01000107	12288	13130	-	Mucin-associated surface protein (MASP)	-1,610232726	0,00722	-1,14474	0,037618932	-1,494939336	0,000418929
C4B63_107g29-t42_1	PRFA01000107	39859	40764	+	unspecified product	-1,585194225	0,003672	-1,29645	0,004996118	-1,604324179	0,000289227
C4B63_107g32-t42_1	PRFA01000107	50807	51955	+	Mucin-associated surface protein (MASP)	-1,426073949	0,001087	-0,9906	0,000262111	-1,459914376	6,41E-05
C4B63_107g93-t42_1	PRFA01000107	29245	32070	-	trans-sialidase%2C Group VI	-1,561948641	0,000274	-1,25712	7,55E-05	-1,569026015	2,10E-06
C4B63_110g7-t42_1	PRFA01000110	8575	10197	+	multidrug resistance protein E	0,597460355	0,00065	0,425455	1,12E-09	0,555846973	0,000163188
C4B63_110g9-t42_1	PRFA01000110	12686	16531	+	conserved hypothetical protein	0,545883871	2,55E-05	0,390886	7,73E-16	0,554069764	2,05E-05
C4B63_12g221-t42_1	PRFA01000012	449058	451799	-	pyruvate phosphate dikinase	-1,156252071	3,38E-06	-0,74242	8,40E-19	-1,072231742	9,80E-07
C4B63_149g40-t42_1	PRFA01000149	65397	66239	+	unspecified product	1,192290723	0,001063	0,663792	0,022647378	0,938604109	0,00014013
C4B63_149g41-t42_1	PRFA01000149	66509	67450	+	conserved hypothetical protein	1,32847265	0,000799	0,811238	0,000119167	1,133725852	1,42E-05
C4B63_151g29-t42_1	PRFA01000151	55419	56318	+	conserved hypothetical protein	1,26218067	1,26E-05	0,776727	5,02E-10	1,108655914	2,22E-08
C4B63_151g31-t42_1	PRFA01000151	59428	59940	+	conserved hypothetical protein	0,961555164	0,000269	0,638797	1,01E-11	0,845508223	4,95E-05
C4B63_151g33-t42_1	PRFA01000151	61145	63370	+	cullin	1,155511539	1,24E-05	0,725303	8,86E-11	1,031618904	8,37E-06
C4B63_151g34-t42_1	PRFA01000151	63628	66042	+	TFIIH basal transcription factor complex helicase subunit	0,935032252	1,74E-06	0,615231	3,31E-27	0,87554555	4,67E-07
C4B63_151g35-t42_1	PRFA01000151	66166	66990	+	zinc-finger multi-pass transmembrane protein	1,200994543	2,78E-06	0,78641	6,20E-28	1,142641003	6,45E-07
C4B63_151g36-t42_1	PRFA01000151	67366	68874	+	notchless homolog	1,1327554	3,92E-05	0,856464	1,02E-08	1,220549353	1,47E-07
C4B63_151g38-t42_1	PRFA01000151	49425	50972	-	unspecified product	1,188615191	1,74E-06	0,811965	7,89E-39	1,225569605	4,71E-08
C4B63_151g41-t42_1	PRFA01000151	44105	45802	-	retrotransposon hot spot (RHS) protein	1,06084477	0,000117	0,737927	1,22E-08	1,144590061	2,65E-05
C4B63_151g43-t42_1	PRFA01000151	40420	41973	-	unspecified product	1,509657709	0,002609	0,957599	6,18E-06	1,442672578	0,000192991
C4B63_151g47-t42_1	PRFA01000151	26076	28088	-	unspecified product	0,863470066	3,92E-05	0,573096	2,30E-10	0,885552876	4,70E-06
C4B63_151g48-t42_1	PRFA01000151	16457	18292	-	retrotransposon hot spot (RHS) protein	1,07786611	2,56E-06	0,687777	3,48E-24	0,981432436	1,60E-07
C4B63_151g54-t42_1	PRFA01000151	3646	14330	-	unspecified product	0,728175862	0,000117	0,457156	4,87E-10	0,703357101	1,17E-05
C4B63_16g11-t42_1	PRFA01000016	23961	24878	+	5'-AMP-activated protein kinase subunit beta	0,690367715	0,000408	0,47999	4,37E-12	0,632703265	9,64E-05
C4B63_16g114-t42_1	PRFA01000016	336905	341050	+	Target of rapamycin complex 2 subunit ste20	0,50814015	2,50E-05	0,320419	7,17E-14	0,455409751	0,000282327
C4B63_16g12-t42_1	PRFA01000016	26102	27061	+	conserved hypothetical protein	0,652769417	0,001656	0,47449	3,19E-07	0,747998643	6,51E-05
C4B63_16g121-t42_1	PRFA01000016	353204	367228	+	dynein heavy chain	0,772773082	0,00035	0,43329	0,009896834	0,594495953	0,000203988
C4B63_16g139-t42_1	PRFA01000016	396139	397056	+	palmitoyl acyltransferase 12	0,858013912	3,22E-05	0,594831	6,17E-19	0,8248597	8,85E-06
C4B63_16g140-t42_1	PRFA01000016	397584	402098	+	Paladin	0,999087916	7,88E-05	0,646553	1,22E-09	0,826395875	2,47E-06
C4B63_16g142-t42_1	PRFA01000016	405252	407780	+	trans-sialidase%2C Group II	0,622801234	0,000193	0,449985	4,32E-09	0,664465368	4,03E-05
C4B63_16g146-t42_1	PRFA01000016	413477	414994	+	Meckel syndrome type 1 protein	0,624166036	0,000742	0,438523	3,26E-08	0,671755313	0,000121275
C4B63_16g147-t42_1	PRFA01000016	417079	417951	+	Ring finger domain containing protein	0,695375534	4,92E-05	0,472648	1,13E-16	0,627664319	2,30E-06
C4B63_16g17-t42_1	PRFA01000016	34316	36349	+	acetyl-CoA synthetase	0,411745444	5,07E-05	0,297547	7,39E-12	0,409562339	0,000512958
C4B63_16g21-t42_1	PRFA01000016	43892	44434	+	conserved hypothetical protein	0,553149246	0,000116	0,3469	4,97E-07	0,528716675	0,000129484
C4B63_16g269-t42_1	PRFA01000016	257345	259813	-	conserved hypothetical protein	0,765755677	0,001626	0,464608	0,000700341	0,646705318	0,000273221
C4B63_16g27-t42_1	PRFA01000016	50144	51694	+	WW domain containing protein	0,530645459	0,000497	0,397263	6,18E-06	0,573599672	0,000176046
C4B63_16g274-t42_1	PRFA01000016	246107	249091	-	conserved hypothetical protein	0,6064827	0,00011	0,40566	5,35E-15	0,650590485	5,30E-06
C4B63_16g28-t42_1	PRFA01000016	51922	53466	+	Protein X92	0,539805022	0,000323	0,388146	1,20E-06	0,549835781	0,000342396
C4B63_16g280-t42_1	PRFA01000016	203610	205595	-	conserved hypothetical protein	0,745792428	0,000107	0,45417	1,58E-06	0,616377794	6,16E-05
C4B63_16g281-t42_1	PRFA01000016	199885	203295	-	Na/H antiporter-like protein	0,614093442	1,70E-05	0,395488	2,39E-14	0,557137015	3,96E-06
C4B63_16g284-t42_1	PRFA01000016	196229	197785	-	actin interacting protein-like protein	0,512416674	0,000255	0,34853	2,09E-10	0,527073488	0,000192143

C4B63_16g285-t42_1	PRFA01000016	194987	195751	-	conserved hypothetical protein	0,59430994	0,000556	0,442575	2,69E-06	0,637986061	0,000211879
C4B63_16g286-t42_1	PRFA01000016	192601	194694	-	Histone-lysine N-methyltransferase SMYD3	0,521568442	0,000168	0,371606	4,35E-08	0,548600795	0,000121621
C4B63_16g289-t42_1	PRFA01000016	189303	190295	-	conserved hypothetical protein	0,525929574	0,00017	0,351444	7,64E-07	0,53731387	0,000104203
C4B63_16g3-t42_1	PRFA01000016	7473	7979	+	aldose 1-epimerase-like protein	0,476995537	0,000189	0,34218	2,31E-07	0,490493329	0,000393083
C4B63_16g311-t42_1	PRFA01000016	128505	135308	-	unspecified product	0,68809831	3,09E-05	0,417014	1,17E-07	0,60359091	4,58E-05
C4B63_16g329-t42_1	PRFA01000016	95141	97729	-	conserved hypothetical protein	0,532271002	1,35E-05	0,36509	9,20E-15	0,538410572	2,29E-05
C4B63_16g33-t42_1	PRFA01000016	65468	67915	+	trans-sialidase%2C Group VIII	0,830731534	1,35E-05	0,531258	9,43E-18	0,731305177	3,91E-06
C4B63_16g330-t42_1	PRFA01000016	92335	94587	-	kinesin-C	0,783727687	5,86E-06	0,493406	1,48E-17	0,714524753	3,71E-07
C4B63_16g331-t42_1	PRFA01000016	87043	90159	-	ubiquitin-activating enzyme E1	0,666760155	3,04E-05	0,448012	1,64E-21	0,670902292	1,37E-05
C4B63_16g332-t42_1	PRFA01000016	85191	86270	-	metallo-beta-lactamase-like protein	0,877603262	2,33E-05	0,589805	1,12E-09	0,845995791	7,27E-06
C4B63_16g35-t42_1	PRFA01000016	77694	78380	+	unspecified product	0,824463624	0,000356	0,520218	6,95E-09	0,714105485	0,000275416
C4B63_16g36-t42_1	PRFA01000016	78767	79936	+	unspecified product	0,717769009	0,001626	0,474696	9,45E-06	0,576355665	0,00032572
C4B63_16g37-t42_1	PRFA01000016	80172	80717	+	unspecified product	0,850428365	0,000282	0,561575	1,20E-06	0,781238158	0,00017512
C4B63_16g6-t42_1	PRFA01000016	10883	14323	+	permease-like protein	0,577228922	9,89E-05	0,368405	6,16E-10	0,489579869	0,000529046
C4B63_16g742c-t42_1	PRFA01000016	416202	416639	+	conserved hypothetical protein	0,61176375	0,000395	0,424352	2,20E-06	0,637052587	0,000125815
C4B63_16g86-t42_1	PRFA01000016	274837	276630	+	mannosyl-oligosaccharide 1%2C2-alpha-mannosidase IB	0,538785284	7,75E-06	0,364168	2,45E-11	0,530112392	1,87E-05
C4B63_16g93-t42_1	PRFA01000016	286038	288017	+	Uncharacterized protein in mobD 3'region	0,501960814	4,28E-05	0,311746	4,12E-12	0,467582374	5,01E-05
C4B63_173g13-t42_1	PRFA01000173	21330	21628	+	unspecified product	-2,011139187	0,000509	-1,39993	0,001738038	-1,9162605	0,000155863
C4B63_178g34-t42_1	PRFA01000178	54022	54489	-	unspecified product	1,086336766	0,001617	0,608113	0,022194323	0,865512885	3,96E-06
C4B63_178g41-t42_1	PRFA01000178	33472	43860	-	dispersed gene family protein 1 (DGF-1)	0,62701516	7,43E-05	0,383628	3,26E-07	0,609932606	3,97E-06
C4B63_178g46-t42_1	PRFA01000178	10396	12861	-	conserved hypothetical protein	0,517693054	2,15E-05	0,375334	1,54E-09	0,525325658	3,25E-05
C4B63_178g47-t42_1	PRFA01000178	233	8216	-	unspecified product	0,584016941	0,002559	0,352585	0,038300806	0,618005875	4,06E-05
C4B63_17g327-t42_1	PRFA01000017	10819	11910	-	retrotransposon hot spot (RHS) protein	1,001618185	0,000191	0,56586	0,001497792	0,802373741	0,000425846
C4B63_17g328-t42_1	PRFA01000017	9996	10877	-	retrotransposon hot spot protein (RHS)	1,142121306	0,001024	0,67149	0,000102844	0,968038447	0,00024587
C4B63_182g13-t42_1	PRFA01000182	25486	35991	+	unspecified product	0,576137459	0,000121	0,381603	1,36E-10	0,57179609	0,000108493
C4B63_182g24-t42_1	PRFA01000182	43446	54115	+	unspecified product	0,573890652	1,60E-05	0,357508	1,77E-12	0,536482547	6,07E-06
C4B63_182g5-t42_1	PRFA01000182	8170	18678	+	dispersed gene family protein 1 (DGF-1)	0,607512415	3,44E-06	0,419844	8,54E-23	0,61423432	2,68E-06
C4B63_1g629-t42_1	PRFA01000001	1437769	1438737	+	Mucin-associated surface protein (MASP)	-0,96625444	0,022645	-0,86355	0,006362457	-1,274599093	0,000477096
C4B63_1g806-t42_1	PRFA01000001	1424024	1426450	-	trans-sialidase%2C Group V	-1,716796932	0,000387	-1,1869	4,34E-11	-1,596890176	5,94E-05
C4B63_1g813-t42_1	PRFA01000001	1410713	1412071	-	unspecified product	-1,822632304	0,00296	-1,23105	0,011023518	-1,950579302	0,000210792
C4B63_1g819-t42_1	PRFA01000001	1400040	1402877	-	trans-sialidase%2C Group VI	-1,192740138	2,68E-05	-0,87653	1,40E-17	-1,225005248	4,54E-06
C4B63_203g4-t42_1	PRFA01000203	4632	5258	+	unspecified product	0,963122668	0,000245	0,563493	5,32E-05	0,849550891	0,000349119
C4B63_203g5-t42_1	PRFA01000203	6569	7513	+	conserved hypothetical protein	0,927110934	0,001081	0,496408	0,047700408	0,717171542	4,03E-05
C4B63_216g7-t42_1	PRFA01000216	25069	26922	+	protein associated with differentiation 4	0,734008567	9,62E-05	0,433653	6,56E-06	0,632773613	2,20E-05
C4B63_219g47-t42_1	PRFA01000219	20336	22567	-	multidrug resistance-associated protein(fragment)	0,519479202	2,53E-05	0,372366	2,99E-18	0,5682851	3,75E-06
C4B63_219g48-t42_1	PRFA01000219	17822	20293	-	multidrug resistance protein E	0,515813907	1,24E-05	0,375116	3,46E-13	0,547208952	6,04E-06
C4B63_220g11-t42_1	PRFA01000220	39064	40512	+	Beta propeller protein 1	0,546434137	0,00017	0,383851	1,63E-10	0,533719041	0,000256575
C4B63_220g13-t42_1	PRFA01000220	41929	42705	+	cyclophilin	0,567800494	4,67E-05	0,39318	1,31E-09	0,562556533	7,37E-05
C4B63_220g9-t42_1	PRFA01000220	36218	37525	+	conserved hypothetical protein	0,693318735	2,48E-05	0,478163	6,97E-15	0,673709646	1,26E-05
C4B63_222g26-t42_1	PRFA01000222	38126	38899	-	UDP-Gal or UDP-GlcNAc-dependent glycosyltransferase	-1,837343605	0,032012	-1,46673	0,007394946	-1,597748114	0,000190891
C4B63_225g26-t42_1	PRFA01000225	35296	37248	-	multidrug resistance protein E	0,439907174	2,86E-05	0,31772	1,65E-13	0,438451483	9,52E-05
C4B63_225g35-t42_1	PRFA01000225	10762	21332	-	unspecified product	0,455303834	0,000509	0,30295	3,64E-09	0,48116117	0,000209799
C4B63_23g21-t42_1	PRFA01000023	53692	55149	+	conserved hypothetical protein	0,769573446	0,000188	0,506815	1,75E-09	0,673566127	8,23E-05

C4B63_23g4-t42_1	PRFA01000023	8277	9029	+	GPR1/FUN34/yaaH family	-0,518900713	0,000245	-0,37625	2,31E-12	-0,547464498	0,00021163
C4B63_241g14-t42_1	PRFA01000241	16601	17215	+	conserved hypothetical protein	0,538520447	0,00032	0,41293	2,59E-13	0,546630815	7,29E-05
C4B63_241g18-t42_1	PRFA01000241	27546	29669	+	conserved hypothetical protein	0,916493573	0,000483	0,511517	0,013205715	0,738948144	3,93E-05
C4B63_241g23-t42_1	PRFA01000241	36937	37617	+	unspecified product	0,89959702	0,004763	0,507959	0,037618932	0,74999138	0,000112896
C4B63_241g24-t42_1	PRFA01000241	38531	39475	+	conserved hypothetical protein	0,933755678	0,000117	0,540654	0,00022221	0,793097423	7,60E-05
C4B63_241g7-t42_1	PRFA01000241	8600	9682	+	Leucine-rich repeat-containing protein 34	0,683513069	0,000149	0,47118	3,86E-11	0,665609309	6,95E-05
C4B63_256g19-t42_1	PRFA01000256	12069	22508	-	dispersed gene family protein 1 (DGF-1)	1,240147121	3,70E-05	0,823485	1,10E-17	1,152093515	2,29E-06
C4B63_26g261-t42_1	PRFA01000026	224353	227355	-	glutamate dehydrogenase	0,499941495	0,000143	0,318029	1,66E-10	0,441923302	0,000108369
C4B63_279g12-t42_1	PRFA01000279	13181	13789	-	beta tubulin	-1,189441804	0,018206	-0,97215	0,027814114	-1,017381636	0,000148419
C4B63_31g166-t42_1	PRFA01000031	388657	390453	-	histone deacetylase 4	-8,066987364	2,68E-06	-5,19432	1,82E-20	-7,652296463	4,72E-11
C4B63_31g167-t42_1	PRFA01000031	387153	388403	-	conserved hypothetical protein	-1,208886008	0,000264	-0,73712	3,35E-07	-1,071394635	0,000143976
C4B63_320g20-t42_1	PRFA01000320	2089	2976	-	histone deacetylase	1,605899383	0,002716	1,28109	0,000559553	1,839843823	0,000390892
C4B63_328g10-t42_1	PRFA01000328	10231	14976	+	multidrug resistance protein E	0,646965503	2,78E-06	0,450064	2,77E-24	0,631317176	1,31E-06
C4B63_328g12-t42_1	PRFA01000328	17465	21298	+	conserved hypothetical protein	0,543845309	3,82E-05	0,351294	2,33E-14	0,522081324	1,12E-05
C4B63_328g8-t42_1	PRFA01000328	6842	7456	+	conserved hypothetical protein	0,538520447	0,00032	0,41293	2,59E-13	0,546630815	7,29E-05
C4B63_32g138-t42_1	PRFA01000032	372866	375253	+	unspecified product	-4,181328713	0,000323	-3,14879	1,35E-05	-4,233890904	7,58E-06
C4B63_32g139-t42_1	PRFA01000032	377181	380183	+	trans-sialidase%2C Group VI	-1,369196826	3,00E-05	-0,93076	1,15E-18	-1,354109334	3,47E-06
C4B63_32g171-t42_1	PRFA01000032	426951	427727	+	unspecified product	-5,902385676	7,43E-05	-4,36232	5,66E-12	-5,650477714	1,57E-06
C4B63_32g172-t42_1	PRFA01000032	428939	431188	+	trans-sialidase%2C Group II	-0,806069241	0,001106	-0,61906	2,41E-08	-0,907993921	9,42E-05
C4B63_32g177-t42_1	PRFA01000032	417391	419745	-	unspecified product	-5,145434109	1,30E-05	-3,83595	1,98E-11	-5,373497015	1,24E-07
C4B63_32g181-t42_1	PRFA01000032	404401	414789	-	dispersed gene family protein 1 (DGF-1)	-7,742015666	2,78E-06	-5,33494	1,94E-31	-7,661679817	1,36E-10
C4B63_32g185-t42_1	PRFA01000032	395454	396209	-	unspecified product	-3,325575621	0,000227	-2,57795	8,13E-06	-3,446843889	2,60E-05
C4B63_32g186-t42_1	PRFA01000032	393324	394817	-	Mucin-associated surface protein (MASP)	-6,33473259	1,74E-06	-4,54371	2,49E-27	-7,218610854	6,49E-08
C4B63_32g187-t42_1	PRFA01000032	387776	390559	-	trans-sialidase%2C Group V	-4,061130018	0,001024	-3,01635	0,000165299	-3,974695022	9,20E-05
C4B63_32g189-t42_1	PRFA01000032	384386	385171	-	unspecified product	-6,462756698	4,22E-07	-4,65012	1,90E-25	-8,74304851	9,95E-06
C4B63_32g198-t42_1	PRFA01000032	368205	369104	-	conserved hypothetical protein	-8,228069885	2,78E-06	-5,3807	5,17E-24	-7,829797073	5,43E-10
C4B63_32g200-t42_1	PRFA01000032	364648	365160	-	conserved hypothetical protein	-7,900824139	1,23E-06	-5,62879	6,95E-35	-8,279138513	1,07E-09
C4B63_32g202-t42_1	PRFA01000032	361204	363429	-	cullin	-8,591509716	7,14E-05	-6,02697	2,76E-10	-7,289115198	2,90E-08
C4B63_32g203-t42_1	PRFA01000032	358525	360939	-	TFIIH basal transcription factor complex helicase subunit	-8,724840002	7,14E-05	-6,30124	1,92E-10	-7,202741247	1,19E-08
C4B63_32g204-t42_1	PRFA01000032	357574	358398	-	zinc-finger multi-pass transmembrane protein	-9,012639635	2,39E-08	-6,34094	2,92E-178	-11,20515386	1,74E-08
C4B63_32g205-t42_1	PRFA01000032	355690	357255	-	notchless homolog	-0,998117675	0,000111	-0,6889	2,07E-11	-0,997768223	1,58E-05
C4B63_32g211-t42_1	PRFA01000032	349223	349717	-	60S ribosomal protein L12	0,877170542	6,15E-07	0,620596	2,52E-51	0,902683331	6,60E-09
C4B63_32g212-t42_1	PRFA01000032	346945	347817	-	Pentatricopeptide repeat-containing protein At2g31400%2C chloroplastic	0,632238514	4,92E-05	0,433159	1,01E-11	0,58072818	1,59E-05
C4B63_32g214-t42_1	PRFA01000032	344653	345867	-	2-amino-3-ketobutyrate coenzyme A ligase	-0,95690629	1,36E-05	-0,64858	7,04E-14	-0,889312862	1,46E-06
C4B63_32g215-t42_1	PRFA01000032	343548	344264	-	Pentatricopeptide repeat-containing protein At2g31400%2C chloroplastic	-8,77575985	4,06E-07	-6,13357	6,68E-137	-10,71441743	8,25E-08
C4B63_32g226-t42_1	PRFA01000032	321634	322218	-	amastin	-7,481092985	4,06E-07	-4,82021	4,09E-43	-8,462641985	1,31E-09
C4B63_32g227-t42_1	PRFA01000032	318439	319023	-	amastin	-7,481092985	4,06E-07	-4,82021	4,09E-43	-8,462641985	1,31E-09
C4B63_32g228-t42_1	PRFA01000032	315244	315828	-	amastin	-7,481092985	4,06E-07	-4,82021	4,09E-43	-8,462641985	1,31E-09
C4B63_32g229-t42_1	PRFA01000032	312055	312639	-	amastin	1,206540747	2,78E-06	0,785277	2,30E-34	1,119238895	1,41E-07
C4B63_32g230-t42_1	PRFA01000032	308861	309445	-	amastin	1,206540747	2,78E-06	0,785277	2,30E-34	1,119238895	1,41E-07
C4B63_340nc2-t42_1	PRFA01000340	22928	22999	+	tRNA-Gln	-2,774995183	0,005749	-1,84033	0,000447626	-2,702956886	0,000115
C4B63_341g11-t42_1	PRFA01000341	21156	22202	-	lipase	1,055321054	6,42E-05	0,701619	5,52E-10	1,065591324	1,18E-05
C4B63_341g12-t42_1	PRFA01000341	19737	20783	-	lipase	0,834223751	0,000117	0,565956	6,80E-07	0,851757003	1,60E-05

C4B63_34g281-t42_1	PRFA01000034	217455	219470	-	conserved hypothetical protein	0,664806689	0,001087	0,380175	0,004493643	0,524151301	0,000144351
C4B63_43g92-t42_1	PRFA01000043	345624	348758	+	trans-sialidase%2C Group VIII	0,659227175	0,000116	0,449478	2,09E-09	0,653690659	4,88E-05
C4B63_43g95-t42_1	PRFA01000043	351035	353503	+	trans-sialidase%2C Group II	0,655180399	0,001121	0,532279	1,41E-06	0,712622041	0,000515403
C4B63_55g99-t42_1	PRFA01000055	242751	243284	+	conserved hypothetical protein	-7,025216239	1,74E-06	-4,88384	9,12E-05	-9,417302703	1,83E-06
C4B63_68g103-t42_1	PRFA01000068	89317	91599	-	trans-sialidase%2C Group II	0,880578036	1,30E-05	0,62096	9,75E-37	0,889987109	8,79E-07
C4B63_68g107-t42_1	PRFA01000068	70118	72646	-	trans-sialidase%2C Group II	0,616571786	0,000434	0,452805	3,26E-06	0,629032703	0,000178937
C4B63_68g109-t42_1	PRFA01000068	60539	62383	-	retrotransposon hot spot protein (RHS)	0,675637126	2,86E-05	0,439431	6,34E-15	0,612246348	5,80E-05
C4B63_68g110-t42_1	PRFA01000068	59136	60068	-	retrotransposon hot spot (RHS) protein	0,478653072	0,000112	0,335153	6,78E-08	0,483882681	0,000235916
C4B63_68g111-t42_1	PRFA01000068	53256	55460	-	trans-sialidase%2C Group II	0,489966953	0,00307	0,412944	1,82E-05	0,568752815	0,000272231
C4B63_68g120-t42_1	PRFA01000068	39113	41554	-	retrotransposon hot spot (RHS) protein	0,567329043	6,23E-06	0,404557	7,96E-18	0,581493462	4,68E-06
C4B63_68g70-t42_1	PRFA01000068	189339	191840	-	neutral sphingomyelinase activation associated factor-like protein	0,614055415	0,000355	0,351661	0,001416522	0,54653795	0,000159929
C4B63_68g74-t42_1	PRFA01000068	177160	180297	-	trans-sialidase%2C Group VIII	0,689055514	9,62E-05	0,522811	3,98E-12	0,680935196	7,33E-06
C4B63_68g77-t42_1	PRFA01000068	172187	173371	-	Mucin-associated surface protein (MASP)	0,663405037	0,000168	0,459862	8,91E-11	0,605736508	0,000103011
C4B63_68g79-t42_1	PRFA01000068	169571	171658	-	surface protease GP63	1,292621437	0,001377	0,894516	0,008130857	1,185399089	0,000296338
C4B63_68g83-t42_1	PRFA01000068	151070	161440	-	dispersed gene family protein 1 (DGF-1)	0,712681288	2,58E-05	0,489682	3,23E-24	0,73832461	3,50E-06
C4B63_68g91-t42_1	PRFA01000068	125009	128143	-	trans-sialidase%2C Group VIII	0,650078346	0,000117	0,415337	1,39E-09	0,632440761	3,56E-05
C4B63_68g98-t42_1	PRFA01000068	106031	107575	-	unspecified product	0,557418322	0,01311	0,448517	0,0003797	0,726624219	0,000480617
C4B63_77g46-t42_1	PRFA01000077	93413	94468	+	NADP-dependent alcohol hydrogenase	0,734261396	0,000387	0,445941	0,003898723	0,693889807	0,000318177
C4B63_77g53-t42_1	PRFA01000077	112536	115550	+	trans-sialidase%2C Group III	0,546206068	2,38E-05	0,385774	8,74E-17	0,53471057	1,80E-05
C4B63_77g60-t42_1	PRFA01000077	122003	125029	+	trans-sialidase%2C Group III	0,702437539	2,78E-06	0,476009	1,13E-24	0,718059541	2,05E-07
C4B63_77g67-t42_1	PRFA01000077	131426	134452	+	trans-sialidase%2C Group III	0,717024949	1,23E-06	0,487526	3,34E-26	0,708813777	3,03E-07
C4B63_77g74-t42_1	PRFA01000077	141784	142839	+	NADP-dependent alcohol hydrogenase	0,716624074	2,33E-05	0,442293	7,21E-11	0,66452226	2,97E-05
C4B63_77g75-t42_1	PRFA01000077	143718	147230	+	ABC transporter	0,636213646	2,78E-06	0,450086	5,66E-27	0,63217609	1,72E-06
C4B63_77g77-t42_1	PRFA01000077	148417	151488	+	conserved hypothetical protein	0,751394862	7,14E-05	0,519894	4,17E-20	0,703468165	1,33E-05
C4B63_77g78-t42_1	PRFA01000077	151547	152860	+	conserved hypothetical protein	0,631025032	0,000106	0,416265	1,23E-07	0,629586535	7,65E-05
C4B63_82g107-t42_1	PRFA01000082	27776	28831	-	NADP-dependent alcohol hydrogenase	0,815206704	0,000183	0,482552	1,11E-05	0,733071531	2,80E-05
C4B63_82g110-t42_1	PRFA01000082	23902	24699	-	beta galactofuranosyl glycosyltransferase	0,745704447	0,000727	0,54349	0,002641798	0,806047423	0,000100297
C4B63_82g111-t42_1	PRFA01000082	18424	21192	-	trans-sialidase%2C Group IV	0,673462188	0,002791	0,448605	1,19E-06	0,687289866	0,000510279
C4B63_82g114-t42_1	PRFA01000082	10099	12477	-	unspecified product	0,612824724	0,000233	0,428206	2,78E-10	0,531829154	9,59E-05
C4B63_82g448c-t42_1	PRFA01000082	36387	36749	-	conserved hypothetical protein	0,667928729	3,00E-05	0,440375	1,67E-15	0,663006818	1,58E-05
C4B63_82g70-t42_1	PRFA01000082	126035	136534	-	dispersed gene family protein 1 (DGF-1)	0,663801485	4,52E-06	0,430633	1,60E-24	0,65019052	2,04E-07
C4B63_82g75-t42_1	PRFA01000082	108396	118167	-	unspecified product	0,659194329	1,31E-05	0,451816	3,41E-22	0,689845088	1,34E-06
C4B63_82g79-t42_1	PRFA01000082	106332	107669	-	tryptophanyl-tRNA synthetase	0,422486555	0,007818	0,365764	0,001776224	0,517149079	0,000273846
C4B63_82g81-t42_1	PRFA01000082	91424	101914	-	dispersed gene family protein 1 (DGF-1)	0,649368701	1,51E-05	0,436918	4,93E-23	0,663506846	1,26E-06
C4B63_82g85-t42_1	PRFA01000082	73787	84286	-	dispersed gene family protein 1 (DGF-1)	0,836130325	2,01E-06	0,535711	4,30E-24	0,785360146	2,28E-07
C4B63_82g89-t42_1	PRFA01000082	55235	65488	-	dispersed gene family protein 1 (DGF-1)	1,102463065	1,13E-05	0,666831	1,76E-08	0,969243255	7,17E-07
C4B63_82g94-t42_1	PRFA01000082	43064	46264	-	conserved hypothetical protein	0,725901896	6,85E-05	0,512935	7,95E-16	0,693639221	1,13E-05
C4B63_82g96-t42_1	PRFA01000082	41644	41979	-	(H+)-ATPase G subunit	0,575543317	0,000149	0,403686	5,26E-12	0,561922422	0,000121807
C4B63_82g97-t42_1	PRFA01000082	39399	40283	-	conserved hypothetical protein	0,621444643	9,14E-05	0,423589	6,82E-12	0,612710116	6,98E-05
C4B63_82g98-t42_1	PRFA01000082	37040	38827	-	glycosyl transferase	0,446939726	0,000191	0,359351	1,12E-09	0,500762309	0,000118801
C4B63_85g44-t42_1	PRFA01000085	109890	110498	-	ribosomal protein S7	-0,646201407	1,25E-05	-0,42298	5,25E-19	-0,625757533	1,22E-06
C4B63_85g45-t42_1	PRFA01000085	109007	109615	-	ribosomal protein S7	0,807600643	2,78E-06	0,568936	1,89E-29	0,830450622	1,39E-07

Table S4: Differentially expressed genes (probability 0.95) in the Metacyclic Trypomastigote analysis (KO vs WT)							
trancriptID(Dm28c2018)	chr	start	end	strand	description	NOISeq	
						M	Probability
C4B63_101g4-t42_1	PRFA01000101	9162	9665	+	Note=2.6.1.5 (Tyrosine transaminase)	0,9060234	0,963231822
C4B63_102g43-t42_1	PRFA01000102	80712	81377	-	Note=2.4.2.8 (Hypoxanthine phosphoribosyltransferase)	1,2307797	0,980341124
C4B63_10g100-t42_1	PRFA01000010	313181	314620	+	LETM1 and EF-hand domain-containing protein 1	-0,9203743	0,963515109
C4B63_10g103-t42_1	PRFA01000010	318996	321254	+	Hercynine oxygenase	-0,9879533	0,970520538
C4B63_10g119-t42_1	PRFA01000010	351486	352610	+	60S ribosomal protein L4	1,0967542	0,978310907
C4B63_10g120-t42_1	PRFA01000010	353291	354415	+	60S ribosomal protein L4	1,0967542	0,978310907
C4B63_10g121-t42_1	PRFA01000010	355097	355630	+	60S ribosomal protein L4	0,8108114	0,951404627
C4B63_10g122-t42_1	PRFA01000010	355675	356217	+	60S ribosomal protein L4	1,2087754	0,984448772
C4B63_10g123-t42_1	PRFA01000010	356899	357432	+	60S ribosomal protein L4	0,8108114	0,951404627
C4B63_10g124-t42_1	PRFA01000010	357477	358019	+	60S ribosomal protein L4	1,2087754	0,984448772
C4B63_10g153-t42_1	PRFA01000010	407659	408519	+	RNA-binding protein	-0,9060862	0,962588527
C4B63_110g23-t42_1	PRFA01000110	38499	38987	+	conserved hypothetical protein	1,5482619	0,981126062
C4B63_110g27-t42_1	PRFA01000110	44184	45296	+	Leucine-rich repeat-containing protein 34	1,4414317	0,989134797
C4B63_110g29-t42_1	PRFA01000110	45567	47075	+	protein kinase A regulatory subunit	0,9508419	0,957477573
C4B63_110g7-t42_1	PRFA01000110	8575	10197	+	multidrug resistance protein E	1,0703337	0,950997403
C4B63_111g124c-t42_1	PRFA01000111	62806	63174	+	casein kinase%2C delta isoform	1,1901011	0,980588999
C4B63_111g154c-t42_1	PRFA01000111	98854	99222	+	casein kinase%2C delta isoform	1,1901011	0,980588999
C4B63_112g31-t42_1	PRFA01000112	63154	64821	+	Probable L-cysteine desulfhydrase%2C chloroplastic	-1,051766	0,961768178
C4B63_116g2-t42_1	PRFA01000116	1095	2102	+	Note=7.1.2.1 (P-type H(+)-exporting transporter)	-0,8525308	0,956149669
C4B63_119g26-t42_1	PRFA01000119	55814	57517	+	unspecified product	-1,0513504	0,975838055
C4B63_11g29-t42_1	PRFA01000011	98716	99552	+	Note=4.1.2.52 (4-hydroxy-2-oxoheptanedioate aldolase)	1,075922	0,968755902
C4B63_128g242c-t42_1	PRFA01000128	46181	46453	-	dynein light chain	1,2245013	0,984761568
C4B63_128g52-t42_1	PRFA01000128	37611	38069	-	conserved hypothetical protein	1,6381682	0,994599858
C4B63_13g140-t42_1	PRFA01000013	494354	494695	+	dynein light chain	1,1248141	0,979621105
C4B63_13g295-t42_1	PRFA01000013	232199	234682	-	pumilio/PUF RNA binding protein 6	-0,9274217	0,964376771
C4B63_149g19-t42_1	PRFA01000149	36453	37133	+	Note=1.11.1.15 (Peroxiredoxin)	0,868779	0,958970727
C4B63_149g38-t42_1	PRFA01000149	60317	62260	+	unspecified product	1,3664778	0,981615911
C4B63_149g40-t42_1	PRFA01000149	65397	66239	+	unspecified product	1,3069404	0,986602927
C4B63_149g41-t42_1	PRFA01000149	66509	67450	+	conserved hypothetical protein	1,7385008	0,99398017
C4B63_14g126-t42_1	PRFA01000014	284930	287284	+	trans-sialidase%2C Group I	-1,0109218	0,970042493
C4B63_14g127-t42_1	PRFA01000014	288406	288857	+	unspecified product	-3,736083	0,966802408

C4B63_14g128-t42_1	PRFA01000014	289585	291942	+	trans-sialidase%2C Group I	-1,1116811	0,979190274
C4B63_14g129-t42_1	PRFA01000014	295105	296595	+	unspecified product	-1,0312964	0,972615675
C4B63_14g140-t42_1	PRFA01000014	312873	315230	+	trans-sialidase%2C Group I	-1,1284751	0,979981114
C4B63_14g3-t42_1	PRFA01000014	2880	4088	+	hexose transporter	1,0930785	0,974592776
C4B63_14g95-t42_1	PRFA01000014	212165	213577	+	conserved hypothetical protein	-1,0577228	0,975430831
C4B63_150g40-t42_1	PRFA01000150	11932	13905	-	Note=3.4.24.- (Metalloendopeptidases.)	-1,1078084	0,977626298
C4B63_161g32-t42_1	PRFA01000161	25670	27112	-	amino acid transporter	0,8280927	0,953641407
C4B63_16g10-t42_1	PRFA01000016	22484	23194	+	conserved hypothetical protein	1,9611711	0,991914542
C4B63_16g107-t42_1	PRFA01000016	318904	320298	+	S-adenosyl-methyltransferase mraW-like protein	1,0283516	0,968088999
C4B63_16g12-t42_1	PRFA01000016	26102	27061	+	conserved hypothetical protein	1,2982574	0,977260387
C4B63_16g154-t42_1	PRFA01000016	425396	426166	+	exosome component CSL4	1,5284567	0,987358357
C4B63_16g165-t42_1	PRFA01000016	443011	444369	+	conserved hypothetical protein	1,3392548	0,986986544
C4B63_16g176-t42_1	PRFA01000016	462092	464593	+	neutral sphingomyelinase activation associated factor-like protein	-0,9045441	0,960103872
C4B63_16g280-t42_1	PRFA01000016	203610	205595	-	conserved hypothetical protein	0,9715218	0,966353872
C4B63_16g291-t42_1	PRFA01000016	184034	185383	-	conserved hypothetical protein	1,0182204	0,968443107
C4B63_16g311-t42_1	PRFA01000016	128505	135308	-	unspecified product	1,007265	0,972043201
C4B63_16g312-t42_1	PRFA01000016	127472	128017	-	unspecified product	1,0114005	0,972155335
C4B63_16g332-t42_1	PRFA01000016	85191	86270	-	metallo-beta-lactamase-like protein	1,1705665	0,973300283
C4B63_16g691c-t42_1	PRFA01000016	389285	389683	+	conserved hypothetical protein	1,1673375	0,964187913
C4B63_16g742c-t42_1	PRFA01000016	416202	416639	+	conserved hypothetical protein	1,504514	0,987175401
C4B63_16g8-t42_1	PRFA01000016	17000	17896	+	conserved hypothetical protein	1,231797	0,982223796
C4B63_16g97-t42_1	PRFA01000016	301450	302139	+	conserved hypothetical protein	1,0254765	0,974268178
C4B63_16g99-t42_1	PRFA01000016	305353	306192	+	cytosolic leucyl aminopeptidase	1,3062806	0,975979698
C4B63_175g48c-t42_1	PRFA01000175	32412	32738	+	conserved hypothetical protein	1,4640892	0,991619452
C4B63_178g41-t42_1	PRFA01000178	33472	43860	-	dispersed gene family protein 1 (DGF-1)	1,2993486	0,973335694
C4B63_178g42-t42_1	PRFA01000178	31202	31828	-	Note=6.1.1.2 (Tryptophan--tRNA ligase)	2,2512802	0,98427762
C4B63_178g44-t42_1	PRFA01000178	15484	25872	-	dispersed gene family protein 1 (DGF-1)	1,203742	0,957636922
C4B63_178g45-t42_1	PRFA01000178	13110	14165	-	Note=6.1.1.2 (Tryptophan--tRNA ligase)	1,9983786	0,995048395
C4B63_178g46-t42_1	PRFA01000178	10396	12861	-	conserved hypothetical protein	1,7202123	0,98457271
C4B63_17g119-t42_1	PRFA01000017	259881	261545	+	Phosphatidylinositol/phosphatidylcholine transfer protein SFH14	-0,9006161	0,962057365
C4B63_17g216-t42_1	PRFA01000017	458258	459217	-	DnaJ homolog	1,267979	0,981840179
C4B63_17g24-t42_1	PRFA01000017	68635	69768	+	LEM3 (ligand-effect modulator 3) family / CDC50 family	-1,0311075	0,974551464
C4B63_180g6-t42_1	PRFA01000180	8959	11220	+	trans-sialidase%2C Group II	-0,8589802	0,95786119
C4B63_18g1130c-t42_1	PRFA01000018	360138	360548	-	FUN14 family	1,2774817	0,986679651
C4B63_18g202-t42_1	PRFA01000018	440557	441789	-	conserved hypothetical protein	-0,8719223	0,957867092

C4B63_19g77-t42_1	PRFA01000019	392802	393923	+	Secretory carrier-associated membrane protein (SCAMP)	-0,9809963	0,969464117
C4B63_19g86-t42_1	PRFA01000019	416594	417529	+	CYC2-like cyclin 6	-1,1505502	0,981403447
C4B63_203g16-t42_1	PRFA01000203	44260	44874	+	Note=6.1.1.2 (Tryptophan--tRNA ligase)	1,5803907	0,956869688
C4B63_219g35-t42_1	PRFA01000219	36652	37680	-	Note=6.1.1.2 (Tryptophan--tRNA ligase)	1,2450085	0,968401794
C4B63_219g40-t42_1	PRFA01000219	28151	29263	-	Leucine-rich repeat-containing protein	1,4563562	0,972975685
C4B63_219g42-t42_1	PRFA01000219	26362	27870	-	protein kinase A regulatory subunit	1,3004258	0,973300283
C4B63_219g44-t42_1	PRFA01000219	24426	25118	-	conserved hypothetical protein	1,0497089	0,97154745
C4B63_219g45-t42_1	PRFA01000219	23596	24177	-	conserved hypothetical protein	0,9355692	0,965934844
C4B63_220g14-t42_1	PRFA01000220	43025	43594	+	conserved hypothetical protein	1,0699308	0,972420916
C4B63_225g42-t42_1	PRFA01000225	3283	3780	-	vacuolar ATP synthase	1,402981	0,975796742
C4B63_22g32-t42_1	PRFA01000022	108340	109407	+	conserved hypothetical protein	1,1841266	0,979172568
C4B63_22g773c-t42_1	PRFA01000022	452038	452433	+	small myristoylated protein-1	1,2489226	0,985782578
C4B63_236g12-t42_1	PRFA01000236	12525	13898	+	Note=6.1.1.2 (Tryptophan--tRNA ligase)	1,3535105	0,969676582
C4B63_236g16-t42_1	PRFA01000236	21250	22362	+	Leucine-rich repeat-containing protein 34	1,5652838	0,988143296
C4B63_23g130-t42_1	PRFA01000023	439116	440813	+	Note=4.2.1.2 (Fumarate hydratase)	-0,851584	0,957076251
C4B63_23g189-t42_1	PRFA01000023	301541	302656	-	Uncharacterized protein C2F7.02c	-0,9616913	0,960581917
C4B63_241g13-t42_1	PRFA01000241	15255	15782	+	conserved hypothetical protein	1,8239525	0,985817989
C4B63_241g14-t42_1	PRFA01000241	16601	17215	+	conserved hypothetical protein	1,1975482	0,980671624
C4B63_241g23-t42_1	PRFA01000241	36937	37617	+	unspecified product	0,9912592	0,968708687
C4B63_241g5-t42_1	PRFA01000241	6130	6627	+	vacuolar ATP synthase	1,402981	0,975796742
C4B63_241g7-t42_1	PRFA01000241	8600	9682	+	Leucine-rich repeat-containing protein 34	2,1556937	0,995213645
C4B63_241g9-t42_1	PRFA01000241	9992	11500	+	protein kinase A regulatory subunit	2,1984109	0,992457507
C4B63_247g13-t42_1	PRFA01000247	36678	37250	-	conserved hypothetical protein	0,9508588	0,959395656
C4B63_24g328-t42_1	PRFA01000024	99777	102011	-	trans-sialidase%2C Group II	-0,9783287	0,969529037
C4B63_259g10-t42_1	PRFA01000259	31193	32041	-	Note=1.3.5.1 (Succinate dehydrogenase (quinone))	1,0334165	0,9723678
C4B63_259g12-t42_1	PRFA01000259	28751	29947	-	conserved hypothetical protein	1,4457098	0,965622049
C4B63_25g112-t42_1	PRFA01000025	409738	413061	+	kinesin	-1,1106247	0,975112134
C4B63_26g257-t42_1	PRFA01000026	231502	232101	-	Note=1.11.1.15 (Peroxiredoxin)	1,2735457	0,985776676
C4B63_26g275-t42_1	PRFA01000026	203131	205626	-	protein phosphatase 2A regulatory subunit	-1,0315389	0,971340888
C4B63_26g278-t42_1	PRFA01000026	197832	198905	-	Note=2.7.3.2 (Creatine kinase)	1,1338635	0,979579792
C4B63_26g282-t42_1	PRFA01000026	189620	190693	-	Note=2.7.3.2 (Creatine kinase)	1,1338635	0,979579792
C4B63_26g286-t42_1	PRFA01000026	181399	182472	-	Note=2.7.3.2 (Creatine kinase)	1,0702711	0,976770538
C4B63_26g313-t42_1	PRFA01000026	136476	137069	-	protein kinase	-1,1882261	0,98194051
C4B63_27g161-t42_1	PRFA01000027	395094	396962	-	conserved hypothetical protein	-0,9286168	0,95687559
C4B63_28g345-t42_1	PRFA01000028	1749	2273	-	unspecified product	-1,2323482	0,983480878

C4B63_28g42-t42_1	PRFA01000028	93211	94020	+	conserved hypothetical protein	0,9255913	0,962358357
C4B63_28g73-t42_1	PRFA01000028	155585	156415	+	molybdopterin synthase sulphurylase-like protein(fragment)	1,2132213	0,977821058
C4B63_2g1532c-t42_1	PRFA01000002	931167	931421	+	c-Myc-binding protein homolog	1,6932466	0,994959868
C4B63_2g166-t42_1	PRFA01000002	545603	546388	+	Radial spoke protein 11	1,0564305	0,973571766
C4B63_2g288-t42_1	PRFA01000002	803665	805113	+	Kinesin-13 6	-0,9570464	0,959773371
C4B63_2g3-t42_1	PRFA01000002	18750	20270	+	Note=2.4.1.17 (Glucuronosyltransferase)	-1,0227127	0,965250236
C4B63_2g309-t42_1	PRFA01000002	844753	846180	+	NIMA-related kinase	-0,8363935	0,95555949
C4B63_2g365-t42_1	PRFA01000002	966924	968309	+	Note=2.7.12.1 (Dual-specificity kinase)	-0,9555764	0,967250944
C4B63_2g78-t42_1	PRFA01000002	151847	152350	+	conserved hypothetical protein	0,9273625	0,954632908
C4B63_2g822-t42_1	PRFA01000002	231888	233180	-	transporter	-1,0002285	0,969717894
C4B63_300g22-t42_1	PRFA01000300	13282	14427	-	Trypomastigote%2C Alanine%2C Serine and Valine rich protein (TASV)%2C subfamily C	-0,8684676	0,955482767
C4B63_301g5-t42_1	PRFA01000301	19534	22656	+	G1 cyclin CycE4	-1,1676511	0,981787063
C4B63_303g16-t42_1	PRFA01000303	557	1210	-	trypanothione reductase	-7,8839817	0,973300283
C4B63_30g110-t42_1	PRFA01000030	429560	431227	-	Probable L-cysteine desulfhydrase%2C chloroplastic	-1,051766	0,961768178
C4B63_30g147-t42_1	PRFA01000030	346585	347712	-	conserved hypothetical protein	1,1306088	0,975554769
C4B63_30g160-t42_1	PRFA01000030	318521	318997	-	MatE	-1,116611	0,955087347
C4B63_310g9-t42_1	PRFA01000310	18676	21033	+	trans-sialidase%2C Group I	-0,958318	0,95947238
C4B63_314g7-t42_1	PRFA01000314	25740	26636	-	conserved hypothetical protein	1,231797	0,982223796
C4B63_31g166-t42_1	PRFA01000031	388657	390453	-	Note=3.5.1.98 (Histone deacetylase)	-7,3237753	0,989683664
C4B63_328g12-t42_1	PRFA01000328	17465	21298	+	conserved hypothetical protein	0,9998291	0,967280453
C4B63_328g7-t42_1	PRFA01000328	5496	6023	+	conserved hypothetical protein	1,8239525	0,985817989
C4B63_328g8-t42_1	PRFA01000328	6842	7456	+	conserved hypothetical protein	1,1975482	0,980671624
C4B63_32g198-t42_1	PRFA01000032	368205	369104	-	conserved hypothetical protein	-8,016432	0,999032106
C4B63_32g200-t42_1	PRFA01000032	364648	365160	-	conserved hypothetical protein	-9,3694085	0,99937441
C4B63_32g202-t42_1	PRFA01000032	361204	363429	-	cullin	-9,8950369	0,982341832
C4B63_32g203-t42_1	PRFA01000032	358525	360939	-	Note=3.6.4.12 (DNA helicase)	-10,435261	0,991389282
C4B63_32g204-t42_1	PRFA01000032	357574	358398	-	zinc-finger multi-pass transmembrane protein	-5,7393581	0,993472616
C4B63_32g211-t42_1	PRFA01000032	349223	349717	-	60S ribosomal protein L12	0,9349329	0,965267941
C4B63_32g215-t42_1	PRFA01000032	343548	344264	-	Pentatricopeptide repeat-containing protein At2g31400%2C chloroplastic	-8,6689688	0,991123702
C4B63_32g226-t42_1	PRFA01000032	321634	322218	-	amastin	-7,9114624	0,999734419
C4B63_32g227-t42_1	PRFA01000032	318439	319023	-	amastin	-7,9114624	0,999734419
C4B63_32g228-t42_1	PRFA01000032	315244	315828	-	amastin	-7,9114624	0,999734419
C4B63_32g229-t42_1	PRFA01000032	312055	312639	-	amastin	1,0712719	0,976015109
C4B63_32g230-t42_1	PRFA01000032	308861	309445	-	amastin	1,0712719	0,976015109
C4B63_333g13-t42_1	PRFA01000333	10605	11960	-	alpha tubulin	1,061036	0,976457743

C4B63_333g15-t42_1	PRFA01000333	6389	7744	-	alpha tubulin	1,061036	0,976457743
C4B63_34g1223c-t42_1	PRFA01000034	244987	245436	-	mucin TcSMUGS	1,9344642	0,997055005
C4B63_34g1224c-t42_1	PRFA01000034	244002	244319	-	mucin TcSMUGS	1,9215747	0,991749292
C4B63_34g1225c-t42_1	PRFA01000034	243017	243334	-	mucin TcSMUGS	1,9215747	0,991749292
C4B63_34g1226c-t42_1	PRFA01000034	242032	242349	-	mucin TcSMUGS	1,657351	0,993378187
C4B63_34g1229c-t42_1	PRFA01000034	239681	239998	-	mucin TcSMUGS	1,9215747	0,991749292
C4B63_34g1232c-t42_1	PRFA01000034	235710	236027	-	mucin TcSMUGS	1,9215747	0,991749292
C4B63_34g1233c-t42_1	PRFA01000034	234725	235042	-	mucin TcSMUGS	2,0584178	0,995709396
C4B63_34g1236c-t42_1	PRFA01000034	231755	232078	-	mucin TcSMUGS	2,0526562	0,982914306
C4B63_34g1237c-t42_1	PRFA01000034	230764	231087	-	mucin TcSMUGS	2,1278426	0,997957979
C4B63_34g1239c-t42_1	PRFA01000034	228781	229104	-	mucin TcSMUGS	2,0526562	0,982914306
C4B63_34g1240c-t42_1	PRFA01000034	227793	228113	-	mucin TcSMUGS	2,3167233	0,995910057
C4B63_34g1244c-t42_1	PRFA01000034	223833	224156	-	mucin TcSMUGS	2,4863094	0,99026204
C4B63_34g216-t42_1	PRFA01000034	283881	284354	-	mucin TcSMUGS	1,9827352	0,985269122
C4B63_34g239-t42_1	PRFA01000034	240350	241492	-	mucin TcSMUGS	1,8916799	0,996635977
C4B63_34g243-t42_1	PRFA01000034	238513	239013	-	mucin TcSMUGS	1,736738	0,995620869
C4B63_34g245-t42_1	PRFA01000034	237708	238379	-	mucin TcSMUGS	1,9419428	0,996352691
C4B63_34g248-t42_1	PRFA01000034	236513	237040	-	mucin TcSMUGS	2,2205351	0,995030689
C4B63_34g263-t42_1	PRFA01000034	229590	230096	-	mucin TcSMUGS	1,897378	0,990444995
C4B63_34g269-t42_1	PRFA01000034	226803	227477	-	mucin TcSMUGS	1,7809817	0,995951369
C4B63_34g272-t42_1	PRFA01000034	225632	226135	-	mucin TcSMUGS	1,7599391	0,99575661
C4B63_34g275-t42_1	PRFA01000034	224508	225146	-	mucin TcSMUGS	2,0011966	0,997444523
C4B63_34g324-t42_1	PRFA01000034	124220	125296	-	Trypomastigote%2C Alanine%2C Serine and Valine rich protein (TASV)%2C subfamily C	-1,1712112	0,981108357
C4B63_351g27c-t42_1	PRFA01000351	12489	12884	+	small myristoylated protein-1	1,1704983	0,982058546
C4B63_35g73-t42_1	PRFA01000035	179161	179640	+	flagellar calcium-binding protein	1,308466	0,987653447
C4B63_35g78-t42_1	PRFA01000035	185719	186354	+	flagellar calcium-binding protein	1,4582997	0,991684372
C4B63_35g79-t42_1	PRFA01000035	186656	187291	+	flagellar calcium-binding protein	1,4582997	0,991684372
C4B63_35g89-t42_1	PRFA01000035	196028	196663	+	flagellar calcium-binding protein	1,211996	0,98315628
C4B63_35g90-t42_1	PRFA01000035	196965	197600	+	flagellar calcium-binding protein	1,211996	0,98315628
C4B63_35g91-t42_1	PRFA01000035	197902	198537	+	flagellar calcium-binding protein	1,211996	0,98315628
C4B63_35g92-t42_1	PRFA01000035	198839	199474	+	flagellar calcium-binding protein	1,5378828	0,984354344
C4B63_35g93-t42_1	PRFA01000035	199777	200412	+	flagellar calcium-binding protein	1,5378828	0,984354344
C4B63_35g95-t42_1	PRFA01000035	201652	202287	+	flagellar calcium-binding protein	1,4415776	0,984100567
C4B63_35g96-t42_1	PRFA01000035	202590	203225	+	flagellar calcium-binding protein	1,4415776	0,984100567
C4B63_35g97-t42_1	PRFA01000035	203527	204162	+	flagellar calcium-binding protein	1,561826	0,985670444

C4B63_35g98-t42_1	PRFA01000035	204474	205109	+	flagellar calcium-binding protein	1,5378828	0,984354344
C4B63_38g178-t42_1	PRFA01000038	373974	375116	+	UDP-Gal or UDP-GlcNAc-dependent glycosyltransferase	1,6569612	0,977655807
C4B63_38g180-t42_1	PRFA01000038	376539	377684	+	UDP-Gal or UDP-GlcNAc-dependent glycosyltransferase	1,4372975	0,967563739
C4B63_38g182-t42_1	PRFA01000038	379107	380252	+	UDP-Gal or UDP-GlcNAc-dependent glycosyltransferase	1,4372975	0,967563739
C4B63_38g184-t42_1	PRFA01000038	381677	382822	+	UDP-Gal or UDP-GlcNAc-dependent glycosyltransferase	1,4372975	0,967563739
C4B63_38g186-t42_1	PRFA01000038	384247	385392	+	UDP-Gal or UDP-GlcNAc-dependent glycosyltransferase	1,4372975	0,967563739
C4B63_38g39-t42_1	PRFA01000038	96333	97463	+	actin 3	1,3870397	0,990025968
C4B63_40g160-t42_1	PRFA01000040	321451	322506	+	conserved hypothetical protein	-0,8055646	0,951292493
C4B63_40g29-t42_1	PRFA01000040	58214	59494	+	conserved hypothetical protein	1,2374502	0,964382672
C4B63_42g269-t42_1	PRFA01000042	2202	2885	-	retrotransposon hot spot protein (RHS)	-0,8490504	0,954007318
C4B63_42g270-t42_1	PRFA01000042	540	1952	-	retrotransposon hot spot (RHS) protein	-0,9822373	0,96967068
C4B63_43g20c-t42_1	PRFA01000043	9820	10251	+	40S ribosomal protein S23	0,9717031	0,968543437
C4B63_46g74-t42_1	PRFA01000046	226605	229217	+	Note=3.4.11.- (Aminopeptidases.)	-1,0918894	0,975908876
C4B63_47g171c-t42_1	PRFA01000047	100600	100974	+	conserved hypothetical protein	0,9624373	0,961195703
C4B63_480g16-t42_1	PRFA01000480	6019	6996	-	Note=3.4.22.41 (Cathepsin F)	1,5963757	0,964364967
C4B63_48g5-t42_1	PRFA01000048	16252	19374	+	G1 cyclin CycE4	-1,1465869	0,980866383
C4B63_48g736c-t42_1	PRFA01000048	215745	216149	-	universal minicircle sequence binding protein 1	1,2705665	0,986591124
C4B63_49g928c-t42_1	PRFA01000049	86580	86942	-	conserved hypothetical protein	1,4673346	0,9875
C4B63_4g189-t42_1	PRFA01000004	744778	745956	+	Note=1.1.1.1 (Alcohol dehydrogenase)	1,1080125	0,956958215
C4B63_4g320-t42_1	PRFA01000004	681607	682962	-	conserved hypothetical protein	-1,1379282	0,980465061
C4B63_4g324-t42_1	PRFA01000004	673016	675712	-	Note=4.2.1.3 (Aconitate hydratase)	-0,8175258	0,953139754
C4B63_4g334-t42_1	PRFA01000004	656919	657797	-	Note=3.1.3.81 (Diacylglycerol diphosphate phosphatase)	-0,829365	0,954402738
C4B63_4g422-t42_1	PRFA01000004	353026	355497	-	C-terminal motor kinesin	-0,9177243	0,962989849
C4B63_4g526-t42_1	PRFA01000004	121318	123642	-	conserved hypothetical protein	-0,9231362	0,96095373
C4B63_51g126-t42_1	PRFA01000051	290772	292127	+	alpha tubulin	1,1909501	0,983238905
C4B63_52g127-t42_1	PRFA01000052	135932	137335	-	protein kinase	-0,8363858	0,954284703
C4B63_52g62-t42_1	PRFA01000052	219956	220858	+	Note=2.3.1.199 (Very-long-chain 3-oxoacyl-CoA synthase)	-0,8793079	0,960115675
C4B63_52g80-t42_1	PRFA01000052	253604	254167	+	conserved hypothetical protein	-1,0411633	0,975519358
C4B63_55g48-t42_1	PRFA01000055	132249	134051	+	Paraflagellar rod protein 2	1,6394862	0,994245751
C4B63_55g53-t42_1	PRFA01000055	137897	138490	+	paraxonemal rod protein PAR2	1,5175999	0,990315156
C4B63_56g32-t42_1	PRFA01000056	72218	74026	+	GTPase	-1,0406103	0,965627951
C4B63_57g122-t42_1	PRFA01000057	29861	30601	-	conserved hypothetical protein	1,4087839	0,975997403
C4B63_57g69-t42_1	PRFA01000057	150459	152432	-	Note=3.4.24.- (Metalloendopeptidases.)	-1,0466081	0,972084514
C4B63_57g89-t42_1	PRFA01000057	106321	108450	-	cullin-like protein	-0,8568144	0,957660529
C4B63_59g115-t42_1	PRFA01000059	171513	173183	+	stress-inducible protein STI1-like(fragment)	-0,8212721	0,952980406

C4B63_60g170-t42_1	PRFA01000060	67637	69001	-	conserved hypothetical protein	-0,9553372	0,963220019
C4B63_6g297-t42_1	PRFA01000006	806352	807446	+	conserved hypothetical protein	-8,3694085	0,95980288
C4B63_72g84-t42_1	PRFA01000072	78480	79712	-	conserved hypothetical protein	-0,9951333	0,971830737
C4B63_76g30-t42_1	PRFA01000076	70766	71263	+	prefoldin	1,0492156	0,975135741
C4B63_77g46-t42_1	PRFA01000077	93413	94468	+	Note=1.-.-.- (Oxidoreductases.)	1,5448105	0,986177998
C4B63_77g74-t42_1	PRFA01000077	141784	142839	+	Note=1.-.-.- (Oxidoreductases.)	1,0805052	0,974639991
C4B63_7g135-t42_1	PRFA01000007	703033	704319	+	Note=4.1.3.4 (Hydroxymethylglutaryl-CoA lyase)	-0,8207122	0,952850567
C4B63_7g145-t42_1	PRFA01000007	720384	723161	+	conserved hypothetical protein	-0,9500757	0,966560434
C4B63_7g249-t42_1	PRFA01000007	419156	419947	-	conserved hypothetical protein	1,3964543	0,985941926
C4B63_7g307-t42_1	PRFA01000007	308692	310686	-	Note=5.99.1.2 (Transferred entry: 5.6.2.2)	-1,0179337	0,973636686
C4B63_80g21-t42_1	PRFA01000080	52233	52694	+	Note=2.7.4.6 (Nucleoside-diphosphate kinase)	1,0617648	0,976127243
C4B63_82g79-t42_1	PRFA01000082	106332	107669	-	Note=6.1.1.2 (Tryptophan--tRNA ligase)	1,7928895	0,986343248
C4B63_82g96-t42_1	PRFA01000082	41644	41979	-	(H+)-ATPase G subunit	1,1569675	0,981692635
C4B63_82g97-t42_1	PRFA01000082	39399	40283	-	conserved hypothetical protein	1,297906	0,986715061
C4B63_82g98-t42_1	PRFA01000082	37040	38827	-	glycosyl transferase	1,0465733	0,967026676
C4B63_84g55-t42_1	PRFA01000084	74131	75309	-	Surface membrane protein	-0,9762501	0,969304769
C4B63_84g56-t42_1	PRFA01000084	72452	73624	-	Surface membrane protein	-0,9871136	0,970550047
C4B63_85g43-t42_1	PRFA01000085	110948	111886	-	conserved hypothetical protein	1,1275793	0,965651558
C4B63_85g45-t42_1	PRFA01000085	109007	109615	-	ribosomal protein S7	0,8337293	0,954432247
C4B63_86g53-t42_1	PRFA01000086	117996	119444	+	Kinesin-13 6	-0,9157066	0,960611426
C4B63_86g54-t42_1	PRFA01000086	120056	122224	+	Hypoxia up-regulated protein 1 (Fragment)	-0,9726818	0,962606232
C4B63_91g35-t42_1	PRFA01000091	94685	96493	+	GTPase	-1,0202695	0,954414542
C4B63_93g73-t42_1	PRFA01000093	54417	55850	-	Note=2.7.11.1 (Non-specific serine/threonine protein kinase)	1,0395684	0,971795326
C4B63_97g21-t42_1	PRFA01000097	59538	60053	+	60S ribosomal protein L34	1,0626588	0,976469547
C4B63_9g254-t42_1	PRFA01000009	583202	585670	-	conserved hypothetical protein	-0,9066116	0,961065864
C4B63_9g279-t42_1	PRFA01000009	530020	531597	-	Note=4.1.1.49 (Phosphoenolpyruvate carboxykinase (ATP))	1,2810038	0,985646837
C4B63_9g281-t42_1	PRFA01000009	525182	526759	-	Note=4.1.1.49 (Phosphoenolpyruvate carboxykinase (ATP))	1,2810038	0,985646837
C4B63_9g283-t42_1	PRFA01000009	520340	521917	-	Note=4.1.1.49 (Phosphoenolpyruvate carboxykinase (ATP))	1,2810038	0,985646837
C4B63_9g285-t42_1	PRFA01000009	515501	517078	-	Note=4.1.1.49 (Phosphoenolpyruvate carboxykinase (ATP))	1,2810038	0,985646837
C4B63_9g412-t42_1	PRFA01000009	228772	229227	-	40S ribosomal protein S13	0,915633	0,964701369

Table S5: Summary chromosome enrichment using Fisher's test corrected by FDR

chr	Number of genes of interest	Total number of genes	p-value	odds-ratio	adjustedP	Analysis
PRFA01000016	24	205	7,37E-23	23,2535854	1,84E-21	Epimastigote
PRFA01000032	22	161	2,52E-22	26,6019201	3,15E-21	Epimastigote
PRFA01000068	4	49	0,0003368	13,3796034	0,000935453	Epimastigote
PRFA01000077	5	41	9,83E-06	20,1854404	4,10E-05	Epimastigote
PRFA01000082	10	36	2,09E-13	48,1594196	1,30E-12	Epimastigote
PRFA01000107	4	30	5,84E-05	21,8753004	0,000208728	Epimastigote
PRFA01000110	2	38	0,0259579	8,48126683	0,04055928	Epimastigote
PRFA01000149	2	28	0,0150741	11,5166948	0,02512347	Epimastigote
PRFA01000151	9	18	4,57E-14	85,9921272	3,81E-13	Epimastigote
PRFA01000178	2	14	0,0044021	23,0331502	0,008465608	Epimastigote
PRFA01000203	3	14	0,0001524	34,8553283	0,000476305	Epimastigote
PRFA01000219	2	19	0,0075482	16,9793317	0,013478974	Epimastigote
PRFA01000220	2	12	0,0033657	26,9001585	0,00701178	Epimastigote
PRFA01000241	3	21	0,0004394	23,2300028	0,001098519	Epimastigote
PRFA01000328	4	15	5,25E-06	43,7445263	2,63E-05	Epimastigote
PRFA01000341	2	12	0,0033657	26,9001585	0,00701178	Epimastigote
PRFA01000016	5	205	0,0003304	9,27925827	0,000660884	Stress
PRFA01000032	20	161	3,09E-27	69,4726361	3,71E-26	Stress
PRFA01000068	6	49	1,07E-08	47,9178241	4,28E-08	Stress
PRFA01000077	4	41	8,19E-06	36,6614267	2,46E-05	Stress
PRFA01000082	3	36	0,0001885	30,671693	0,000452473	Stress
PRFA01000151	6	18	5,31E-11	130,376251	3,19E-10	Stress
PRFA01000016	35	205	3,28E-32	23,1282989	1,15E-30	Adhered
PRFA01000032	25	161	3,17E-22	19,5961399	5,55E-21	Adhered
PRFA01000068	13	49	1,10E-14	31,0582222	7,69E-14	Adhered
PRFA01000077	8	41	1,35E-08	22,1435302	7,90E-08	Adhered
PRFA01000082	15	36	3,31E-19	49,4115803	3,86E-18	Adhered
PRFA01000107	4	30	0,0002525	14,7711792	0,000888405	Adhered
PRFA01000151	12	18	1,72E-17	77,5717902	1,50E-16	Adhered
PRFA01000178	4	14	1,87E-05	31,6603775	8,17E-05	Adhered
PRFA01000182	3	11	0,0002538	30,046117	0,000888405	Adhered
PRFA01000203	2	14	0,009169	15,6458368	0,021394334	Adhered
PRFA01000219	2	19	0,0155737	11,5263008	0,034067529	Adhered
PRFA01000220	3	12	0,0003152	27,5208025	0,001002781	Adhered
PRFA01000225	2	14	0,009169	15,6458368	0,021394334	Adhered
PRFA01000241	5	21	3,39E-06	26,5233734	1,70E-05	Adhered
PRFA01000328	3	15	0,0005539	22,0351466	0,001615615	Adhered
PRFA01000341	2	12	0,0070369	18,2496384	0,018945552	Adhered
PRFA01000016	16	205	2,93E-08	6,34542384	1,20E-06	M. trypomastigote
PRFA01000032	12	161	2,57E-06	5,96620706	4,21E-05	M. trypomastigote
PRFA01000034	22	167	4,39E-15	11,0311562	3,60E-13	M. trypomastigote
PRFA01000035	12	148	1,13E-06	6,49480468	2,31E-05	M. trypomastigote
PRFA01000082	4	36	0,0017011	8,64476633	0,013948704	M. trypomastigote
PRFA01000110	4	38	0,002042	8,19042933	0,015222215	M. trypomastigote
PRFA01000149	4	28	0,0007254	11,1191127	0,007435039	M. trypomastigote
PRFA01000178	5	14	3,40E-06	27,9230615	4,65E-05	M. trypomastigote

PRFA01000219	5	19	1,18E-05	20,5657711	0,000138181	M. trypomastigote
PRFA01000241	6	21	1,01E-06	22,4252731	2,31E-05	M. trypomastigote
PRFA01000328	3	15	0,00149	15,5125336	0,013575546	M. trypomastigote

Table S6: Differentially expressed genes (FDR < 5%) in common among all stages in the comparison KO vs WT

trancriptID(Dm28c2018)	logFC (KO/WT)	logCPM	p-value	FDR
C4B63_32g226-t42_1	-7,305302297	4,0702009	2,66E-18	1,45E-14
C4B63_32g227-t42_1	-7,305302297	4,0702009	2,66E-18	1,45E-14
C4B63_32g228-t42_1	-7,305302297	4,0702009	2,66E-18	1,45E-14
C4B63_32g181-t42_1	-7,881171311	3,687364765	1,77E-17	5,87E-14
C4B63_32g198-t42_1	-7,618564037	5,19865495	1,80E-17	5,87E-14
C4B63_31g166-t42_1	-7,274856952	4,671797174	4,56E-16	1,24E-12
C4B63_32g200-t42_1	-8,334090596	4,011118936	1,22E-15	2,85E-12
C4B63_32g204-t42_1	-11,15286309	4,054238837	7,11E-14	1,45E-10
C4B63_32g203-t42_1	-7,220055692	5,338389776	2,32E-13	4,19E-10
C4B63_32g186-t42_1	-7,681543543	1,909768443	8,87E-13	1,44E-09
C4B63_32g177-t42_1	-5,230059417	2,60723897	1,37E-11	2,03E-08
C4B63_32g215-t42_1	-10,57732121	3,836596737	1,04E-10	1,41E-07
C4B63_32g189-t42_1	-9,212392654	1,636329885	4,94E-10	6,19E-07
C4B63_32g202-t42_1	-6,682997819	5,126629004	5,52E-10	6,42E-07
C4B63_32g229-t42_1	0,974690033	5,2019229	1,87E-09	1,90E-06
C4B63_32g230-t42_1	0,974690033	5,2019229	1,87E-09	1,90E-06
C4B63_32g211-t42_1	1,068292989	7,817879084	7,92E-09	7,59E-06
C4B63_151g38-t42_1	1,174064681	4,871170814	9,87E-09	8,93E-06
C4B63_32g139-t42_1	-1,526380642	3,923849191	1,41E-08	1,21E-05
C4B63_77g67-t42_1	0,76526782	6,830923607	7,48E-08	6,10E-05
C4B63_1g819-t42_1	-1,294212031	3,741142855	3,89E-07	0,0003015
C4B63_107g93-t42_1	-1,821393634	3,316437745	4,11E-07	0,0003047
C4B63_151g29-t42_1	1,007107535	6,896501263	5,64E-07	0,0003996
C4B63_32g171-t42_1	-6,538484469	2,082148449	6,55E-07	0,0004438
C4B63_32g187-t42_1	-3,793713743	1,469120498	6,81E-07	0,0004438
C4B63_256g19-t42_1	1,146509357	4,477532945	7,30E-07	0,000452
C4B63_77g75-t42_1	0,668755236	8,14258497	7,49E-07	0,000452
C4B63_85g45-t42_1	0,999083965	8,170408634	8,47E-07	0,0004928
C4B63_32g205-t42_1	-1,260605623	5,316541236	9,12E-07	0,000512
C4B63_68g103-t42_1	0,858712241	6,527729911	9,54E-07	0,0005178
C4B63_16g148-t42_1	0,992350377	5,040697212	1,39E-06	0,0007319
C4B63_16g33-t42_1	0,692730515	6,369074776	1,79E-06	0,0008944
C4B63_16g140-t42_1	0,723597579	6,369064087	1,81E-06	0,0008944
C4B63_151g35-t42_1	1,00417426	5,147972486	3,39E-06	0,0016221
C4B63_32g185-t42_1	-3,486243976	0,716238588	3,77E-06	0,0017552
C4B63_68g83-t42_1	0,810381721	5,885872407	4,78E-06	0,0021643
C4B63_16g139-t42_1	0,775165509	5,007864882	6,05E-06	0,0026646
C4B63_16g331-t42_1	0,736503873	7,721113623	8,12E-06	0,003482
C4B63_82g75-t42_1	0,817783932	6,790933303	8,97E-06	0,0037448
C4B63_151g47-t42_1	0,864585919	4,524299151	9,93E-06	0,0040435
C4B63_77g60-t42_1	0,683120037	7,061831667	1,03E-05	0,0040825
C4B63_151g34-t42_1	0,744588504	6,759791918	1,14E-05	0,0044182
C4B63_341g11-t42_1	1,084550539	3,729824711	1,27E-05	0,0046931
C4B63_32g138-t42_1	-3,755956288	1,304065108	1,50E-05	0,0054167
C4B63_151g48-t42_1	0,882396415	5,395842093	1,81E-05	0,0064236
C4B63_16g330-t42_1	0,717818919	7,27057522	1,89E-05	0,0065535
C4B63_151g36-t42_1	1,215666142	4,302910945	1,96E-05	0,0066371

C4B63_149g41-t42_1	1,205627763	4,581390123	2,04E-05	0,006769
C4B63_16g86-t42_1	0,644061051	6,780713528	2,30E-05	0,0075036
C4B63_31g167-t42_1	-1,097446572	4,787886415	2,38E-05	0,0075936
C4B63_1g818-t42_1	-1,688078504	1,834836919	2,43E-05	0,0076239
C4B63_16g147-t42_1	0,615429409	6,295008411	2,73E-05	0,0083897
C4B63_219g48-t42_1	0,576533079	6,744522086	2,81E-05	0,0084888
C4B63_82g448c-t42_1	0,715712098	5,970797274	3,00E-05	0,0088978
C4B63_151g33-t42_1	0,829365018	6,409210946	3,25E-05	0,0094499
C4B63_1g629-t42_1	-1,834254316	1,252624701	3,34E-05	0,0095568
C4B63_82g94-t42_1	0,809687541	5,038373021	3,52E-05	0,0096694
C4B63_16g11-t42_1	0,813641334	6,109916875	3,56E-05	0,0096694
C4B63_328g10-t42_1	0,649055175	7,964375935	3,56E-05	0,0096694
C4B63_16g142-t42_1	0,774639941	4,920426799	4,32E-05	0,0115402
C4B63_107g29-t42_1	-1,838118634	1,781092718	5,72E-05	0,0147833
C4B63_16g742c-t42_1	0,791908908	5,028791302	5,82E-05	0,0148164
C4B63_43g95-t42_1	0,893355436	5,189125019	6,50E-05	0,0162871
C4B63_182g5-t42_1	0,751075933	7,335096262	6,78E-05	0,0167381
C4B63_151g41-t42_1	1,133700742	2,87589335	8,27E-05	0,0201028
C4B63_341g12-t42_1	0,877009906	4,779302554	8,46E-05	0,020268
C4B63_219g47-t42_1	0,560339488	6,865063038	0,00012542	0,029185
C4B63_82g114-t42_1	0,649453244	5,872898295	0,00017455	0,0400467
C4B63_1g813-t42_1	-1,72495313	1,122619792	0,00019047	0,0430915
C4B63_178g41-t42_1	0,64711006	8,494637655	0,00021326	0,0469424
C4B63_68g91-t42_1	0,579803142	6,40469207	0,00021712	0,047155
C4B63_68g120-t42_1	0,583298485	6,317154204	0,00022053	0,0472364
C4B63_178g46-t42_1	0,662349887	6,143664031	0,00022329	0,0472364
C4B63_1g806-t42_1	-1,349608577	2,442121242	0,00023286	0,0486278

Table S7: Sequence coverage of *T. cruzi* histones

Histone	Epimastigote Wild type (%)	Epimastigote KO (%)	Metacyclic Trypomastigote Wild type (%)	Metacyclic Trypomastigote KO (%)
H2A	72	79	67	60
H2B	85	85	80	80
H3	80	80	80	72
H4	84	76	71	71
H1	30	30	30	30
H2AZ	45	45	43	43
H2BV	30	30	30	30
H3V	62	62	62	62

Table S8: Summary of type and number of T. cruzi PTMs in histones and histone variants

		Me	Me2	Me3	Ac	Ph	Hib	Su	Ub	Cit	OH	Total
**H2A**	New	4	4	3	7	3	2	1	2		1	27
	**This work total**	**6**	**6**	**4**	**14**	**3**	**2**	**1**	**2**		**1**	**40**
	Literature	6	1	1	5		1			3		17
	Total	12	7	5	19	3	3	1	2	4	1	57
**H2B**	New	5	5	4	15	3	2			1		35
	**This work total**	**9**	**6**	**5**	**21**	**3**	**3**			**2**	**1**	**50**
	Literature	4	8	1	4	3			2	1		23
	Total	13	14	6	25	6	3		2	3	1	73
**H3**	New	4	4	1	2	1			1	1		14
	**This work total**	**8**	**8**	**4**	**6**	**2**			**1**	**2**	**1**	**32**
	Literature	9	5	2	10		1	1	1	4		33
	Total	17	13	9	16	2	1	1	2	6	1	65
**H4**	New	2	4	4	5	1		1	1			18
	**This work total**	**8**	**6**	**4**	**14**	**1**		**1**	**1**	**1**	**3**	**39**
	Literature	5	2	1	10	2	2			3	2	27
	Total	13	8	5	24	3	2	1	1	4	5	66
**H2AZ**	New				1							1
	**This work total**				**4**							**4**
	Literature		2		11	6						19
	Total		2		15	6						23
**H2BV**	New						2					2
	**This work total**						**2**					**2**
	Literature	3	4	1	8							16
	Total	3	4	1	8		2					18
**H3V**	New	2	2		3	2						9
	**This work total**	**2**	**5**	**2**	**4**	**2**						**15**
	Literature	3	1		2							6
	Total	5	6	2	6	2						21
**H1**	New				1	1			1			3
	**This work total**				**1**	**1**			**1**			**3**
	Literature		2	3	6	1						12
	Total		2	3	7	2			1			15

Table S9: PSM normalized Areas

				EPI WT1	EPI WT2	EPI WT3	EPI WT4	EPI WT5	EPI WT6	EPI WT7	EPI WT8	EPI KO1	EPI KO2	EPI KO3	EPI KO4	EPI KO5	EPI KO6	EPI KO7	EPI KO8	META WT1	META WT5	META KO1	META KO5
H2B	K20	Acetylation (K)	H2BK20Acetylation (K)	16785,71	0	0	0	0	0	20536,64	0	0	0	0	0	0	0	19365,43	40654,21	0	0	0	0
H2B	K22	Acetylation (K)	H2BK22Acetylation (K)	16785,71	0	0	0	0	0	20536,64	0	0	0	0	0	0	0	19365,43	40654,21	0	0	0	0
H2B	R23	Dimethylation(KR)	H2BR23Dimethylation(KR)	0	0	0	0	0	0	20536,64	0	0	0	0	0	0	0	0	0	0	0	0	0
H2B	R23	Trimethylation	H2BR23Trimethylation	0	0	0	0	0	0	0	0	0	9465,021	11398,28	0	0	0	0	0	0	0	0	0
H2B	T24	Acetylation (TSCYH)	H2BT24Acetylation (TSCYH)	34553,57	55906,04	38892,31	51749,27	113888,9	67809,73	29721,36	60875,51	29288,03	34567,9	41948,31	75373,62	56205,25	53265,87	8905,908	0	48107,45	130111,5	53364,27	59785,2
H2B	Y28	Hydroxylation	H2BY28Hydroxylation	13125	691,2752	17784,62	10422,74	3738,426	0	17440,66	0	25404,53	693,7096	44135,19	18453,54	38782,82	782,8887	49343,54	0	0	13011,15	0	43078,76
H2B	S30	Acetylation (TSCYH)	H2BS30Acetylation (TSCYH)	45446,43	58389,26	75076,92	42784,26	129629,6	67588,5	118679,1	22161,42	56148,87	81716,64	72233,27	48862,9	115871,1	134314,6	122538,3	49766,36	46642,25	106195,8	41183,29	103580
H2B	R31	Methylation(KR)	H2BR31Methylation(KR)	45446,43	58389,26	75076,92	42784,26	129629,6	67588,5	118679,1	22161,42	56148,87	81716,64	72233,27	48862,9	115871,1	134314,6	122538,3	49766,36	46642,25	106195,8	41183,29	103580
H2B	S32	Acetylation (TSCYH)	H2BS32Acetylation (TSCYH)	0	4651,007	3464,615	3367,347	4710,648	0	1444,788	16005,47	1618,123	11111,11	821,7362	0	0	0	18599,56	0	0	0	0	8579,952
H2B	K34	Acetylation (K)	H2BK34Acetylation (K)	0	0	0	0	0	0	0	0	0	0	0	0	0	0	0	0	0	188,3519	0	110,2625
H2B	K34	Methylation(KR)	H2BK34Methylation(KR)	2196,429	0	0	0	7708,333	4568,584	24148,61	2982,216	0	0	0	0	0	6228,151	8763,676	6086,449	0	11078,07	0	4594,272
H2B	K34	Trimethylation	H2BK34Trimethylation	0	0	0	0	0	0	0	0	0	0	0	0	0	0	0	0	0	0	0	3663,484
H2B	S35	Acetylation (TSCYH)	H2BS35Acetylation (TSCYH)	0	10536,91	0	0	0	0	1444,788	0	0	0	650,7621	0	0	0	0	0	0	1734,82	0	0
H2B	S35	Phosphorylation (STY)	H2BS35Phosphorylation (STY)	0	0	0	0	0	0	20330,24	0	0	0	0	0	0	0	0	0	0	0	0	0
H2B	S41	Acetylation (TSCYH)	H2BS41Acetylation (TSCYH)	0	0	5827,692	0	0	0	0	0	0	11757,79	0	0	0	10763,57	0	0	8717,949	0	758,7007	10942,72
H2B	S43	Acetylation (TSCYH)	H2BS43Acetylation (TSCYH)	96428,57	16442,95	10092,31	18513,12	210648,1	35840,71	100928,8	2202,462	50080,91	113462,7	52683,9	7537,362	16229,12	64029,44	48030,63	7348,131	39804,64	81164,81	8225,058	43556,09
H2B	R45	Citrullination	H2BR45Citrullination	0	0	613,5385	0	0	0	0	0	0	0	0	0	0	0	0	0	0	0	0	0
H2B	R45	Methylation(KR)	H2BR45Methylation(KR)	357142,9	227516,8	329230,8	191691	756944,4	384955,8	484004,1	160054,7	372977,3	400940,6	341948,3	280052	618138,4	648574,1	489059,1	302570,1	321123,3	565055,8	272621,8	468973,7
H2B	R45	Dimethylation(KR)	H2BR45Dimethylation(KR)	0	0	5827,692	0	0	1,106195	0	0	0	11757,79	0	0	0	10763,57	0	0	8717,949	0	758,7007	10942,72
H2B	T46	Acetylation (TSCYH)	H2BT46Acetylation (TSCYH)	0	71,14094	0	0	97,91667	0	0	0	0	0	0	12,47563	379,4749	37,07452	0	0	0	0	0	3,854415
H2B	K48	2-Hydroxyisobutyrylation	H2BK482-Hydroxyisobutyrylation	390,1786	2402,685	6215,385	1603,499	0	827,4336	0	2393,981	329,288	3139,33	10404,24	8901,884	1431,981	3229,071	1487,965	1460,28	1514,042	0	1118,329	0
H2B	K48	Acetylation (K)	H2BK48Acetylation (K)	0	0	800	0	39,35185	0	75,5418	0	0	0	2379,059	0	0	0	71,11597	0	1098,901	0	0	0
H2B	K48	Methylation(KR)	H2BK48Methylation(KR)	874,1071	55503,36	107076,9	0	278935,2	202433,6	194014,4	0	0	8818,342	137839,6	113710,2	263723,2	119595,2	196936,5	289719,6	0	297397,8	631,0905	156324,6
H2B	K48	Dimethylation(KR)	H2BK48Dimethylation(KR)	2178,571	347,651	843,0769	0	0	1,106195	10835,91	0	0	0	0	1000,65	1252,983	1113,155	2275,711	370,3271	0	0	0	0
H2B	K48	Trimethylation	H2BK48Trimethylation	158,0357	410,0671	806,1538	0	0	0	0	0	0	0	0	0	175,4177	0	0	0	0	1141,264	0	0
H2B	S52	Acetylation (TSCYH)	H2BS52Acetylation (TSCYH)	0	0	0	0	0	827,4336	0	0	0	0	0	0	0	0	0	0	0	0	0	0
H2B	S52	Phosphorylation (STY)	H2BS52Phosphorylation (STY)	0	0	0	0	47337,96	0	59339,53	0	0	0	0	7407,407	42959,43	0	83807,44	84696,26	0	0	0	0
H2B	R60	Citrullination	H2BR60Citrullination	6258,929	2530,201	9169,231	3360,058	29166,67	2500	8369,453	231,1902	10194,17	7231,041	4824,387	6068,876	25536,99	13615,46	2319,475	5151,869	3980,464	5613,383	2366,589	4892,601
H2B	T67	Acetylation (TSCYH)	H2BT67Acetylation (TSCYH)	5392,857	6315,436	10584,62	2179,3	4803,241	6128,319	0	6224,35	5647,249	8641,975	8018,555	3339,831	0	13799,45	9584,245	11425,23	3492,063	9157,373	10336,43	0
H2B	R70	Methylation(KR)	H2BR70Methylation(KR)	5392,857	6315,436	10584,62	2179,3	4803,241	6128,319	0	6224,35	5647,249	8641,975	8018,555	3339,831	0	13799,45	9584,245	11425,23	3492,063	9157,373	10336,43	0
H2B	K73	Acetylation (K)	H2BK73Acetylation (K)	0	0	0	0	0	0	0	0	0	0	0	0	0	0	0	0	0	0	0	0
H2B	K74	Acetylation (K)	H2BK74Acetylation (K)	0	0	0	0	0	0	0	0	0	0	0	0	0	0	0	0	0	0	0	0
H2B	R75	Methylation(KR)	H2BR75Methylation(KR)	0	0	0	0	0	0	0	0	0	0	0	0	0	2677,093	0	0	0	0	0	0
H2B	R75	Dimethylation(KR)	H2BR75Dimethylation(KR)	3205,357	0	4787,692	1610,787	0	0	0	0	0	0	0	0	0	0	0	0	10488,4	0	0	0
H2B	R75	Trimethylation	H2BR75Trimethylation	0	0	0	0	0	0	0	0	0	0	0	0	0	0	0	0	0	0	0	0
H2B	T76	Acetylation (TSCYH)	H2BT76Acetylation (TSCYH)	0	0	720	0	0	0	0	0	0	946,5021	0	760,2339	0	0	0	0	0	0	0	0
H2B	R80	Methylation(KR)	H2BR80Methylation(KR)	0	0	720	0	0	0	0	0	0	946,5021	0	760,2339	0	0	0	0	0	0	0	0
H2B	R80	Dimethylation(KR)	H2BR80Dimethylation(KR)	0	0	0	0	0	0	0	0	0	0	354,5394	0	0	0	0	0	0	0	0	0
H2B	K96	2-Hydroxyisobutyrylation	H2BK962-Hydroxyisobutyrylation	0	0	0	0	0	0	0	0	0	0	7024,52	0	0	0	0	0	0	0	0	0
H2B	K96	Acetylation (K)	H2BK96Acetylation (K)	0	0	0	0	0	0	0	0	0	0	0	0	584,7255	565,7774	0	0	0	0	0	0
H2B	K96	Methylation(KR)	H2BK96Methylation(KR)	0	0	0	0	0	0	0	0	0	0	15573,23	0	0	0	0	0	0	0	0	30190,93
H2B	H97	Acetylation (TSCYH)	H2BH97Acetylation (TSCYH)	54,375	102,0134	0	0	0	0	0	0	0	0	22531,48	0	72195,7	793,9282	0	0	1067,155	990,0867	0	30190,93
H2B	T103	Acetylation (TSCYH)	H2BT103Acetylation (TSCYH)	602678,6	791946,3	1655385	675656	156250	27212,39	1052632	0	182038,8	201058,2	1610338	955165,7	264916,5	1481141	959518,6	121495,3	75702,08	1363073	660092,8	110501,2
H2B	T103	Phosphorylation (STY)	H2BT103Phosphorylation (STY)	0	0	0	0	0	0	0	0	0	0	0	0	71599,05	0	0	0	0	0	0	0
H2B	K104	2-Hydroxyisobutyrylation	H2BK1042-Hydroxyisobutyrylation	3133,929	0	0	0	0	0	0	5170,999	0	0	0	0	0	0	0	12032,71	0	0	0	3293,556
H2B	K104	Methylation(KR)	H2BK104Methylation(KR)	0	0	0	0	0	0	0	0	0	44,79718	0	0	0	0	0	0	0	0	0	0
H2B	K104	Dimethylation(KR)	H2BK104Dimethylation(KR)	0	0	2843,077	0	0	0	0	0	0	1457,966	0	0	71599,05	0	0	0	5970,696	0	0	0
H2B	K104	Trimethylation	H2BK104Trimethylation	0	0	0	0	0	0	0	0	0	0	9343,936	10786,22	0	0	0	0	0	0	0	0
H2B	S107	Acetylation (TSCYH)	H2BS107Acetylation (TSCYH)	881,25	0	2916,923	0	0	0	0	0	0	0	0	0	0	0	0	0	5970,696	0	0	0
H2B	H108	Acetylation (TSCYH)	H2BH108Acetylation (TSCYH)	0	0	1255,385	0	0	0	0	0	0	0	0	0	0	0	0	0	5970,696	0	0	0
H3	K19	Ubiquitination	H3K19Ubiquitination	0	0	0	0	0	0	0	0	719,2557	0	0	18778,43	0	0	0	0	0	0	0	0
H3	K20	Acetylation (K)	H3K20Acetylation (K)	0	0	0	0	0	0	110423,1	0	0	0	0	0	0	0	59518,6	52453,27	0	0	0	1193,317
H3	K23	Methylation(KR)	H3K23Methylation(KR)	0	0	0	0	0	0	0	0	0	0	11928,43	0	0	1287,948	0	0	0	0	0	0
H3	K23	Dimethylation(KR)	H3K23Dimethylation(KR)	0	0	5729,231	6698,251	0	0	50877,19	0	2071,197	3462,669	12789,93	870,6953	0	0	0	57827,1	0	3717,472	29814,39	0
H3	K23	Trimethylation	H3K23Trimethylation	0	0	0	0	0	0	110423,1	0	0	0	0	0	0	0	59518,6	52453,27	0	0	0	0

H3	T29	Acetylation (TSCYH)	H3T29Acetylation (TSCYH)	0	290,604	0	0	0	0	1331,269	0	470,8738	593,7684	600,3976	656,2703	0	1517,939	1126,915	0	492,0635	0	0	0
H3	T29	Phosphorylation (STY)	H3T29Phosphorylation (STY)	0	0	0	0	0	1261,062	0	0	0	0	0	0	0	0	1017,505	0	0	0	0	0
H3	K32	Methylation(KR)	H3K32Methylation(KR)	0	0	0	0	0	1261,062	0	0	0	823,0453	0	0	0	0	1017,505	0	0	0	0	0
H3	R37	Dimethylation(KR)	H3R37Dimethylation(KR)	0	0	0	0	0	0	0	0	0	1487,36	0	0	0	0	0	0	0	0	0	0
H3	R39	Dimethylation(KR)	H3R39Dimethylation(KR)	5348,214	16040,27	21846,15	12390,67	43055,56	32411,5	52941,18	25991,79	13025,89	20987,65	30881,38	12215,72	25417,66	11959,52	41028,45	11471,96	8058,608	45105,33	17517,4	25059,67
H3	R53	Methylation(KR)	H3R53Methylation(KR)	1151,786	0	0	903,7901	2673,611	4026,549	4520,124	0	0	2704,292	0	0	2243,437	6062,557	3260,394	2149,533	0	3779,43	0	5000
H3	R53	Dimethylation(KR)	H3R53Dimethylation(KR)	0	0	0	0	2395,833	0	0	842,6813	0	605,5262	0	0	0	0	0	1927,57	0	0	0	2720,764
H3	T55	Acetylation (TSCYH)	H3T55Acetylation (TSCYH)	0	0	0	0	0	0	0	0	0	1205,173	0	0	0	0	0	0	0	0	0	0
H3	K61	Acetylation (K)	H3K61Acetylation (K)	0	1046,98	2203,077	954,8105	3888,889	2411,504	3199,174	839,9453	0	2657,26	735,5865	2339,181	1503,58	1554,738	3041,575	4415,888	2039,072	1833,953	1160,093	3066,826
H3	K61	Methylation(KR)	H3K61Methylation(KR)	0	0	0	0	0	0	0	0	0	0	0	0	0	0	11269,15	0	0	0	0	0
H3	K61	Dimethylation(KR)	H3K61Dimethylation(KR)	0	0	0	0	12037,04	0	7595,459	0	0	0	0	0	0	0	0	0	0	0	0	0
H3	K61	Trimethylation	H3K61Trimethylation	0	0	0	0	11192,13	0	0	0	0	5808,348	4618,953	0	0	0	0	6285,047	0	8128,872	0	0
H3	R66	Citrullination	H3R66Citrullination	17946,43	12416,11	14523,08	8309,038	32986,11	17146,02	16615,07	5006,84	19498,38	23398	17428,76	22871,99	22673,03	32198,71	20240,7	23714,95	7680,098	11809,17	4269,142	11801,91
H3	R66	Methylation(KR)	H3R66Methylation(KR)	0	0	0	0	0	0	0	0	0	0	0	0	0	0	0	1577,103	0	0	0	0
H3	R66	Dimethylation(KR)	H3R66Dimethylation(KR)	0	0	0	0	0	0	0	0	0	0	1742,876	0	0	0	0	0	0	0	0	0
H3	S72	Acetylation (TSCYH)	H3S72Acetylation (TSCYH)	2258,929	0	0	0	7025,463	0	995,872	0	0	0	0	0	0	0	0	0	0	0	0	0
H3	K76	Dimethylation(KR)	H3K76Dimethylation(KR)	80,17857	0	42,76923	0	0	2035,398	1795,666	0	0	0	0	0	0	1241,95	0	0	0	0	0	0
H3	K76	Trimethylation	H3K76Trimethylation	0	353,0201	0	145,7726	0	0	995,872	0	0	0	0	0	0	0	0	0	501,8315	0	0	0
H3	R80	Trimethylation	H3R80Trimethylation	0	0	0	0	0	0	0	0	0	0	0	0	0	0	0	0	0	0	335,2668	0
H3	Y96	Hydroxylation	H3Y96Hydroxylation	0	0	0	0	0	0	0	0	0	0	0	3014,945	0	0	0	0	0	0	0	0
H3	R106	Methylation(KR)	H3R106Methylation(KR)	0	0	0	0	866,8981	0	0	0	0	0	0	0	0	0	0	0	0	0	0	0
H3	T115	Phosphorylation (STY)	H3T115Phosphorylation (STY)	0	1348,993	646,1538	0	0	0	0	0	0	4973,545	3677,932	9486,68	0	0	0	0	0	0	404,8724	0
H3	K119	Methylation(KR)	H3K119Methylation(KR)	0	1348,993	646,1538	0	0	0	0	0	0	752,4985	1636,846	0	0	0	0	0	0	0	404,8724	0
H3	C126	Acetylation (TSCYH)	H3C126Acetylation (TSCYH)	41875	207382,6	197538,5	53862,97	230324,1	24889,38	21878,22	1110,807	122168,3	289829,5	456593,8	385964,9	85560,86	64213,43	88840,26	58411,21	168498,2	128872,4	68909,51	50119,33
H3	R128	Citrullination	H3R128Citrullination	0	0	0	0	0	0	0	0	0	0	579,1915	0	0	0	0	0	0	0	0	0
H3	R128	Methylation(KR)	H3R128Methylation(KR)	28392,86	197986,6	195692,3	57215,74	180555,6	24889,38	21878,22	1110,807	100323,6	257495,6	447316,1	398960,4	85560,86	55197,79	78774,62	43925,23	168498,2	105948	60324,83	50119,33
H3	R128	Dimethylation(KR)	H3R128Dimethylation(KR)	0	0	0	0	0	0	0	0	0	284,5385	0	252,7615	0	0	0	0	0	0	0	0
H2A	R18	Trimethylation	H2AR18Trimethylation	0	0	0	0	0	0	0	0	0	0	0	0	0	0	0	0	0	0	0	0
H2A	S19	Acetylation (TSCYH)	H2AS19Acetylation (TSCYH)	28214,29	34228,19	43076,92	27332,36	59837,96	42809,73	38596,49	18467,85	30582,52	40681,95	49900,6	52566,6	39140,81	53909,84	47155,36	29088,79	38583,64	45972,74	23201,86	43914,08
H2A	S19	Phosphorylation (STY)	H2AS19Phosphorylation (STY)	17142,86	27583,89	0	10131,2	0	38384,96	58204,33	0	0	0	8349,901	0	59188,54	38270,47	32494,53	0	0	42007,43	14501,16	0
H2A	K21	Succinylation	H2AK21Succinylation	0	3107,383	7200	0	10810,19	7533,186	8740,97	5444,596	0	12757,2	3691,186	6562,703	0	0	8172,867	6401,869	3919,414	0	4060,325	8651,551
H2A	K21	Acetylation (K)	H2AK21Acetylation (K)	1107,143	1993,289	2430,769	1625,364	0	0	0	0	1949,838	2557,319	2365,805	2001,3	0	0	2592,998	0	1040,293	0	1809,745	0
H2A	K21	Methylation(KR)	H2AK21Methylation(KR)	0	0	0	0	0	0	0	0	0	0	0	0	0	0	0	2967,29	0	0	0	0
H2A	K21	Dimethylation(KR)	H2AK21Dimethylation(KR)	0	69,79866	0	7288,63	0	19137,17	0	0	0	168,7243	0	167,6413	0	175,713	0	0	89,98779	0	0	1527,446
H2A	K21	Trimethylation	H2AK21Trimethylation	17142,86	27651,01	0	10131,2	0	38384,96	58204,33	0	10760,52	799,5297	8349,901	0	59188,54	38270,47	32494,53	0	16239,32	34572,49	22853,83	0
H2A	R30	Methylation(KR)	H2AR30Methylation(KR)	0	145,6376	118,1538	685,1312	0	0	0	0	0	168,7243	1583,83	260,5588	0	1977,921	0	0	0	0	1392,111	0
H2A	R30	Dimethylation(KR)	H2AR30Dimethylation(KR)	0	0	0	0	0	0	0	649,7948	0	0	0	199,4802	0	0	0	0	0	0	0	0
H2A	S33	Acetylation (TSCYH)	H2AS33Acetylation (TSCYH)	1446,429	2503,356	1981,538	750,7289	0	4900,442	2941,176	0	1860,841	168,7243	302,8496	5224,172	2971,36	6007,36	2592,998	5280,374	1636,142	0	2540,603	3365,155
H2A	R36	Dimethylation(KR)	H2AR36Dimethylation(KR)	0	0	0	0	0	0	0	649,7948	0	0	0	199,4802	0	0	0	0	0	0	0	0
H2A	R36	Trimethylation	H2AR36Trimethylation	0	0	0	0	0	0	0	0	0	0	1583,83	0	0	0	0	0	0	0	0	0
H2A	Y51	Hydroxylation	H2AY51Hydroxylation	0	85,90604	0	75,80175	0	500	0	2626,539	0	0	0	0	0	0	0	0	0	0	0	0
H2A	R84	Methylation(KR)	H2AR84Methylation(KR)	2107,143	5859,06	6215,385	0	0	0	6759,546	0	2540,453	6584,362	5619,616	6120,858	8305,489	12511,5	4332,604	2733,645	3980,464	0	4605,568	10358
H2A	R84	Dimethylation(KR)	H2AR84Dimethylation(KR)	0	0	0	0	0	0	0	0	0	0	0	0	0	0	0	0	0	0	2598,608	0
H2A	T85	Acetylation (TSCYH)	H2AT85Acetylation (TSCYH)	1223,214	5859,06	2560	0	0	0	669,7626	0	1221,683	4726,631	5619,616	4152,047	4498,807	7589,696	4332,604	0	2136,752	0	4605,568	10358
H2A	R91	Methylation(KR)	H2AR91Methylation(KR)	0	0	0	0	0	0	0	0	0	0	0	87,06953	0	0	0	0	0	0	0	0
H2A	H92	Acetylation (TSCYH)	H2AH92Acetylation (TSCYH)	5008,929	5033,557	7692,308	3163,265	20601,85	4756,637	8431,373	0	11569,58	13639,04	2776,673	1630,929	32816,23	9751,61	0	5981,308	2576,313	3469,641	3410,673	13603,82
H2A	K101	Acetylation (K)	H2AK101Acetylation (K)	473,2143	661,745	1870,769	918,3673	0	1117,257	2321,981	0	1189,32	1622,575	1338,635	478,2326	0	2474,701	657,5492	720,7944	495,7265	0	569,6056	0
H2A	K101	Methylation(KR)	H2AK101Methylation(KR)	46607,14	48187,92	89846,15	33746,36	179398,1	136061,9	125903	0	92233,01	88771,31	43605,04	59194,28	146778	202391,9	144420,1	121495,3	32234,43	163568,8	49303,94	105131,3
H2A	K101	Dimethylation(KR)	H2AK101Dimethylation(KR)	0	0	683,0769	0	0	0	0	0	922,3301	0	3247,184	0	0	3946,642	0	0	0	0	27,37819	0
H2A	T104	Acetylation (TSCYH)	H2AT104Acetylation (TSCYH)	0	0	0	0	0	0	0	0	0	0	0	0	0	0	0	0	0	0	0	0
H2A	T104	Phosphorylation (STY)	H2AT104Phosphorylation (STY)	46607,14	32147,65	94769,23	33746,36	197916,7	104203,5	63570,69	0	101132,7	88771,31	43605,04	54905,78	195704,1	153633,9	74070,02	121495,3	32234,43	66542,75	49303,94	105131,3
H2A	S106	Acetylation (TSCYH)	H2AS106Acetylation (TSCYH)	14553,57	25033,56	57969,23	17346,94	71180,56	11836,28	8679,051	0	30016,18	47148,74	21206,1	28719,95	18019,09	68721,25	50437,64	29906,54	30769,23	54151,18	9593,968	30310,26
H2A	S106	Phosphorylation (STY)	H2AS106Phosphorylation (STY)	0	0	0	0	0	32411,5	0	0	0	0	0	0	0	0	0	0	0	0	0	0
H2A	R107	Citrullination	H2AR107Citrullination	0	0	30,70769	30,3207	0	0	6119,711	0	0	0	0	0	0	0	0	0	62,14896	0	48,49188	0
H2A	R107	Methylation(KR)	H2AR107Methylation(KR)	21428,57	26241,61	61353,85	18002,92	123842,6	20022,12	46130,03	0	31957,93	63492,06	49171,64	30929,17	100358	74517,02	52407	30607,48	32234,43	54894,67	16937,35	44510,74
H2A	R107	Dimethylation(KR)	H2AR107Dimethylation(KR)	0	0	683,0769	0	0	0	0	0	0	0	3247,184	0	0	0	0	0	0	0	0	0
H2A	R107	Trimethylation	H2AR107Trimethylation	1089,286	3073,826	3550,769	0	0	0	0	0	0	0	3684,559	254,0611	0	0	0	0	0	0	1612,529	0

H2A	S113	Acetylation (TSCYH)	H2AS113Acetylation (TSCYH)	0	133,557	0	0	0	0	0	0	0	0	0	0	0	0	0	0	0	0	0	0
H2A	K116	2-Hydroxyisobutyrylation	H2AK1162-Hydroxyisobutyrylation	0	133,557	0	0	0	0	0	0	0	0	0	0	0	0	0	0	0	0	0	0
H2A	K116	Acetylation (K)	H2AK116Acetylation (K)	0	0	3667,692	485,4227	0	0	0	0	0	2186,949	662,6905	0	0	1987,121	4037,199	0	0	8364,312	0	0
H2A	K120	Acetylation (K)	H2AK120Acetylation (K)	0	0	2098,462	0	0	0	0	0	0	1069,959	0	0	0	1987,121	0	0	0	0	0	0
H2A	K121	Acetylation (K)	H2AK121Acetylation (K)	0	0	1415,385	0	0	0	0	0	0	1069,959	0	0	0	1987,121	0	0	0	0	0	0
H2A	K121	Ubiquitination	H2AK121Ubiquitination	36250	0	769,2308	16326,53	163194,4	75331,86	122807	10697,67	26537,22	0	0	0	63484,49	122355,1	46936,54	69275,7	24786,32	36059,48	0	55727,92
H2A	H122	Acetylation (TSCYH)	H2AH122Acetylation (TSCYH)	0	0	0	0	0	0	0	0	0	33392,12	0	0	0	0	0	0	0	0	0	0
H2A	K123	Ubiquitination	H2AK123Ubiquitination	0	0	0	0	0	0	0	0	0	0	0	0	0	0	0	0	0	8364,312	0	0
H2A	S124	Acetylation (TSCYH)	H2AS124Acetylation (TSCYH)	0	0	1495,385	485,4227	0	0	0	0	0	1069,959	0	0	0	477,4609	2024,07	0	0	0	0	0
H2A	K127	2-Hydroxyisobutyrylation	H2AK1272-Hydroxyisobutyrylation	0	0	0	0	0	0	0	0	0	6349,206	0	4886,29	0	0	0	0	0	0	0	0
H4	K4	Acetylation (K)	H4K4Acetylation (K)	275892,9	267114,1	301538,5	334548,1	1168981	899336,3	1028896	729138,2	268608,4	49088,77	0	307342,4	2088305	63477,46	962800,9	801401,9	30036,63	0	76450,12	59069,21
H4	K10	Acetylation (K)	H4K10Acetylation (K)	24196,43	17046,98	35753,85	29883,38	0	0	80185,76	0	19417,48	49088,77	0	14814,81	0	61637,53	0	39369,16	30036,63	0	76450,12	52386,63
H4	R25	Dimethylation(KR)	H4R25Dimethylation(KR)	0	0	0	0	0	657,0796	0	0	0	0	0	0	0	0	0	0	0	0	0	0
H4	K42	Acetylation (K)	H4K42Acetylation (K)	0	1758,389	2498,462	0	0	0	2559,34	0	0	0	0	1663,418	0	0	0	2757,009	0	0	0	0
H4	R43	Methylation(KR)	H4R43Methylation(KR)	0	0	2498,462	794,4606	0	0	0	678,5226	0	1875,367	1623,592	1293,047	0	0	0	0	0	0	0	0
H4	R43	Dimethylation(KR)	H4R43Dimethylation(KR)	0	812,0805	2498,462	0	0	0	0	0	0	0	0	0	0	0	0	2757,009	0	0	0	0
H4	R43	Trimethylation	H4R43Trimethylation	0	946,3087	0	0	0	0	0	0	0	0	0	0	0	0	0	0	0	0	0	0
H4	Y49	Hydroxylation	H4Y49Hydroxylation	0	0	0	523,3236	0	0	973,1682	0	0	0	0	0	0	956,7617	0	0	0	0	0	0
H4	Y49	Acetylation (TSCYH)	H4Y49Acetylation (TSCYH)	20892,86	52684,56	52738,46	41180,76	28125	95685,84	77399,38	30232,56	22653,72	41857,73	73558,65	62768,03	38424,82	67801,29	105142,2	5899,533	51770,45	86988,85	47795,82	81980,91
H4	R53	Methylation(KR)	H4R53Methylation(KR)	20892,86	52684,56	52738,46	41180,76	28125	95685,84	77399,38	30232,56	22653,72	41857,73	73558,65	62768,03	38424,82	67801,29	105142,2	5899,533	51770,45	86988,85	47795,82	81980,91
H4	K57	Acetylation (K)	H4K57Acetylation (K)	0	0	0	0	0	0	0	0	0	0	0	0	0	312,7875	0	0	0	0	0	0
H4	K57	Methylation(KR)	H4K57Methylation(KR)	60089,29	59328,86	62769,23	56122,45	144675,9	134955,8	141382,9	5403,557	49514,56	59964,73	64214,71	75373,62	126491,6	140754,4	177242,9	156542,1	62515,26	117100,4	46287,7	139618,1
H4	S58	Acetylation (TSCYH)	H4S58Acetylation (TSCYH)	2982,143	6416,107	11200	4001,458	10694,44	6515,487	6367,389	0	6399,676	12874,78	12193,51	9291,748	13365,16	12787,49	9398,249	4637,85	4896,215	5824,04	2470,998	9701,671
H4	S58	Phosphorylation (STY)	H4S58Phosphorylation (STY)	60089,29	59328,86	62769,23	56122,45	144675,9	134955,8	141382,9	5403,557	49514,56	59964,73	64214,71	75373,62	126491,6	140754,4	177242,9	156542,1	62515,26	117100,4	44895,59	139618,1
H4	T68	Acetylation (TSCYH)	H4T68Acetylation (TSCYH)	0	406,0403	0	625,3644	171,2963	3130,531	3746,13	2585,499	0	1022,928	564,6123	1072,125	0	3707,452	2746,171	2266,355	0	0	0	1396,181
H4	T68	Ubiquitin	H4T68Ubiquitin	423,2143	0	484,3077	0	0	7710,177	0	0	81,71521	0	0	0	0	0	0	0	1233,211	5216,853	1693,735	0
H4	Y70	Hydroxylation	H4Y70Hydroxylation	0	28,05369	275,6923	0	0	279,8673	586,1713	217,5103	0	0	0	0	115,9905	539,0984	125,8206	134,3458	0	0	888,6311	170,6444
H4	Y73	Hydroxylation	H4Y73Hydroxylation	0	0	275,6923	0	0	0	190,9185	228,4542	0	0	0	0	0	307,2677	0	0	0	0	888,6311	0
H4	Y73	Acetylation (TSCYH)	H4Y73Acetylation (TSCYH)	0	0	534,1538	0	0	3042,035	3694,53	1309,166	0	1557,907	1073,559	483,4308	0	1205,152	5393,873	1810,748	0	0	0	0
H4	S74	Acetylation (TSCYH)	H4S74Acetylation (TSCYH)	24017,86	31073,83	22523,08	20699,71	30439,81	8152,655	4788,442	21203,83	30582,52	32039,98	26905,24	31448,99	52744,63	1205,152	34135,67	37967,29	26251,53	29615,86	27842,23	47613,37
H4	R75	Methylation(KR)	H4R75Methylation(KR)	56339,29	196644,3	102153,8	48032,07	30439,81	31305,31	1093,911	43775,65	53721,68	92298,65	196156,4	56530,21	81742,24	14627,41	57986,87	36098,13	164835,2	118339,5	146171,7	47613,37
H4	R75	Dimethylation(KR)	H4R75Dimethylation(KR)	0	0	534,1538	323,6152	0	3042,035	3694,53	1309,166	0	1557,907	1073,559	483,4308	0	1205,152	5393,873	1810,748	0	0	0	0
H4	K76	Methylation(KR)	H4K76Methylation(KR)	0	0	593,8462	0	10081,02	0	3261,094	0	0	834,8031	3552,021	0	13245,82	0	2385,12	3422,897	0	11288,72	0	0
H4	K76	Dimethylation(KR)	H4K76Dimethylation(KR)	1491071	1711409	2344615	1413994	3425926	2621681	2941176	1253078	1788026	2692534	2445328	2787524	2947494	3606256	3074398	2757009	2051282	2862454	1740139	2959427
H4	K77	Succinylation	H4K77Succinylation	0	0	0	0	0	0	0	0	0	0	0	0	0	0	0	0	0	0	0	0
H4	K77	Acetylation (K)	H4K77Acetylation (K)	0	0	1218,462	0	0	0	1599,587	837,2093	0	0	0	0	0	0	0	0	0	0	0	0
H4	K77	Methylation(KR)	H4K77Methylation(KR)	3473,214	0	0	0	0	137168,1	0	0	2022,654	0	0	0	14916,47	0	8205,689	3422,897	0	21065,68	0	2100,239
H4	K77	Dimethylation(KR)	H4K77Dimethylation(KR)	4071,429	3348,993	2713,846	22886,3	10312,5	6393,805	0	5512,996	3600,324	1322,751	2849,569	0	0	0	0	0	2771,673	0	2691,415	0
H4	K77	Trimethylation	H4K77Trimethylation	0	0	0	1137,026	561,3426	227,8761	137254,9	62243,5	0	3115,814	0	0	13723,15	6329,347	154267	149532,7	0	0	0	140811,5
H4	T78	Acetylation (TSCYH)	H4T78Acetylation (TSCYH)	919,6429	640,2685	2387,692	947,5219	3020,833	0	514,9639	0	762,945	0	1457,919	0	4033,413	0	3118,162	662,3832	0	971,4994	674,0139	3544,153
H4	T80	Acetylation (TSCYH)	H4T80Acetylation (TSCYH)	0	0	0	0	0	0	0	0	0	0	473,8237	0	0	0	0	0	0	0	0	0
H4	R89	Citrullination	H4R89Citrullination	14642,86	9932,886	19446,15	13119,53	33796,3	13384,96	20433,44	9712,722	23543,69	18753,67	8614,977	14165,04	22792,36	35326,59	12363,24	16471,96	10549,45	18215,61	5382,831	17064,44
H4	K90	Acetylation (K)	H4K90Acetylation (K)	6589,286	9261,745	4763,077	10349,85	23958,33	16150,44	14860,68	7510,26	9951,456	14520,87	13055	2443,145	0	19687,21	15098,47	2021,028	22710,62	0	19025,52	2995,227
H4	R91	Methylation(KR)	H4R91Methylation(KR)	0	0	3489,231	0	0	0	792,5697	0	0	0	0	10851,2	0	0	0	20443,93	0	18463,44	0	12529,83
H4	R91	Dimethylation(KR)	H4R91Dimethylation(KR)	0	2798,658	9661,538	736,1516	0	2278,761	2518,06	5731,874	1148,867	1040,564	7886,017	6952,567	0	1195,952	7757,112	0	1294,261	6530,359	2563,805	0
H4	R91	Trimethylation	H4R91Trimethylation	0	0	4763,077	2048,105	0	2477,876	0	1614,227	2022,654	3256,908	1855,533	1669,916	0	0	4474,836	9801,402	0	0	0	2995,227
H4	K93	Acetylation (K)	H4K93Acetylation (K)	0	0	4763,077	0	0	2477,876	0	0	2022,654	0	0	1669,916	0	0	1859,956	2021,028	0	0	0	0
H4	K93	Methylation(KR)	H4K93Methylation(KR)	0	0	0	0	0	0	792,5697	0	0	0	0	0	0	0	0	0	0	0	0	0
H4	K93	Trimethylation	H4K93Trimethylation	0	0	0	7507,289	0	11393,81	0	0	3438,511	10229,28	5520,212	0	0	10855,57	0	0	0	18463,44	0	0
H3V	K42	Dimethylation(KR)	H3VK42Dimethylation(KR)	0	0	0	0	0	0	0	0	0	0	0	0	0	0	0	70,3271	0	0	0	0
H3V	K43	Dimethylation(KR)	H3VK43Dimethylation(KR)	0	0	0	0	0	0	0	0	0	0	0	0	0	1205,152	0	0	0	0	0	1229,117
H3V	K43	Trimethylation	H3VK43Trimethylation	0	0	0	0	2511,574	1814,159	0	1969,904	0	0	0	0	0	0	900,4376	1355,14	0	0	0	847,2554
H3V	S45	Acetylation (TSCYH)	H3VS45Acetylation (TSCYH)	6160,714	8791,946	8246,154	4147,23	19791,67	14712,39	5221,878	0	7176,375	4115,226	6302,187	6627,68	9642,005	6991,72	9540,481	755,8411	7521,368	11276,33	5707,657	0
H3V	S45	Phosphorylation (STY)	H3VS45Phosphorylation (STY)	3098,214	0	1464,615	1486,88	0	616,1504	6491,228	4227,086	0	1793,063	0	2124,756	13245,82	13707,45	5820,569	0	7582,418	0	3700,696	0
H3V	T50	Acetylation (TSCYH)	H3VT50Acetylation (TSCYH)	0	0	0	0	0	0	0	0	0	355,6731	0	0	0	0	0	0	0	0	0	0
H3V	R51	Methylation(KR)	H3VR51Methylation(KR)	2616,071	0	0	0	0	0	6418,989	6484,268	2896,44	7760,141	3724,321	1903,834	7446,301	10671,57	0	3422,897	5518,926	10879,8	4814,385	18019,09
H3V	K79	Dimethylation(KR)	H3VK79Dimethylation(KR)	0	0	0	0	0	0	0	0	0	0	0	0	0	0	0	0	1096,459	0	0	0

H3V	R83	Dimethylation(KR)	H3VR83Dimethylation(KR)	0	0	0	0	0	0	0	0	0	0	0	0	0	0	0	0	1096,459	0	0	0
H3V	K94	Dimethylation(KR)	H3VK94Dimethylation(KR)	8312,5	8859,06	10523,08	6078,717	26041,67	15044,25	19401,44	7879,617	8576,052	11111,11	10006,63	9616,634	10656,32	18859,25	17943,11	4240,654	13553,11	31350,68	6392,111	15751,79
H3V	K94	Trimethylation	H3VK94Trimethylation	19821,43	8657,718	35138,46	10568,51	53703,7	9391,593	57481,94	9343,365	45469,26	28042,33	34062,29	34957,76	38902,15	63017,48	27899,34	24415,89	34920,63	33581,16	33178,65	37947,49
H3V	Y135	Acetylation (TSCYH)	H3VY135Acetylation (TSCYH)	0	0	58,70769	0	0	0	0	0	0	0	159,7084	0	0	0	0	0	0	0	0	0
H3V	Y135	Phosphorylation (STY)	H3VY135Phosphorylation (STY)	0	0	0	0	0	0	0	0	0	0	1027,17	0	0	0	0	0	0	0	0	0
H3V	K137	Methylation(KR)	H3VK137Methylation(KR)	0	0	0	0	0	0	0	0	0	0	1027,17	0	0	0	0	0	0	0	0	0
H3V	K144	Acetylation (K)	H3VK144Acetylation (K)	0	0	0	0	0	0	0	0	0	3109,935	0	5438,596	0	0	0	0	0	0	0	0
H2AZ	K31	Acetylation (K)	H2AZK31Acetylation (K)	601,7857	0	0	0	0	0	0	0	0	0	0	222,2222	0	0	0	0	0	2118,959	1039,443	0
H2AZ	K35	Acetylation (K)	H2AZK35Acetylation (K)	601,7857	0	0	0	2986,111	0	0	0	0	0	552,6839	682,2612	0	1021,159	0	0	0	0	0	0
H2AZ	K54	Acetylation (K)	H2AZK54Acetylation (K)	3625	1416,107	2073,846	998,5423	7731,481	37389,38	14963,88	38850,89	622,1683	202,8219	182,9026	402,859	8687,351	18307,27	20897,16	34696,26	2319,902	43246,59	9976,798	36396,18
H2AZ	K58	Acetylation (K)	H2AZK58Acetylation (K)	2258,929	4845,638	3772,308	660,3499	7731,481	34955,75	15170,28	36525,31	1755,663	699,5885	85,48708	103,9636	8687,351	22447,1	20240,7	21845,79	5995,116	41140,02	18329,47	35680,19
H2BV	K117	2-Hydroxyisobutyrylation	H2BVK1172-Hydroxyisobutyrylation	891,0714	0	0	0	0	0	0	0	0	0	0	0	0	0	0	0	0	0	0	0
H2BV	K125	2-Hydroxyisobutyrylation	H2BVK1252-Hydroxyisobutyrylation	0	0	0	0	0	0	0	0	0	0	0	11436	0	0	0	0	0	0	0	0
H1	K19	Acetylation (K)	H1K19Acetylation (K)	22857,14	64429,53	33292,31	20481,05	6238,426	133849,6	76780,19	80848,15	26132,69	85831,86	20212,06	19298,25	28639,62	124195	598,4683	52102,8	24175,82	167286,2	206496,5	165871,1
H1	S21	Phosphorylation (STY)	H1S21Phosphorylation (STY)	2053,571	9261,745	5156,923	1508,746	0	0	25387	0	1723,301	8935,92	1438,038	1663,418	0	0	0	0	0	0	5406,032	8198,091
H1	K33	Succinylation	H1K33Succinylation	0	0	0	0	0	0	0	0	0	0	0	0	0	0	0	0	0	18835,19	0	0

Table S10: Ratio of PTM areas, knockout/WT

							At least 2 x enrichment in the acetylation values of KO metacyclic trypomastigotes compared to WT metacyclic trypomastigotes											Acetylation values in KO metacyclic trypomastigotes ≥ acetylation values in KO epimastigotes			Putative target
			run 2		run 1		meta KO/WT		BOTH runs	run 2				run 1				KO meta/epi		BOTH runs	BOTH conditions
			META WT1	META KO1	META WT5	META KO5	run 2	run 1		EPI KO1	EPI KO2	EPI KO3	EPI KO4	EPI KO5	EPI KO6	EPI KO7	EPI KO8	run 2	run 1		
H2A	T85	Acetylation (TSCYH)	2136,7521	4605,5684	0	10357,995	2,155406032	#DIV/0!	H2AT85ac	1221,683	4726,631	5619,616	4152,047	4498,81	7589,7	4332,6	0	1,171902107	2,523093043	H2AT85ac	H2AT85ac
H4	K10	Acetylation (K)	30036,63	76450,116	0	52386,635	2,545229472	#DIV/0!	H4K10ac	19417,48	49088,77	0	14814,81	0	61637,5	0	39369,2	3,670146026	2,074580727	H4K10ac	H4K10ac
H4	T78	Acetylation (TSCYH)	0	674,01392	971,49938	3544,1527	#DIV/0!	3,648126613	H4T78ac	762,945	0	1457,919	0	4033,41	0	3118,16	662,383	1,213966961	1,814267621	H4T78ac	H4T78ac
H4	K4	Acetylation (K)	30036,63	76450,116	0	59069,212	2,545229472	#DIV/0!	H4K4ac	268608,4	49088,77	0	307342,4	2088305	63477,5	962801	801402	0,489249731	0,060336495		
H2B	T24	Acetylation (TSCYH)	48107,448	53364,269	130111,52	59785,203	1,109272498	0,459491988		29288,03	34567,9	41948,31	75373,62	56205,3	53265,9	8905,91	0	1,178163163	2,020162318	H2BT24ac	
H2AZ	K54	Acetylation (K)	2319,9023	9976,7981	43246,592	36396,181	4,300525095	0,841596515		622,1683	202,8219	182,9026	402,859	8687,35	18307,3	20897,2	34696,3	28,28789184	1,762782278	H2AZK54ac	
H2AZ	K58	Acetylation (K)	5995,116	18329,466	41140,025	35680,191	3,057399785	0,867286569		1755,663	699,5885	85,48708	103,9636	8687,35	22447,1	20240,7	21845,8	27,72253692	1,949179415	H2AZK58ac	
H1	K19	Acetylation (K)	24175,824	206496,52	167286,25	165871,12	8,541446952	0,991540705		26132,69	85831,86	20212,06	19298,25	28639,6	124195	598,468	52102,8	5,452958327	3,228070691	H1K19ac	
H2B	S32	Acetylation (TSCYH)	0	0	0	8579,9523	#DIV/0!	#DIV/0!		1618,123	11111,11	821,7362	0	0	0	18599,6	0	0	1,845194441		
H2B	S41	Acetylation (TSCYH)	8717,9487	758,7007	0	10942,721	0,087027433	#DIV/0!		0	11757,79	0	0	0	10763,6	0	0	0,258109977	4,066576913		
H2B	T46	Acetylation (TSCYH)	0	0	0	3,8544153	#DIV/0!	#DIV/0!		0	0	0	12,47563	379,475	37,0745	0	0	0	0,037012798		
H2B	T67	Acetylation (TSCYH)	3492,0635	10336,427	9157,373	0	2,959976798	0		5647,249	8641,975	8018,555	3339,831	0	13799,4	9584,25	11425,2	1,612068579	0		
H2B	H97	Acetylation (TSCYH)	1067,1551	0	990,08674	30190,931	0	30,49321795		0	0	22531,48	0	72195,7	793,928	0	0	0	1,65453256		
H2B	T103	Acetylation (TSCYH)	75702,076	660092,81	1363073,1	110501,19	8,719613053	0,081067694		182038,8	201058,2	1610338	955165,7	264916	1481141	959519	121495	0,895465849	0,156347241		
H2B	S43	Acetylation (TSCYH)	39804,64	8225,058	81164,808	43556,086	0,20663566	0,536637578		50080,91	113462,7	52683,9	7537,362	16229,1	64029,4	48030,6	7348,13	0,147030396	1,284486763		
H3	K20	Acetylation (K)	0	0	0	1193,3174	#DIV/0!	#DIV/0!		0	0	0	0	0	0	59518,6	52453,3	#DIV/0!	0,042629186		
H3	K61	Acetylation (K)	2039,072	1160,0928	1833,9529	3066,8258	0,568931742	1,67224892		0	2657,26	735,5865	2339,181	1503,58	1554,74	3041,58	4415,89	0,80955136	1,166561283		
H2A	S19	Acetylation (TSCYH)	38583,639	23201,856	45972,739	43914,081	0,601339246	0,95522004		30582,52	40681,95	49900,6	52566,6	39140,8	53909,8	47155,4	29088,8	0,534199794	1,037576602		
H2A	S33	Acetylation (TSCYH)	1636,1416	2540,6032	0	3365,1551	1,552801538	#DIV/0!		1860,841	168,7243	302,8496	5224,172	2971,36	6007,36	2593	5280,37	1,344841692	0,798750727		
H2A	H92	Acetylation (TSCYH)	2576,3126	3410,6729	3469,6406	13603,819	1,323858326	3,920814865		11569,58	13639,04	2776,673	1630,929	32816,2	9751,61	0	5981,31	0,460649351	1,12082863		
H4	Y49	Acetylation (TSCYH)	51770,452	47795,824	86988,848	81980,907	0,923225934	0,942430084		22653,72	41857,73	73558,65	62768,03	38424,8	67801,3	105142	5899,53	0,951927271	1,509305643		
H4	T68	Acetylation (TSCYH)	0	0	0	1396,1814	#DIV/0!	#DIV/0!		0	1022,928	564,6123	1072,125	0	3707,45	2746,17	2266,36	0	0,640451827		
H4	S74	Acetylation (TSCYH)	26251,526	27842,227	29615,861	47613,365	1,060594615	1,607698146		30582,52	32039,98	26905,24	31448,99	52744,6	1205,15	34135,7	37967,3	0,92058126	1,510902993		
H4	K90	Acetylation (K)	22710,623	19025,522	0	2995,2267	0,837736696	#DIV/0!		9951,456	14520,87	13055	2443,145	0	19687,2	15098,5	2021,03	1,903957582	0,325508781		
H2AZ	K31	Acetylation (K)	0	1039,4432	2118,9591	0	#DIV/0!	0		0	0	0	222,2222	0	0	0	0	18,7099768	#DIV/0!		
H2B	K20	Acetylation (K)	0	0	0	0	#DIV/0!	#DIV/0!		0	0	0	0	0	0	19365,4	40654,2	#DIV/0!	0		
H2B	K22	Acetylation (K)	0	0	0	0	#DIV/0!	#DIV/0!		0	0	0	0	0	0	19365,4	40654,2	#DIV/0!	0		
H2B	S30	Acetylation (TSCYH)	46642,247	41183,295	106195,79	103579,95	0,882961213	0,975367812		56148,87	81716,64	72233,27	48862,9	115871	134315	122538	49766,4	0,636129582	0,98066089		
H2B	K34	Acetylation (K)	0	0	188,35192	110,26253	#DIV/0!	0,585406984		0	0	0	0	0	0	0	0	#DIV/0!	#DIV/0!		
H2B	S35	Acetylation (TSCYH)	0	0	1734,8203	0	#DIV/0!	0		0	0	650,7621	0	0	0	0	0	0	#DIV/0!		
H2B	K48	Acetylation (K)	1098,9011	0	0	0	0	#DIV/0!		0	0	2379,059	0	0	0	71,116	0	0	0		
H2B	S52	Acetylation (TSCYH)	0	0	0	0	#DIV/0!	#DIV/0!		0	0	0	0	0	0	0	0	#DIV/0!	#DIV/0!		
H2B	K73	Acetylation (K)	0	0	0	0	#DIV/0!	#DIV/0!		0	0	0	0	0	0	0	0	#DIV/0!	#DIV/0!		
H2B	K74	Acetylation (K)	0	0	0	0	#DIV/0!	#DIV/0!		0	0	0	0	0	0	0	0	#DIV/0!	#DIV/0!		
H2B	T76	Acetylation (TSCYH)	0	0	0	0	#DIV/0!	#DIV/0!		0	946,5021	0	760,2339	0	0	0	0	0	#DIV/0!		
H2B	K96	Acetylation (K)	0	0	0	0	#DIV/0!	#DIV/0!		0	0	0	0	584,726	565,777	0	0	#DIV/0!	0		
H2B	S107	Acetylation (TSCYH)	5970,696	0	0	0	0	#DIV/0!		0	0	0	0	0	0	0	0	#DIV/0!	#DIV/0!		
H2B	H108	Acetylation (TSCYH)	5970,696	0	0	0	0	#DIV/0!		0	0	0	0	0	0	0	0	#DIV/0!	#DIV/0!		
H3	T29	Acetylation (TSCYH)	492,06349	0	0	0	0	#DIV/0!		470,8738	593,7684	600,3976	656,2703	0	1517,94	1126,91	0	0	0		
H3	T55	Acetylation (TSCYH)	0	0	0	0	#DIV/0!	#DIV/0!		0	1205,173	0	0	0	0	0	0	0	#DIV/0!		
H3	S72	Acetylation (TSCYH)	0	0	0	0	#DIV/0!	#DIV/0!		0	0	0	0	0	0	0	0	#DIV/0!	#DIV/0!		
H3	C126	Acetylation (TSCYH)	168498,17	68909,513	128872,37	50119,332	0,408962978	0,388906738		122168,3	289829,5	456593,8	385964,9	85560,9	64213,4	88840,3	58411,2	0,219709559	0,674949275		
H2A	K21	Acetylation (K)	1040,293	1809,7448	0	0	1,739649031	#DIV/0!		1949,838	2557,319	2365,805	2001,3	0	0	2593	0	0,815727439	0		
H2A	K101	Acetylation (K)	495,7265	569,60557	0	0	1,149031923	#DIV/0!		1189,32	1622,575	1338,635	478,2326	0	2474,7	657,549	720,794	0,492231372	0		
H2A	T104	Acetylation (TSCYH)	0	0	0	0	#DIV/0!	#DIV/0!		0	0	0	0	0	0	0	0	#DIV/0!	#DIV/0!		
H2A	S106	Acetylation (TSCYH)	30769,231	9593,9675	54151,177	30310,263	0,311803944	0,559734139		30016,18	47148,74	21206,1	28719,95	18019,1	68721,3	50437,6	29906,5	0,301955933	0,725627053		
H2A	S113	Acetylation (TSCYH)	0	0	0	0	#DIV/0!	#DIV/0!		0	0	0	0	0	0	0	0	#DIV/0!	#DIV/0!		
H2A	K116	Acetylation (K)	0	0	8364,3123	0	#DIV/0!	0		0	2186,949	662,6905	0	0	1987,12	4037,2	0	0	0		
H2A	K120	Acetylation (K)	0	0	0	0	#DIV/0!	#DIV/0!		0	1069,959	0	0	0	1987,12	0	0	0	0		
H2A	K121	Acetylation (K)	0	0	0	0	#DIV/0!	#DIV/0!		0	1069,959	0	0	0	1987,12	0	0	0	0		

H2A	H122	Acetylation (TSCYH)	0	0	0	0	#DIV/0!	#DIV/0!	0	33392,12	0	0	0	0	0	0	0	#DIV/0!
H2A	S124	Acetylation (TSCYH)	0	0	0	0	#DIV/0!	#DIV/0!	0	1069,959	0	0	0	477,461	2024,07	0	0	0
H4	K42	Acetylation (K)	0	0	0	0	#DIV/0!	#DIV/0!	0	0	0	1663,418	0	0	0	2757,01	0	0
H4	K57	Acetylation (K)	0	0	0	0	#DIV/0!	#DIV/0!	0	0	0	0	0	312,787	0	0	#DIV/0!	0
H4	S58	Acetylation (TSCYH)	4896,2149	2470,9977	5824,0397	9701,6706	0,504675087	1,665797491	6399,676	12874,78	12193,51	9291,748	13365,2	12787,5	9398,25	4637,85	0,242494141	0,965610744
H4	Y73	Acetylation (TSCYH)	0	0	0	0	#DIV/0!	#DIV/0!	0	1557,907	1073,559	483,4308	0	1205,15	5393,87	1810,75	0	0
H4	K77	Acetylation (K)	0	0	0	0	#DIV/0!	#DIV/0!	0	0	0	0	0	0	0	0	#DIV/0!	#DIV/0!
H4	T80	Acetylation (TSCYH)	0	0	0	0	#DIV/0!	#DIV/0!	0	0	473,8237	0	0	0	0	0	0	#DIV/0!
H4	K93	Acetylation (K)	0	0	0	0	#DIV/0!	#DIV/0!	2022,654	0	0	1669,916	0	0	1859,96	2021,03	0	0
H3V	S45	Acetylation (TSCYH)	7521,3675	5707,6566	11276,332	0	0,758858891	0	7176,375	4115,226	6302,187	6627,68	9642	6991,72	9540,48	755,841	0,942578112	0
H3V	T50	Acetylation (TSCYH)	0	0	0	0	#DIV/0!	#DIV/0!	0	355,6731	0	0	0	0	0	0	0	#DIV/0!
H3V	Y135	Acetylation (TSCYH)	0	0	0	0	#DIV/0!	#DIV/0!	0	0	159,7084	0	0	0	0	0	0	#DIV/0!
H3V	K144	Acetylation (K)	0	0	0	0	#DIV/0!	#DIV/0!	0	3109,935	0	5438,596	0	0	0	0	0	#DIV/0!
H2AZ	K35	Acetylation (K)	0	0	0	0	#DIV/0!	#DIV/0!	0	0	552,6839	682,2612	0	1021,16	0	0	0	0

Table S11: Sequences of the oligonucleotides used in this work

Primer name	*Fragment*	*Sequence (5' – 3')*
HDAC4_F	HDAC4 coding sequence	ggggacaagtttgtacaaaaaagcaggctggATGAAGAAGCGGGGGCCTCAGC
HDAC4_R		ggggaccactttgtacaagaaagctgggtcTTGCCTTTTGTTCGACTCCTCG
DAC4UpsF	HDAC4 5'flank	CTTCTGGTACCGATTTTATTGGGTTTCAG
DAC4UpsR		TTCCGTCGACAGCAACATGCGTGAATCGTACG
DAC4DownF	HDAC4 3'flank	AAGGGATCCCAAAGCTCCTTCACTGCTTGCC
DAC4DownR		TTCTCTAGACCCCACACTCCACTGGTTCTTTTG
DAC4ExtF	HDAC4 locus	GTTGAGGTGTCACTTTGTATTGCAG
DAC4ExtR		CACATCATCTCTGGCGGTGG

AttB sites for DNA recombination are in lowercase. Restriction sites are underlined.

Complete Images

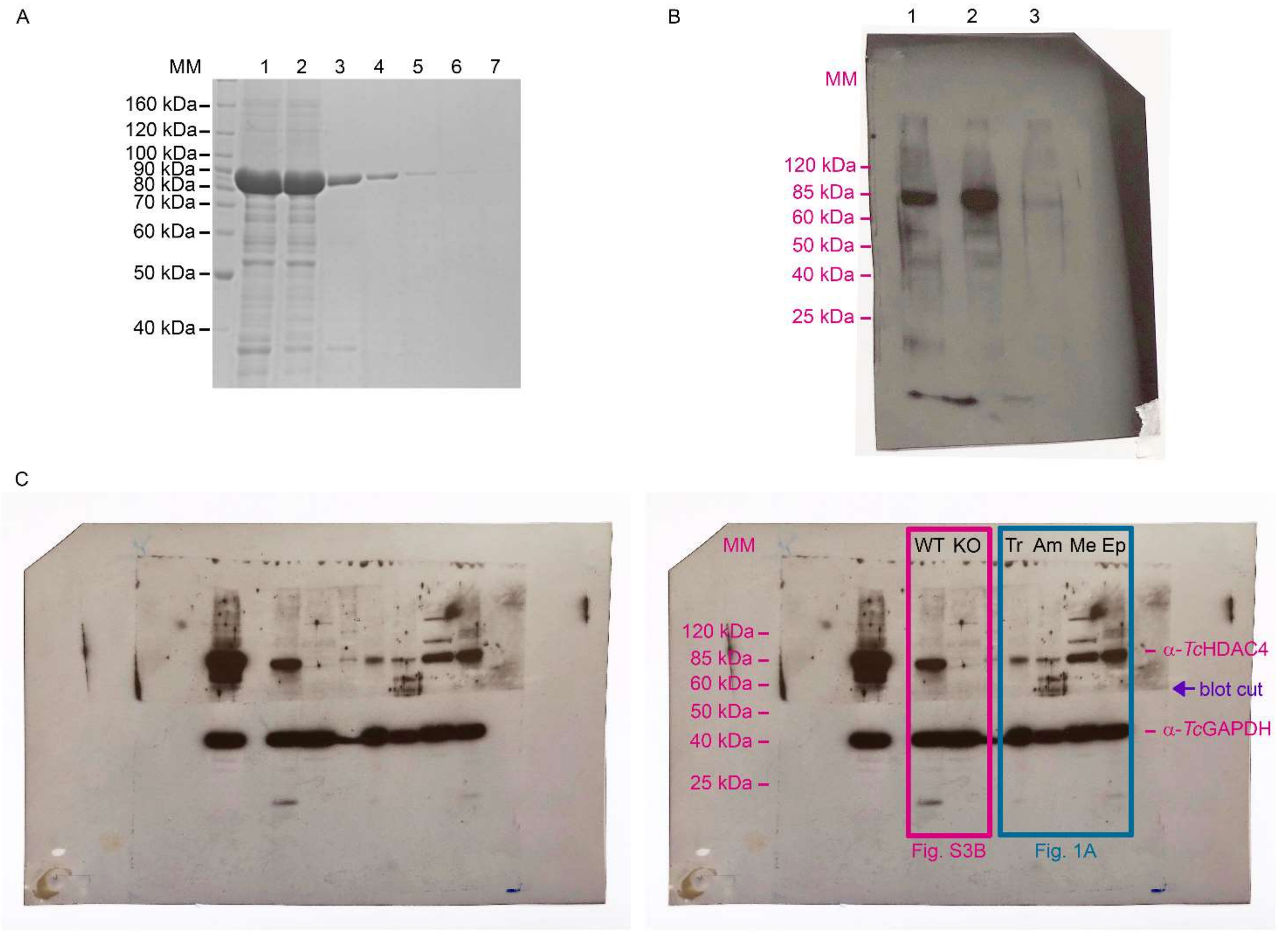

Figure CI-1. **A.** SDS-PAGE of fractions (1-7) obtained from a recombinant *Tc*HDAC4 purification showing that it presents a slower electrophoretic mobility than predicted from its molecular mass, migrating in the range of ~85 kDa. MM: BenchMark Protein Ladder (Invitrogen). **B.** Original image of a western blot developed by chemiluminescence using three different non-normalized *T. cruzi* epimastigote extracts and an antiserum for *Tc*HDAC4. The blot shows a specific recognition of *Tc*HDAC4 by the antiserum with *Tc*HDAC4 showing electrophoretic mobility in the same range of the recombinant *Tc*HDAC4. MM: Pre-stained BenchMark Protein Ladder (Invitrogen). **C.** Complete image of the western blot developed by chemiluminescence partially shown in figures 1A and S3B, using antisera for *Tc*HDAC4 and *Tc*GAPDH. After transfer, the membrane was cut and incubated separately with the *Tc*HDAC4 and *Tc*GAPDH antisera. For development by chemiluminescence, the membrane parts were placed back together to reconstitute the complete blot image. The parts shown in the figures 1A and S3B are marked by boxes in the image on the right. *Tc*GAPDH was used as a loading control for the cell extracts [epimastigote (Ep), metacyclic trypomastigote (Me), amastigote (Am) and cell culture-derived trypomastigote (Tr) cells]. The polyclonal mouse antibody against *T. cruzi* glyceraldehyde 3-phosphate dehydrogenase (GAPDH) was kindly provided by F. Morini [Carlos Chagas Institute, Oswaldo Cruz Foundation (Fiocruz), state of Paraná, Brazil]. MM: Pre-stained BenchMark Protein Ladder (Invitrogen).

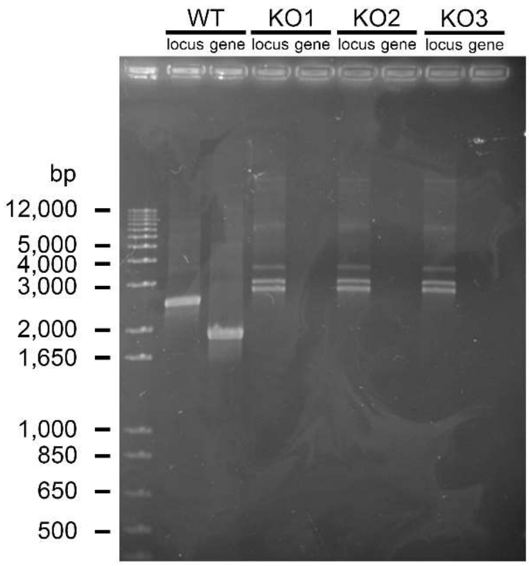

Figure CI-2. Complete image of the DNA gel electrophoresis shown in figure 3E. Gel electrophoresis of the PCR products of the analyses performed for confirmation of *Tc*HDAC4 knockout. MM: 1 Kb Plus DNA Ladder, Invitrogen.

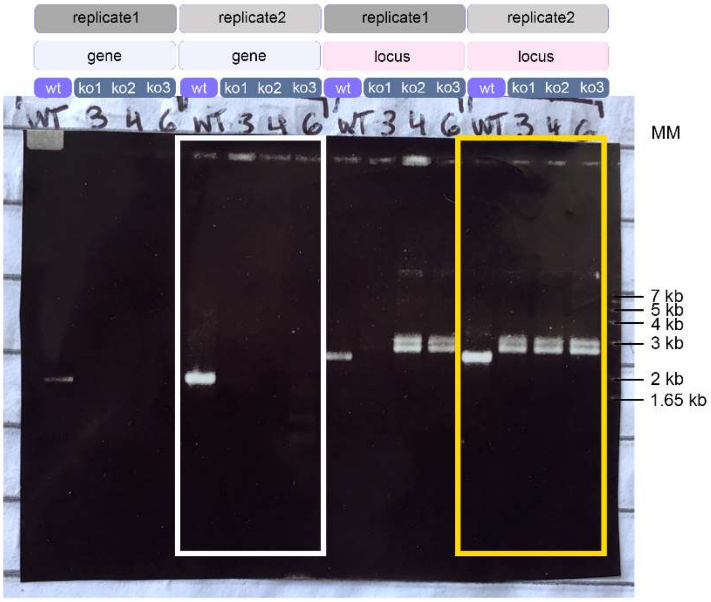

Figure CI-3. Complete image of the DNA gel electrophoresis shown in figure S3C. The image shows the analysis of the PCR products performed for confirmation of *Tc*HDAC4 knockout. The lanes identified by "gene" correspond to the PCR reactions performed using oligonucleotides DAC4UpsF + DAC4R (Table S11). A band showing amplification of the gene is seen only in the wild type sample. The lanes identified by "locus" correspond to the PCR reactions performed using primers DAC4ExtF + DAC4ExtR (Table S11). The single band in the WT sample indicates the presence of the original genome sequence. The two bands in the KO samples represent each of the selection makers used to knockout the two copies of the *Tc*HDAC4 gene. The white and yellow boxes mark the regions shown in the figure S3C. MM: 1 Kb Plus DNA Ladder, Invitrogen.
